# How culturally unique are pandemic effects? Evaluating cultural similarities and differences in effects of age, biological sex, and political beliefs on COVID impacts

**DOI:** 10.3389/fpsyg.2022.937211

**Published:** 2022-12-19

**Authors:** Lucian Gideon Conway, Shailee R. Woodard, Alivia Zubrod, Marcela Tiburcio, Nora Angélica Martínez-Vélez, Angela Sorgente, Margherita Lanz, Joyce Serido, Rimantas Vosylis, Gabriela Fonseca, Žan Lep, Lijun Li, Maja Zupančič, Carla Crespo, Ana Paula Relvas, Kostas A. Papageorgiou, Foteini-Maria Gianniou, Tayler Truhan, Dara Mojtahedi, Sophie Hull, Caroline Lilley, Derry Canning, Esra Ulukök, Adnan Akın, Claudia Massaccesi, Emilio Chiappini, Riccardo Paracampo, Sebastian Korb, Magdalena Szaflarski, Almamy Amara Touré, Lansana Mady Camara, Aboubacar Sidiki Magassouba, Abdoulaye Doumbouya, Melis Mutlu, Zeynep Nergiz Bozkurt, Karolina Grotkowski, Aneta M. Przepiórka, Nadia Saraí Corral-Frías, David Watson, Alejandro Corona Espinosa, Marc Yancy Lucas, Francesca Giorgia Paleari, Kristina Tchalova, Amy J. P. Gregory, Talya Azrieli, Jennifer A. Bartz, Harry Farmer, Simon B. Goldberg, Melissa A. Rosenkranz, Jennifer Pickett, Jessica L. Mackelprang, Janessa M. Graves, Catherine Orr, Rozel Balmores-Paulino

**Affiliations:** ^1^Department of Psychology, University of Montana, Missoula, MT, United States; ^2^Department of Psychology, Keene State College, Keene, NH, United States; ^3^Department of Psychology and Sociology, Park University, Parkville, MO, United States; ^4^Departamento de Ciencias Sociales en Salud, Instituto Nacional de Psiquiatría Ramón de la Fuente Muñiz, Mexico City, Mexico; ^5^Unità di Ricerca Teoria della Mente, Dipartimento di Psicologia, Università Cattolica del Sacro Cuore, Milan, Italy; ^6^University of Minnesota Twin Cities, St. Paul, MN, United States; ^7^Mykolas Romeris University, Vilnius, Lithuania; ^8^Faculty of Psychology and Education Sciences, Centre for Social Studies, University of Coimbra, Coimbra, Portugal; ^9^University of Ljubljana, Ljubljana, Slovenia; ^10^University of Lisbon, Lisbon, Portugal; ^11^University of Coimbra, Coimbra, Portugal; ^12^Queen’s University Belfast, Belfast, United Kingdom; ^13^University of Huddersfield, Huddersfield, United Kingdom; ^14^Department of Business Administration, Kırıkkale University, Kırıkkale, Turkey; ^15^Department of Clinical and Health Psychology, Faculty of Psychology, University of Vienna, Vienna, Austria; ^16^Netherlands Institute for Neuroscience (KNAW), Amsterdam, Netherlands; ^17^Department of Psychology, University of Essex, Colchester, United Kingdom; ^18^University of Alabama at Birmingham, Birmingham, AL, United States; ^19^Department of Medical Sciences, Kofi Annan University of Guinea, Conakry, Guinea; ^20^National Centre of Training and Recherche in Rural Health of Mafèrinyah, Forécariah, Guinea; ^21^Department of Public Health, Faculty of Sciences and Health Techniques, Gamal Abdel Nasser University, Conakry, Guinea; ^22^Erasmus University Rotterdam, Rotterdam, Netherlands; ^23^Cognitive Neuropsychology Master’s Program, Institute of Graduate Education, Bahçeşehir University, Istanbul, Turkey; ^24^Rosalind Franklin University of Medicine and Science, North Chicago, IL, United States; ^25^The John Paul II Catholic University of Lublin, Lublin, Poland; ^26^Department of Psychology, University of Sonora, Hermosillo, Mexico; ^27^University of Notre Dame, Notre Dame, IN, United States; ^28^Department of Human and Social Sciences, University of Bergamo, Bergamo, Italy; ^29^Department of Psychology, McGill University, Montreal, QC, Canada; ^30^Institute for Lifecourse Development, University of Greenwich, London, United Kingdom; ^31^Department of Counseling Psychology and Center for Healthy Minds, University of Wisconsin – Madison, Madison, WI, United States; ^32^Department of Psychiatry and Center for Healthy Minds, University of Wisconsin – Madison, Madison, WI, United States; ^33^Vrije Universiteit Brussel, Brussels, Belgium; ^34^Swinburne University of Technology, Hawthorn, VIC, Australia; ^35^Washington State University – Spokane, Spokane, WA, United States; ^36^Department of Anthropology, Sociology and Psychology College of Social Sciences, University of the Philippines Baguio, Baguio, Philippines

**Keywords:** COVID-19, cultural psychology, age, biological sex, political beliefs, cross-cultural psychology, pandemic psychology, adverse psychological change

## Abstract

Despite being bio-epidemiological phenomena, the causes and effects of pandemics are culturally influenced in ways that go beyond national boundaries. However, they are often studied in isolated pockets, and this fact makes it difficult to parse the unique influence of specific cultural psychologies. To help fill in this gap, the present study applies existing cultural theories *via* linear mixed modeling to test the influence of unique cultural factors in a multi-national sample (that moves beyond Western nations) on the effects of age, biological sex, and political beliefs on pandemic outcomes that include adverse financial impacts, adverse resource impacts, adverse psychological impacts, and the health impacts of COVID. Our study spanned 19 nations (participant *N* = 14,133) and involved translations into 9 languages. Linear mixed models revealed similarities across cultures, with both young persons and women reporting worse outcomes from COVID across the multi-national sample. However, these effects were generally qualified by culture-specific variance, and overall more evidence emerged for effects unique to each culture than effects similar across cultures. Follow-up analyses suggested this cultural variability was consistent with models of pre-existing inequalities and socioecological stressors exacerbating the effects of the pandemic. Collectively, this evidence highlights the importance of developing culturally flexible models for understanding the cross-cultural nature of pandemic psychology beyond typical WEIRD approaches.

## Introduction

By definition, a pandemic is a worldwide spread of a new disease with social and psychological implications that also crosses cultural and national boundaries (World Health Organization, 2010). In order to fully understand the psychology of pandemics such as the worldwide spread of the new coronavirus (i.e., SARS-CoV-2) causing a COVID-19 outbreak (classified as a pandemic by the on 11 March; World Health Organization, 2020a,b), researchers cannot merely study individual nations or isolated locales. Rather, we need an increasing number of multi-national studies that evaluate the cultural psychology of pandemics around the world (De Backer et al., 2021; Motrico et al., 2021; Blackburn and Vestergren, 2022; Legate et al., 2022).

Indeed, this is especially important given the tendency in psychology to focus exclusively on WEIRD (Western, Educated, Industrialized, Rich, and Democratic) samples (Henrich et al., 2010). For example, COVID-19 has particular implications for Asia and Asian psychology, and that is likely why Asian social psychologists have taken an especially keen interest in the pandemic (Khazaie and Khan, 2020; Albarracin and Jung, 2021; Bond, 2021; Jetten et al., 2021; Kashima, 2021; Liu, 2021). These researchers have highlighted the dangers inherent in attempting to understand the pandemic without considering the unique cultures inherent in each locale – and in particular Asian national locales (see, e.g., Bond, 2021; Kashima, 2021; Liu, 2021). For example, as Liu (2021) notes, there was a strong tendency for Western scholars to ignore the success of many Asian countries in fighting the pandemic because that success was in part due to cultural variability in collectivism less instantiated in the West. Cultural variability is vital to our understanding of pandemic psychology.

Thus, one of the important questions to consider when evaluating the psychology of the pandemic world-wide is the degree that particular effects can be explained by culture-specific mechanisms. In the present research, a group of collaborators from around the world – including many non-WEIRD contexts – used linear mixed models to evaluate the degree that effects of age, biological sex, and political beliefs involved shared variance across cultures versus variance unique to each culture. We evaluate outcomes that include adverse financial impacts, adverse resource impacts, adverse psychological impacts, and the health impacts of COVID. We then use existing theory to further investigate *why* different cultures might show different effects. This investigation represents 14,133 participants across 6 continents, with data from 19 nations and scale translations into 9 languages.

Importantly, while in each case some prior research suggests relationships between our independent variables and COVID psychological outcomes, our work – over and above this prior work – allows for simultaneous comparisons of shared versus unique cultural variance in the effects of age, biological sex, and political beliefs. Most prior work involves studying isolated pockets and no work that we know of has attempted a comprehensive study of the effects of these variables on identical measures validated for use in those nations. Thus, our work makes a novel contribution to a broad cultural psychological understanding of pandemics by evaluating the cultural contribution of the effects of biological sex and age on adverse financial impacts, adverse resource impacts, adverse psychological impacts, and the health impacts of COVID.

## Age and biological sex across cultures: Structural inequality and socioecological stress theories

At a broad level, much theory suggests that events such as pandemics expose societal vulnerabilities and inequalities regarding access to resources, capabilities, and opportunities (Boin et al., 2016; Connor et al., 2020; Politi et al., 2021). Consistent with this, COVID-19 research suggests that women (e.g., Ausín et al., 2020) and younger persons (e.g., Vahia et al., 2020) are especially vulnerable to the psychological and resource impacts of the disease.^1^ For example, work in the U.S. shows that older adults have less anxiety-based disorders and suicidal ideations due to COVID (Czeisler et al., 2020). Similar results were found in a study in Spain that revealed older persons had less anxiety (González-Sanguino et al., 2020). Another sample in the U.S. and Canada found that older adults had less stress and more positive affect (Klaiber et al., 2021). A longitudinal study in the Netherlands found that older adults showed little mental health change after the start of the pandemic (van Tilburg et al., 2021).^2^

However, this work generally occurs within individual locales and does not allow for large-scale tests that parse unique country-level variance from variance shared across cultures. This is important because there are many reasons to expect that such effects will be in part culture-bound. For example, models focusing on structural inequalities (Boin et al., 2016; Connor et al., 2020; Politi et al., 2021) would suggest that negative effects of a pandemic on vulnerable groups – such as adverse financial impacts, adverse resource impacts, and adverse psychological impacts – would be greatest in cultures where pre-existing inequalities were more evident. These perspectives would argue that groups that tend to have more wealth and resources (e.g., older persons and men) would be less affected by the pandemic – but the degree that this is so would be constrained by the economic and resource gap between groups. The need for research in this area is especially evident if one considers the nations from the studies above, which are overwhelmingly rich and Western.

Further, differences between young/old and men/women may be exacerbated in locales with a more general history of socioecological stressors. For example, research shows that ecological stress (such as pre-COVID pathogen levels) is associated in world-wide samples with less literacy (Conway et al., 2022), less happiness (Conway et al., 2021a), and less societal confidence (Conway et al., 2021a). Thus, there is reason to suspect that ecological stressors that existed pre-COVID may have led to exacerbating differences between groups with different levels of resources.

In the present study, we use data from around the world to evaluate the degree that the effects felt by younger persons and women are in fact common across cultures versus unique to each culture, and further test the degree that any culture-level differences in these effects are related to pre-existing structural differences (such as inequality indexes) and pre-existing stressors (such as a history of pathogen stress and extreme climates).

Because both inequality and socioecological stress exist in some degree in every nation, both models would expect a general main effect of age and biological sex on negative COVID outcomes such as levels of self-reported depression due to COVID. Thus, we hypothesize: (1) after controlling for nation-level nesting and unique effects of each culture, there will be a main effect of age and biological sex on outcome measurements related to the impacts of COVID. However, because both inequality and socioecological stress models hypothesize differences across cultures, we further hypothesize that (2) a significant amount of variance in these relationships will be due to effects unique to each culture. Finally, we hypothesize that (3) culture-level variance in these effects will be related to culture-level variance in inequality and socioecological stress. For the other variables studied here, we make no specific hypotheses – rather, we explore the amount of variance attributable to culture-general versus culture-specific effects.

### Perceived Anxiety-Ideology Relationship (PAIR) model

We further aimed to expand existing research on the influence of ideological beliefs in the psychology of pandemics. Given that pandemics are unpredictable occurrences with uncertain and often transient time courses, it is hardly surprising that there is a dearth of theory on the cross-cultural interface of psychology and perceived pandemic threat. To fill in this gap, Conway et al. (2021b) used an empirical approach to develop the Perceived Anxiety-Ideology Relationship (PAIR) model – a model which focuses on political beliefs.

The PAIR model contains two primary aspects. First, the model suggests that the *ideological match* between group-level ideologies and the outcomes of the pandemic will be crucial in determining public responses to a given pandemic. This part of the theory is culture-specific and thus provides a larger theoretical umbrella for situating cultural differences and similarities. Consider the domain of perceived threat. The PAIR model suggests that ideological groups who feel a *threatening* pandemic will benefit their own ideological ends in a given culture will be more likely to view it as a genuinely threatening; ideological groups who feel a *threatening* pandemic will *hurt* their own ideological ends will be *less* likely to view it as a threat.

Consider an example. Imagine that an ideological group wants more governmental control. Now imagine that same group perceives that increased threat from a pandemic will justify more government control. Thus, in that instance, the group’s ends are served by a pandemic perceived as maximally threatening – the more threatening the pandemic is perceived, the more psychologically justifiable their desired ends are. In this example, there is a match between a particular interpretation of the pandemic (it is threatening) and a desired governmental outcome (more governmental control). In that instance, the PAIR model suggests that the ideological group will be motivated to view the pandemic as more threatening. The PAIR model thus predicts that, rather than the actual threat level of a disease impacting governmental policy, people’s desired governmental policy will impact their perceived threat level.

Initial evidence to support the model in one cultural context (the United States) revealed that, because political beliefs interfaced in that context with disease threat, political beliefs (and not actual impacts of the disease, nor differential exposure to/trust in partisan political messaging) drove perceptions of COVID-19 (Conway et al., 2021b). This work was a useful starting point, and yet to date no research has tested culture-specific predictions of the PAIR model in Asia or other contexts. Indeed, researchers have hypothesized that the relationship between political beliefs (driven by ideology) and the perceived threat of the disease will be smaller in other parts of the world compared to the U.S., given that the U.S. currently has a higher (on average) ideological match between political beliefs/goals and COVID threat (Conway et al., 2021b). While there are multiple potential reasons for this, one possibility is that the U.S. is especially polarized currently around issues related to COVID, with conservative groups showing increasing desires to reduce government influence and liberal groups showing increasing desires to increase (liberal) government influence. The present data allow the first test of the cultural hypothesis that political beliefs will affect COVID threat perceptions more in the U.S. than in other contexts.

Second, the PAIR model suggests that the effect of ideological match on how people view a pandemic will become less pronounced as the direct experiential impact of the pandemic grows. Once people begin to be personally impacted by a disease outbreak in tangible ways (e.g., they or loved ones contract the disease, they begin to lose resources on account of pandemic), then pre-existing ideological beliefs likely play less of a role in accounting for perceptions of the disease itself. Conway et al. (2021b) found evidence of this attenuating effect of experience/impact on the ideological beliefs–perceived threat relationship in the U.S., though it was hypothesized that this effect would apply beyond the borders of the U.S. as well. The present study provides for the first cross-cultural test of this hypothesis.

Specifically, in the present study, we test the following hypotheses: (1) There will be a general tendency across all studied nations for political beliefs to predict perceived threat. (2) This tendency will be constrained by culture, such that there will be differences among nations in the political beliefs → perceived threat relationship. (2a) In particular, we expect the relationship to be larger in the U.S. compared to other cultures. (3) We predict a culture-general moderating effect of experiences/impacts of COVID on the political beliefs → perceived threat relationship, such that political beliefs will become less important to perceived threat as experiences/impacts increase.

## Materials and methods

This investigation represents 14,133 (63% female, mean age = 33.5, SD = 12.9) participants across six continents, with data from 19^3^ nations and scale translations into nine languages.

### Participants

Participants from countries around the world completed measurements of biological sex, age, and COVID-related beliefs from 20 April 2020 to 21 September 2020. Data collection occurred in the context of multiple parent projects, each of which had a different theoretical focus ranging from health to stigma to autobiographical memory. As a result, while all samples had age and biological sex measurements, not all samples completed all of the remaining scales (instead sometimes only completing a subset of those scales). This convenience sample approach allowed us to perform tests on the scales on a large sample across the world. A summary of each sample included in the present study is presented in Table 1; longer descriptions of each study context can be found in Supplementary material.

**TABLE 1:** Sample characteristics.

Nation	Characteristic			
	*N*	Age	Female (%)	Context/form/language
United Kingdom	2,198	33.9	45	
Sample 1	1,797	36.7	44	Toughness and wellbeing/long
Sample 2	301	18.2	–	Attribution/long
Sample 3	79	31.1	69	Toughness and wellbeing/long
Sample 4	21	32.3	48	Toughness and wellbeing/long
Greece	103	33.4	77	Toughness and wellbeing/long
Germany	30	42.1	76	Loneliness/short
Austria	66	30.3	71	Loneliness/short/German
Italy	872	28.3	70	
Sample 1	139	36.3	57	Loneliness/short/Italian
Sample 2	332	30.2	76	Stigma/short/Italian
Sample 3	401	23.9	46	Emerging adults/short/Italian
United States	1545	33.9	49	
Sample 1	265	38.5	49	Toughness and wellbeing/long
Sample 2	218	42.7	46	Alaskan commercial fishing/short
Sample 3	154	42.4	37	Mobile health/short
Sample 4	359	41.3	50	Psychological impacts/long
Sample 5	293	46.4	43	Ideologies and health/short
Sample 6	319	24.3	35	Emerging adults/short
Sample 7	300	39.4	81	Wellbeing of Ph.D. faculty and students/short
Poland	720	37.0	48	
Sample 1	442	34.8	50	Mood expectancies/long/Polish
Sample 2	278	41.1	44	Ideologies and health/short
Turkey	2175	30.1	48	
Sample 1	296	30.1	42	Autobiographical memory/short
Sample 2	1,879	–	50	Health/short/Turkish
Mexico	4,398	37.0	45	
Sample 1	4,127	37.1	45	Short
Sample 2	271	22.3	45	Mental health/short/Spanish
India	62	31.7	44	Toughness and wellbeing/long
Brazil	23	27.0	34	Toughness and wellbeing/long
Guinea	278	28.7	48	Insomnia/short/French
Slovenia	358	21.1	31	Emerging adults/short/Slovenian
Portugal	298	23.8	44	Emerging adults/short/Portuguese
Canada	23	39.4	81	Wellbeing of Ph.D. faculty and students/short
Lithuania	368	22.8	39	Emerging adults/short/Lithuanian
China	314	24.4	49	Emerging adults/short/Chinese
Australia	56	39.4	81	Wellbeing of Ph.D. faculty and students/short
Philippines	261	19.7	76	Filipinos’ perceptions and experiences related to COVID-19/long

Unless otherwise specified, the language for each scale was English. Italy Sample 1, Germany, and Austria were drawn from Massaccesi et al. (2021).

### Scale construction and validation

We developed a questionnaire set pertaining to key aspects of the social psychology of a pandemic (see, e.g., Van Bavel et al., 2020): (1) *Perceived Threat*, (2) *Negative Impacts*, (3) *Experiences*, and (4) *Government Response.* This questionnaire set was initially psychometrically validated in the United States. Then, in the present study, we validated the psychometric properties of the scales across all the nations studied. As can be seen in Supplementary material, those analyses reveal that the scales have good psychometric properties, both across international contexts and within each nation studied here.

#### Perceived coronavirus threat questionnaire

All measurements used a rating scale anchored by “not true of me at all” and “very true of me.” The Perceived Threat Questionnaire contained 6 items concerning how threatened or worried they were about COVID-19, for example, “Thinking about the coronavirus (COVID-19) makes me feel threatened.” The short version of the scale contained three of these items (see the Supplementary material).

#### Coronavirus impacts questionnaire

Participants completed 9 items concerning their perceived impacts from COVID-19, including how they had been financially impacted [“I have lost job-related income due to the Coronavirus (COVID-19)”], how they had been impacted in terms of resources, and how they had been psychologically impacted [“The Coronavirus (COVID-19) outbreak has impacted my psychological health negatively”].

#### Coronavirus experiences questionnaire

Participants completed 10 items concerning their experiences with COVID-19. The questions stemmed from several conceptual dimensions: Whether participants might have had COVID-19 or other related diseases recently [“I have been diagnosed with coronavirus (COVID-19)”], whether they might have known others who had COVID-19 (“I know someone who has had coronavirus-like symptoms in the last two months”), and how much COVID-19 news they had been consuming [“I watch a lot of news about the Coronavirus (COVID-19)”].

#### Political beliefs: Governmental response to coronavirus questionnaire

The Governmental Response Scale involved 12 items across six dimensions (2 items per dimensions) concerning what they believed about their government’s response to the crisis. For each dimension, participants completed two questions. All the questions and scales (many of which were adapted from prior work; Conway et al., 2017b; Conway and Repke, 2019) can be found in Supplementary material.

*Restriction* questions measured the degree to which participants wanted their governments to restrict citizens’ behavior to help stop the spread of the virus. *Punishment* questions measured the degree to which participants wanted their governments to punish citizens who violated social distancing rules. *Reactance* questions measured the degree to which participants felt angry that their governments were taking away their freedom during the crisis. *Research* questions measured the degree to which participants wanted their governments to fund research on the virus. *Stimulus* questions measured the degree to which participants wanted their governments to give money back to individuals to help the economy. *Informational Contamination* questions measured the degree to which participants felt that they could not trust their governments to provide accurate information during the crisis.

### Age and biological sex

In all samples, participants completed measurements of their age (in years) and their biological sex assigned at birth.

### Culture-level variables

To better understand cultural variability in the effects of age and biological sex across cultures, we further included variables that prior research would suggest might help explain such variance. These variables fell into two categories: (1) Some of these variables are related to socioecological stressors (see Conway et al., 2017a, 2019): Historic (pre-COVID) nation-level *pathogen prevalence* (Fincher and Thornhill, 2012), two nation-level measurements of *Climate Stress* (hot and cold stress; Van de Vliert, 2013), and GDP per capita (conceptually inversely related to socioecological stress; Conway et al., 2017a). (2) Some of these variables are related to structural inequality or societal hierarchies. These included the Freedom House *Totalitarianism Index* (Conway et al., 2017a), Hofstede’s *Collectivism Index* (Hofstede, 2001), and three measurements of structural inequality: The GINI Coefficient (World Bank Research Development Group, 2020), the Gender Inequality Index (United Nations, 2020), and the Discrimination Index (Van de Vliert, 2019). These three inequality indices were all highly correlated (*r*’s ranging from 0.79 to 0.89) and thus were standardized and combined into a single *Inequality Index* (standardized *alpha* = 0.94).

### Analytic strategy

#### Linear mixed models

Our primary strategy was to use Linear Mixed Models to evaluate the degree that key relationships were significantly captured by shared across-culture variance, unique within-culture variance, or both. To accomplish this, we ran linear mixed models in *R* using the lme4 package (see Winter, 2013); to estimate probability values, we used the popular lmerTest supplement (Kuznetsova et al., 2017). Specifically, we first ran models for each relationship that did not include an interaction term, but which did directly account for the nesting of the data within each nation. Then we ran our key models that also included the nation-level interaction term for each effect. This allows us to test, using linear mixed models that account for the nested nature of the data, the degree that a given effect is significant across cultures (represented by Column 2 in Table 7) versus the results of unique within-culture effects (represented by Column 3 in Table 7). For example, when considering the relationship between age and psychological impacts, this method allows us to test the degree (while accounting for the nested nature of the data) that the relationship is common across cultures versus whether or not the relationship is culturally constrained – or whether both are statistically significant and thus each have independent contributions.

**TABLE 2A:** The relationship of age with perceived COVID-19 threat and impacts across nations.

Nation	Measure			
	Threat	Financial	Resource	Psychology
United Kingdom (*n* = 2,204)	–0.01	−0.05*	–0.02	−0.07***
Greece (*n* = 104)	–0.04	–0.00	−0.20*	−0.25*
Germany (*n* = 32)	0.12	–0.27	–0.12	–0.17
Austria (*n* = 67)	0.15	−0.39***	0.24*	−0.25*
Italy (*n* = 473–851)	0.09^∧^	–0.01	–0.03	–0.04
United States (*n* = 917–1,488)	–0.04	−0.16***	−0.14***	−0.23***
Poland (*n* = 720)	0.14***	0.06	−0.12**	−0.08*
Turkey (*n* = 302)	0.01	−	−	−
Mexico (*n* = 4,398)	−0.06***	−0.03*	−0.11***	−0.19***
Guinea (*n* = 239)	−	−0.14*	−0.15*	−
India (*n* = 62)	–0.04	–0.13	–0.10	–0.03
Brazil (*n* = 23)	0.05	–0.01	–0.05	–0.18
Slovenia (*n* = 264)	−	–0.03	–0.06	−0.17**
Portugal (*n* = 251)	−	0.06	–0.03	−0.12^∧^
Lithuania (*n* = 270)	−	−0.11^∧^	–0.01	–0.07
China (*n* = 197)	−	0.08	−0.23***	−0.12^∧^
Australia (*n* = 51)	–0.11	−	−	−
Canada (*n* = 21)	0.38^∧^	–0.01	–0.06	–0.09
Philippines (*n* = 261)	–0.08	−0.12^∧^	–0.00	–0.08
TOTAL	–0.02	−0.04***	−0.08***	−0.13***

^∧^*p* ≤ 0.10; *p ≤ 0.05; **p ≤ 0.01; ***p ≤ 0.001.

**TABLE 2B:** The relationship of age with government response across nations.

Nation	Measure					
	Restriction	Punishment	Reactance	Research	Stimulus	Contamination
United Kingdom (*n* = 2,204)	–0.01	−0.06***	0.02	0.01	0.02	0.05*
Greece (*n* = 104)	–0.01	–0.05	–0.02	–0.12	0.09	–0.03
United States (*n* = 918)	0.02	−0.15***	−0.11**	0.00	0.01	0.02
Poland (*n* = 718)	0.05	0.08*	0.16***	0.09*	0.04	0.10**
Guinea (*n* = 238)	0.09	0.09	−	0.06	–0.10	−
India (*n* = 62)	0.26*	0.20	–0.12	0.07	0.13	–0.15
Brazil (*n* = 23)	–0.06	0.12	0.04	–0.10	–0.07	0.31
Philippines (*n* = 261)	−0.20***	–0.03	0.09	0.02	0.03	–0.08
TOTAL	0.01	−0.04***	0.01	0.02	0.02	0.04**

*p ≤ 0.05; **p ≤ 0.01; ***p ≤ 0.001.

**TABLE 3:** The relationship of age with COVID experiences across nations.

Nation	Measure		
	Personal	Other	News
United Kingdom (*n* = 2,204)	−0.05**	−0.04^∧^	0.05*
Greece (*n* = 104)	−0.26**	–0.02	−0.21*
Italy (*n* = 472–870)	0.06	0.04	0.03
United States (*n* = 917–1,198)	−0.19***	−0.20**	0.03
Poland (*n* = 718)	−0.08*	–0.02	0.21***
Mexico (*n* = 4,398)	−0.07***	−0.09***	−0.07**
India (*n* = 62)	−0.35**	−0.33**	0.20
Brazil (*n* = 23)	–0.18	0.14	0.22
Slovenia (*n* = 259)	0.05	0.01	–0.03
Portugal (*n* = 251)	–0.02	–0.02	−0.14*
Lithuania (*n* = 270)	–0.05	–0.08	−0.20***
China (*n* = 197)	−0.22**	−0.18*	0.04
Philippines (*n* = 261)	0.05	0.02	−0.18**
TOTAL	−0.07***	−0.07***	–0.00

^∧^*p* ≤ 0.10; *p ≤ 0.05; **p ≤ 0.01; ***p ≤ 0.001.

**TABLE 4:** The relationship of biological sex with perceived COVID threat and impacts across nations.

Nation	Measure			
	Threat	Financial	Resource	Psychology
United Kingdom (*n* = 2,204)	0.00	0.07**	0.09***	0.06*
Greece (*n* = 104)	–0.02	0.05	–0.01	0.07
Germany (*n* = 32)	–0.21	–0.25	0.01	–0.28
Austria (*n* = 67)	–0.06	–0.10	–0.05	–0.10
Italy (*n* = 473–851)	−0.19***	−0.10**	0.01	−0.19***
United States (*n* = 917–1,488)	−0.12***	–0.01	–0.02	−0.11***
Poland (*n* = 720)	–0.03	−0.08*	0.03	−0.10**
Turkey (*n* = 1,885)	−0.16***	−0.04^∧^	−0.16***	−0.27***
Mexico (*n* = 4,399)	−0.12***	0.04*	0.04**	−0.11***
Guinea (*n* = 239)	−	0.02	–0.01	−
India (*n* = 62)	–0.18	–0.12	0.06	–0.07
Brazil (*n* = 23)	0.28	0.18	–0.16	0.05
Slovenia (*n* = 264)	−	–0.10	–0.00	−0.12*
Portugal (*n* = 251)	−	–0.02	–0.00	−0.13*
Lithuania (*n* = 270)	−	–0.09	–0.04	−0.15*
China (*n* = 197)	−	–0.01	–0.06	–0.09
Australia (*n* = 51)	–0.18	−	−	−
Canada (*n* = 23)	–0.04	–0.01	–0.06	–0.09
Philippines (*n* = 261)	–0.09	–0.00	–0.03	–0.05
TOTAL	−0.10***	0.00	0.00	−0.11***

^∧^*p* ≤ 0.10; *p ≤ 0.05; **p ≤ 0.01; ***p ≤ 0.001. Biological sex is dummy-coded male = 2, female = 1; positive correlations mean men are higher and negative correlations mean women are higher.

**TABLE 5:** The relationship of biological sex with government response across nations.

Nation	Measure					
	Restriction	Punish	Reactance	Research	Stimulus	Contamination
United King. (*n* = 2,204)	−0.07***	0.11***	0.19***	0.03	–0.01	0.09***
Greece (*n* = 104)	–0.09	0.18^∧^	0.06	–0.04	0.03	–0.03
United States (*n* = 918)	−0.12***	0.05	0.17***	−0.09**	−0.15***	0.05
Poland (*n* = 718)	−0.08*	−0.09*	–0.01	0.05	–0.05	−0.12***
Turkey (*n* = 1,885)	−0.06**	−0.06*	0.06**	−0.14***	−0.06**	–0.04
Guinea (*n* = 238)	0.04	–0.08	−	–0.01	–0.02	−
India (*n* = 62)	–0.17	–0.21	0.04	–0.03	–0.07	–0.05
Brazil (*n* = 23)	–0.10	0.20	−0.36^∧^	–0.16	–0.18	–0.06
Philippines (*n* = 261)	–0.04	–0.10	0.01	0.16*	0.08	0.10
TOTAL	−0.07***	0.01	0.11***	−0.04**	−0.05***	0.01

^∧^*p* ≤ 0.10; *p ≤ 0.05; **p ≤ 0.01; ***p ≤ 0.001.

**TABLE 6:** The relationship of biological sex with COVID experiences across nations.

Nation	Measure		
	Personal	Other	News
United Kingdom (*n* = 2,204)	0.14***	0.14***	0.13***
Greece (*n* = 104)	0.19^∧^	–0.05	0.03
Italy (*n* = 472–870)	–0.03	0.01	0.00
United States (*n* = 917–1,198)	–0.01	–0.02	0.07*
Poland (*n* = 718)	0.08*	0.11**	−0.09***
Turkey (*n* = 1,885)	–0.01	0.04^∧^	0.07*
Mexico (*n* = 4,399)	−0.03^∧^	–0.01	0.07***
India (*n* = 62)	–0.07	–0.00	–0.13
Brazil (*n* = 23)	0.00	–0.00	0.08
Slovenia (*n* = 259)	–0.08	–0.05	0.04
Portugal (*n* = 251)	0.01	–0.00	–0.02
Lithuania (*n* = 270)	0.04	0.09	–0.03
China (*n* = 197)	0.02	0.04	0.02
Philippines (*n* = 261)	0.06	–0.02	0.08
TOTAL	0.02***	0.03***	0.05***

^∧^*p* ≤ 0.10; *p ≤ 0.05; **p ≤ 0.01; ***p ≤ 0.001.

**TABLE 7:** Linear mixed models: comparing across-culture versus within-culture effects for age and biological sex.

	Across-culture (zero order main effect)	Across-culture (main effect)	Within-culture (age × nation interaction)
**Age**			
Threat	2.15	0.01	3.03***
**Impacts**			
Financial impacts	19.31***	7.04**	3.00***
Resource impacts	63.32***	5.66*	2.54***
Psychological impacts	193.51***	20.93***	3.30***
**Experiences**			
Personal	44.73***	5.83*	3.79***
Other	45.58***	3.09	3.44***
News	0.02	4.29*	7.38***
**Government response**			
Restriction	0.22	1.99	2.70**
Punishment	8.40**	0.24	4.08***
Reactance	0.62	0.66	5.22***
Research	1.43	0.03	0.97
Stimulus	1.36	0.32	0.77
Informational contamination	5.84*	0.19	1.43
**Biological sex**			
Threat	111.59***	2.88	3.34***
**Impacts**			
Financial impacts	0.10	0.46	2.26**
Resource impacts	0.32	0.26	4.30***
Psychological impacts	129.31***	11.30***	7.92***
**Experiences**			
Personal	4.62*	0.66	3.57***
Other	14.11***	0.42	2.83***
News	20.41***	1.14	4.50***
**Government response**			
Restriction	31.97***	4.37*	0.78
Punishment	0.10	0.02	5.09***
Reactance	62.19***	0.00	4.43***
Research	9.95**	0.59	5.62***
Stimulus	12.83***	1.79	1.99*
Informational contamination	0.80	0.05	4.40***

Numbers are *F*-values from linear mixed model tests. *p ≤ 0.05; **p ≤ 0.01; ***p ≤ 0.001.

#### Within-nation correlations

Further, for descriptive purposes, we produced tables of correlations within-country. To create summary scores for the entire sample, we standardized all measures within each dataset. For nation-level summations, we further standardized data within-nation. As a result, final weighted averages capture the average within-country effects across the world while controlling directly for across-nation differences.^4^

#### Across-nation correlations

To illuminate the degree that nation-level variables might help us understand cultural variability, we correlated the nation-level variables (e.g., Inequality Index) with the strength of relationships (e.g., the strength of the relationship between biological sex and psychological impacts) across cultures. To do this, we imputed scores for each participant for the nation-level variables and relationship strength. As a result, these correlations represent effect measurements that are weighted by participant sample size in each nation. This method has pros and cons: it provides an estimate that does not over-rely on nations with small samples, but it also means the results reflect more on the large-sample nations as well. Thus, while caution is warranted in interpretation, these weighted correlations are at a minimum valuable at an exploratory level.

#### Summary variables: Perceived Anxiety-Ideology Relationship model

For summary tests of the PAIR model, we created summary variables in a fashion identical to those created in Conway et al. (2021b). Specifically, we averaged all experiences and impacts scales into a single *Experiences/Impacts*^5^ summary scale (representing increasing experiences with and impacts of COVID), averaged all Government Response items (except informational contamination) into a single *Political Beliefs* scale (representing a desire for more government intervention across categories), and used reversed-scored informational contamination as the *Messaging Trust* scale (representing the degree that participants trusted their government to provide accurate information about COVID).

## Results

### To what degree are the effects of age and biological sex on COVID psychology influenced by cultural uniqueness?

Age and biological sex results by nation are presented in Tables 2–6. As in prior research (e.g., Ausín et al., 2020; Vahia et al., 2020), there was a tendency across national contexts for both older participants and men to have fewer negative impacts and experiences associated with the COVID-19 pandemic.^6,7^ Specifically, older participants showed significantly less financial, psychological, and resource impacts from COVID, while men showed significantly less perceived threat and psychological impacts from COVID.

Are these age and biological sex effects better captured by considering across-culture similarity or each culture’s uniqueness? Our linear mixed models provide a clear overall answer to that question. Comparing the Across-Culture Effects (Column 2 of Table 7) to the Within-Culture Effects (Column 3 of Table 7) reveals that, while the majority of culture interaction effects are significant, only a small number of across-culture main effects remain significant when accounting for the unique impacts of each culture. As a result, in the main these results suggest that many of the effects often talked about in broad terms – such as the effects of age (e.g., Vahia et al., 2020) and biological sex (e.g., Ausín et al., 2020) on negative impacts of COVID – are in fact better characterized as culture-dependent.

Notably, perhaps the most consistent pan-cultural finding is that both young people and women experienced significantly more *psychological* distress as a result of COVID. While in both cases cultural variability in the relationship was also significant, this importantly does reveal that there is nonetheless quite a bit of similarity in those effects across cultures.

### Exploring nation-level factors that might explain culture-level variance in age and biological sex effects

Tables 8, 9 show the weighted correlations between nation-level effects and the nation-level inequality/stressor variables.^8^ We note that although most of these correlations are significant, they should nonetheless be interpreted with inferential caution because of their imputed nature.

**TABLE 8:** Explaining culture-level variance: weighted correlations between culture-level socioecological variables and the relationships of age and biological sex with outcomes.

	Pathogens	Cold stress	Heat stress	GDP/PC
**Age**				
Threat	–0.55	0.19	0.33	0.20
**Impacts**				
Financial impacts	0.08	–0.29	–0.25	–0.42
Resource impacts	–0.56	0.23	–0.11	0.27
Psychological impacts	–0.43	–0.06	0.18	0.08
**Experiences**				
Personal	–0.16	–0.22	–0.19	–0.21
Other	–0.33	–0.28	–0.05	–0.13
News	–0.50	0.12	0.26	0.49
**Biological sex**				
Threat	–0.50	–0.09	–0.16	0.40
**Impacts**				
Financial impacts	0.21	–0.25	–0.55	0.03^∧^
Resource impacts	–0.14	–0.48	–0.41	0.34
Psychological impacts	–0.38	–0.27	0.22	0.49
**Experiences**				
Personal	–0.68	0.11	–0.04	0.46
Other	–0.67	0.31	–0.19	0.32
News	–0.05	0.23	–0.41	0.20

All correlations weighted by sample size. All correlations significant at *p* ≤ 0.001 unless otherwise noted. For Age, higher scores mean that cultures high in the variable in each column have a positive relationship between age and the variable in each row; lower scores mean that cultures high in the variable in each column have a negative relationship between age and the variable in each row. For example, the negative relationship between pathogens and threat means that cultures high in pathogens are more likely to have younger people perceive COVID as threatening than older people. For biological sex, higher scores mean that cultures high in the variable in each column have a positive relationship between biological sex and the variable in each row; lower scores mean that cultures high in the variable in each column have a negative relationship between biological sex and the variable in each row. For example, the negative relationship between pathogens and threat means that cultures high in pathogens are more likely to have women perceive COVID as threatening than men. ^∧^*p* < 0.01.

**TABLE 9:** Explaining culture-level variance: weighted correlations between culture-level socioecological variables and the relationships of age and biological sex with outcomes.

	Inequality	Totalitarianism	Collectivism
**Age**			
Threat	–0.60	–0.28	–0.30
**Impacts**			
Financial impacts	−0.00^∧∧^	0.33	0.40
Resource impacts	–0.49	–0.49	–0.37
Psychological impacts	–0.58	–0.13	–0.44
**Experiences**			
Personal	–0.15	–0.08	0.11
Other	–0.35	–0.11	0.02
News	–0.60	–0.47	–0.63
**Biological sex**			
Threat	–0.61	–0.43	–0.49
**Impacts**			
Financial impacts	0.19	0.01^∧∧^	–0.06
Resource impacts	–0.28	–0.37	–0.31
Psychological impacts	–0.47	–0.47	–0.51
**Experiences**			
Personal	–0.70	–0.43	–0.63
Other	–0.71	–0.33	–0.58
News	0.06	–0.21	–0.23

All correlations weighted by sample size. All correlations significant at *p* ≤ 0.001 unless otherwise noted. For Age, higher scores mean that cultures high in the variable in each column have a positive relationship between age and the variable in each row; lower scores mean that cultures high in the variable in each column have a negative relationship between age and the variable in each row. For example, the negative relationship between inequality and threat means that cultures high in inequality are more likely to have younger people perceive COVID as threatening than older people. For biological sex, higher scores mean that cultures high in the variable in each column have a positive relationship between biological sex and the variable in each row; lower scores mean that cultures high in the variable in each column have a negative relationship between biological sex and the variable in each row. For example, the negative relationship between inequality and threat means that cultures high in inequality are more likely to have women perceive COVID as threatening than men. ^∧∧^*p* > 0.05.

Two findings stand out in these analyses. First, for both age and biological sex, Inequality (and to a lesser degree, Totalitarianism and Collectivism) tends to be predictive of relationships for threat and psychological impacts. Consistent with models based on systemic inequalities (Boin et al., 2016; Connor et al., 2020), younger persons and women showed more negative effects of COVID if they lived in societies with more pre-existing inequalities.

Second, while generally we found that pre-existing ecological stressors similarly led to more negative outcomes for younger persons and women, that was especially so (and most consistently so) for pre-existing pathogen prevalence. Since pathogen prevalence is known to effect other variables related to inequality (see, e.g., Conway et al., 2022), this result might dovetail with results from pure inequality measurements.

### Tests of the perceived anxiety-ideology relationship model

To test the PAIR model, we first replicated Conway et al.’s (2021b) U.S. findings using our U.S. sample specifically. Consistent with that prior study, Political Beliefs (*beta* = 0.52, *p* < 0.001) mattered more for predicting Perceived Threat than Experiences/Impacts (*beta* = 0.22, *p* < 0.001) or Messaging Trust (*beta* = −0.03, *p* > 0.30). This provides a conceptual replication of Conway et al. (2021b) on an entirely new set of U.S. participants across multiple data collection mechanisms and research contexts in that country.

Aggregating data from all other nations (U.S. participants excluded), we performed the same simultaneous regression tests. Consistent with the tenets of the PAIR model (Conway et al., 2021b), the relative weight of political beliefs was weaker in other parts of the world, with Political Beliefs [*beta* (5,247) = 0.39, *p* < 0.001] and Experiences/Impacts [*beta* (5,247) = 0.28, *p* < 0.001] having effects closer together in strength compared to the U.S. Similar to the U.S., effects of Messaging Trust on Perceived COVID-19 Threat were generally small internationally [*beta* (5247) = 0.02, *p* > 0.12]. However, as illustrated by Table 10, there was a great deal of variability across nations.

**TABLE 10:** Relative predictive validity of impacts/experiences, political beliefs, and trust in political messaging on perceived COVID threat across nations.

Nation	Measure		
	Impacts	Beliefs	Messaging
United Kingdom (*n* = 2,204)	0.39***	0.35***	0.07***
Greece (*n* = 104)	0.30**	0.31**	0.12
Philippines (*n* = 261)	0.26***	0.18**	−0.17**
United States (*n* = 560)	0.22***	0.52***	–0.04
Poland (*n* = 720)	0.24***	0.44***	0.05^∧^
Turkey (*n* = 1,885)	0.21***	0.40***	0.01
India (*n* = 62)	0.28*	0.21^∧^	−0.40**
Brazil (*n* = 23)	0.32	0.43^∧^	0.12

^∧^*p* ≤ 0.10; *p ≤ 0.05; **p ≤ 0.01; ***p ≤ 0.001. All tests were regressions where all three predictor variables were entered in simultaneously.

This variability was statistically confirmed with linear mixed models. As can be seen in Table 11, although there were large and statistically significant pan-cultural effects across all three variables, there were also significant effects attributable to unique differences within culture as well. Although the U.S. showed the largest discrepancy between Political Beliefs and Experiences/Impacts on predicted Perceived COVID-19 Threat, both Poland and Turkey showed a similar pattern to the U.S. (with fairly large discrepancies between Beliefs and Experiences/Impacts), whereas the Philippines, Greece and the UK all showed a pattern divergent from the U.S. (with similar effect sizes for both Beliefs and Experiences/Impacts). Thus, while it is clear that political beliefs matter to perceived threat in all parts of the world we studied, it is also clear that the relative weight of those beliefs varies from nation-to-nation.

**TABLE 11:** Linear mixed models: comparing across-culture versus within-culture effects for PAIR predictions of perceived threat.

	Across-culture (zero order main effect)	Across-culture (main effect)	Within-culture (sex × nation interaction)
Experiences/impacts	2,714.10***	189.17***	18.78***
Political beliefs	1,994.80***	246.50***	7.10**
Messaging	177.81***	29.90***	10.80***

*p ≤ 0.05; **p ≤ 0.01; ***p ≤ 0.001.

Finally, we tested the second prediction from the PAIR model – that political variables become less important as experiences and impacts become greater. Consistent with the model, looking at data from all nations simultaneously, the effect of Political Beliefs was moderated by Experiences/Impacts [interaction *beta* (5,929) = −0.04, *p* < 0.001; LCI = −0.06, UCI = −0.02]. Descriptive analyses revealed the expected effect. The effect of Political Beliefs on Perceived COVID-19 Threat was highest for participants who had been less impacted by the disease (effect in the lower third = 0.43, LCI = 0.40, UCI = 0.46) than for those who had been more impacted by the disease (effect in the upper third = 0.35, LCI = 0.32, UCI = 0.39).^9^ This is consistent with the PAIR model’s prediction that as experiences with (and impacts of) COVID-19 are higher, political beliefs play less of a role in perceptions of threat.

However, as can be seen in Table 12, great variability emerged for this prediction across national contexts. Indeed, the effect appears largely driven by the UK, which showed the largest effect in the predicted direction. The U.S. showed an effect roughly the same magnitude as in past work (Conway et al., 2021b), and the Philippines and India similarly showed effects in the same direction (though, like the U.S., non-significant at the nation-level). However, Poland and Turkey essentially showed zero effect and Greece showed a nearly significant effect in the opposite direction, such that increasing experiences and impacts led to more effect of political variables. Thus, these results suggest that cultural factors moderate the experiences/impacts on the relationship between political variables and perceived threat.

**TABLE 12:** Moderating impact of experiences/impacts on the relationship between political beliefs and perceived threat.

Nation	Measure		
	Moderation (interaction)	Effect at low impact	Effect at high impact
United Kingdom (*n* = 2,204)	−0.09***	0.41***	0.23***
Greece (*n* = 104)	0.12^∧^	0.22*	0.48***
Philippines (*n* = 261)	–0.05	0.31**	0.12
United States (*n* = 560)	–0.03	0.47***	0.40***
Poland (*n* = 720)	0.00	0.44***	0.45***
Turkey (*n* = 1,885)	0.01	0.40***	0.41***
India (*n* = 62)	–0.03	0.37**	0.30
Brazil (*n* = 23)	0.04	0.37	0.45
TOTAL CUMULATIVE	−0.04***	0.43***	0.35***

^∧^*p* ≤ 0.10; *p ≤ 0.05; **p ≤ 0.01; ***p ≤ 0.001. Moderation, moderating effect of impacts/experiences on the relationship between political beliefs and perceived threat. Effect at low impact, effect of political beliefs on perceived threat for bottom 1/3 of persons on impacts/experiences measure. Effect at high impact, effect of political beliefs on perceived threat for top 1/3 of persons on impacts/experiences measure.

## Discussion

Understanding how cultural psychology interfaces with the pandemic is an important topic (Khazaie and Khan, 2020; Albarracin and Jung, 2021; Bond, 2021; Jetten et al., 2021; Kashima, 2021; Liu, 2021). To aid in this endeavor, drawing from culturally flexible psychological theories, the present results identified the influence of culturally unique factors in better understanding the psychology of COVID-19. Specifically, our results reveal that (1) although both similarities and differences in the effects of age and biological sex exist across cultures, on average far more significant effects occur because of culture-specific effects. (2) They further suggest that, consistent with models focusing on how stressors can exacerbate inequalities (Boin et al., 2016; Connor et al., 2020), both pre-existing nation-level inequalities and pre-existing ecological stressors can cause women and young people to be disproportionately affected by COVID. (3) Finally, these results provide novel evidence both supporting the PAIR model of pandemic psychology and suggesting the importance of better understanding local cultures in applying the model.

Below, we expound on these insights and discuss limitations with our study.

### Age and biological sex

The present study revealed both across-cultural similarities and differences in the effects of age and biological sex on perceived threat, impacts, experiences, and desired government response with respect to COVID. On the one hand, consistent with prior COVID-19 pandemic research (Vahia et al., 2020), there was a tendency across national contexts for older participants to have fewer negative impacts and experiences associated with the COVID-19 pandemic, in particular showing that older participants had less psychological anxiety, less resource stress, and less financial stress. Also consistent with prior research on biological sex-based COVID-19 effects (e.g., Ausín et al., 2020; Connor et al., 2020), women perceived COVID as more threatening and reported more psychological distress as a result of COVID-19.

One of the primary advances of our multi-national dataset is the ability to directly test cultural similarities and differences in a linear mixed model design. These analyses revealed that the similarities across cultures were often overshadowed by unique differences within cultures. Descriptively, our data suggest that some of these differences may be because stressful ecological events exacerbate existing inequalities (Boin et al., 2016; Connor et al., 2020) and, indeed, a history of pre-existing stressful ecologies itself pre-disposes cultures to this pattern. However, we do not want to over-interpret these data. Rather, we suggest that our data provide important context from which cultural psychologists can begin to more fully understand such potential differences in age and biological sex effects as they pertain to COVID-19 across cultures.

### Perceived Anxiety-Ideology Relationship model: The importance of cultural ideological matching

It is difficult to build cross-culturally valid theories of phenomena such as pandemics. Not only is each culture different – and thus any measurement transmuted from one culture to another is by definition imprecise – but also pandemics ebb and flow, making capturing the psychology of them challenging. This means we need to not only build theories that are culturally flexible, but also to collect data in multiple cultural locales to test those theories.

In the present study, we provided the first across-culture examination of the PAIR model. Available data to date had been exclusively in one Western nation (Conway et al., 2021b), and thus a need for expansion into other parts of the world – including non-WEIRD contexts – was paramount. In the present study, we provided one such test. That test both confirmed some of the basic conclusions of the PAIR model and suggested a need for cultural refinement of the model.

#### Confirming the model

First, drawing on years of motivated reasoning research (e.g., Jost et al., 2003), the model suggests at a broad level the importance of people’s desired governmental response in helping us understand why people view a disease as threatening. Consistent with that general assertion, in all six nations with an *n* > 100, political beliefs relevant to the desired government response were significant predictors of perceived COVID-19 threat (and in the two other nations, the general pattern was the same), and overall, political beliefs – and not experiences/impacts or political messaging – was the strongest predictor in our data worldwide. This highlights the importance of culturally relevant ideological beliefs.

Further, the PAIR model also predicts that the degree political beliefs are related to perceived threat ought to vary based on the within-cultural ideological match between disease threat and desired ideological ends. We do not have specific measurements of “match” in the present study, but prior researchers had suggested that, due to the unique cultural conditions of the U.S., the match would likely be higher there than in other parts of the world. This was borne out in our data: Although some nations mirrored the U.S. more closely than others, the U.S. showed the strongest tendency for political beliefs to predict COVID stress among all the nations we studied.

Linear mixed models revealed both areas of commonality across cultures and further highlighted the validity of the continued emphasis of cultural psychologists on cultural variability in the social psychology of COVID (e.g., Bond, 2021; Kashima, 2021; Liu, 2021). Indeed, this is especially in evidence with respect to the PAIR model’s predictions of the political beliefs-perceived threat relationship. As noted above, the PAIR model explicitly predicts cultural variability in the degree (and direction) of the political beliefs-threat relationship, because that model asserts that the cultural match between a given political ideology and perceived threat is the driver of threat perceptions. Since that match will vary from culture to culture, as such, the model provides a direct framework for understanding cultural variability in threat perceptions by highlighting specific kinds of variables cultural researchers can identify and study. And indeed, in our work, the basic PAIR model prediction of cultural variability was borne out in linear mixed models.

#### Qualifying the model

On the other hand, in its original instantiation, the PAIR model did not directly predict cultural variability in the moderating impact of experiences and impacts on the political beliefs-ideology relationship (see Conway et al., 2021b). Indeed, because of this, we expected that across most places and most times, the presence of direct impacts from a disease would make political beliefs less important. The clear variability in this moderating effect (see Table 12) highlights again the importance of considering cultural context in making such blanket statements, and likely reflects that the originators of the PAIR model were themselves used to doing research in WEIRD contexts. In fact, it is easy to see in hindsight that the premise of the PAIR model would in fact expect some cultural variability in the moderating impact of experiences/impacts. It is possible, for example, that experiencing impacts with a disease might, under some circumstances, actually increase the relative importance of political beliefs as people look to different sources (either governmental or otherwise) to solve problems.

### Limitations

Despite its valuable contributions, this work is not without limitations. Many of these limitations pertain to a tradeoff between the necessity of producing measurements with reasonable speed and the necessity of maintaining scientific rigor during events that have unpredictable time courses. For example, the present work uses convenience samples from existing projects, each with different focal points, and thus does not have a standardized format that is identical across cultures.

Further, although we have a reasonably large sample of persons from different cultural locales globally, our sample is far from representative of the many and varied cultures in the world. Thus, we cannot say for certain that we would get the same levels of similarities and differences if we had included other cultural locales. However, no effort is perfect in this kind of endeavor; and this dataset provides a novel contribution to the literature in this regard.

Finally, like the vast majority of work in the field, our work does not directly account for the non-independence of nations (see, e.g., Claessens and Atkinson, 2022). While this is an important limitation, we believe our primary conclusions nonetheless are likely largely unaffected by this possibility. The non-independence problem is most in evidence when researchers regress one nation-level variable (e.g., pathogen prevalence) on another nation-level variable (e.g., individualism). Indeed, when Claessens and Atkinson (2022) re-analyzed six cultural psychology studies to account for non-independence, all six primary cultural psychology examples involved such basic nation-level associations. However, our present linear mixed models generally do not run the same level of risk associated with non-independence of nations. For example, our cultural uniqueness approach does not primarily correlate one nation-level variable with another; rather, it evaluates differences in within-culture variance on specific relationships while controlling for the nested nature of the data. While this can of course be affected by non-independence, it is not clear that it would inevitably produce false positives; in fact, the shared variance of local cultures might, in this instance, actually inhibit our ability to find effects instead of artificially producing them. One of our primary interests is in parsing unique cultural variance in *X*–*Y* relationships at the individual level, and non-independence across nations may interfere with the ability to find this kind of cultural uniqueness because it makes it harder to show that each culture is different from its surrounding cultures. Of course, some of our conclusions do involve traditional *X*–*Y* correlations at the nation-level, and we urge caution for those exploratory tests.

## Conclusion

Cultural psychologists have correctly noted the need to consider culture more carefully as we investigate the pandemic. However, often studies are conducted in isolated pockets using different measurements. The present study helps fill this gap. By using measurements validated across multiple cultural contexts, our large research team from around the world was able to separate the effects that are shared across cultures from those that are unique to specific cultures. In addition, we provided novel evidence consistent with prior theorizing about the culture-specific effects of pandemics on younger persons and women.

This work also has important implications for medical practitioners. In particular, it suggests that there is no “one size fits all” approach to successfully managing a pandemic. The prevalence of cultural uniqueness in explaining pandemic effects in the present work reveals the danger of promoting any health strategy in a cultural vacuum. In fact, our data suggest that strategies to deal with pandemics must be in part be tailored to each unique cultural context.

## Data availability statement

The raw data supporting the conclusions of this article will be made available by the authors, without undue reservation.

## Ethics statement

The studies involving human participants were reviewed and approved by the ethics boards at the University of Montana, University of Greenwich, University of Alabama at Birmingham, Instituto Nacional de Psiquiatría Ramón de la Fuente Muñiz, Swinburne University of Technology, McGill University, University of Minnesota, Università Cattolica del Sacro Cuore, Mykolas Romeris University, University of Coimbra, University of Ljubljana, Koffi Annan University, Kirikkale University, University of Amsterdam, Universidad de Sonora, and the University of Notre Dame. The patients/participants provided their written informed consent to participate in this study.

## Author contributions

LGC, SW, and AZ contributed to the conception and design of the study. AZ organized the database. LGC and SW performed statistical analyses. LGC, SW, and AZ wrote the first draft of the manuscript. All authors were involved in data collection, translation (when needed), and refinement of ideas and contributed to the manuscript revision.

## Footnotes

1 A complementary reason why women and younger persons were more psychologically affected by the pandemic is that they lost close others (e.g., spouses and parents/grandparents) at higher levels, as males and the elderly had higher death rates during the pandemic (Krams et al., 2020).

2 This work on older adults might seem puzzling at first glance because older adults were disproportionately more likely to suffer hospitalization and death from COVID (Center for Disease Control, 2022). How, then, did they report less anxiety from COVID and fewer mental health consequences? It is quite possible that the effects of age on (say) death diverge from those on (say) mental health because it is in mental health domains where the structural inequalities might be especially likely to disadvantage younger persons. Younger persons on average have less access to financial and social resources that might help offset the mental strain of the pandemic. The present study helps us better understand these relationships by parsing cultural similarities and differences in age-based effects on resource and mental health stress.

3 Because some of the individual parent studies cast a wide national net, our larger study included participant data from 48 nations; however, in many cases, there were <10 participants per nation. We thus excluded data from these nations from all key cultural tests reported in the text. These data have already been excluded in the sample figures reported in the text and tables.

4 Computing averages across countries that standardize within-country is conceptually identical to performing a main effect linear mixed model analysis that accounts for the country-level nesting of the data, because both methods remove across-nation variance. As can be seen by comparing Column 1 from Tables 8, 9 with the summary scores in Tables 2–7, the methods essentially produce the same results. Thus, the two alternative approaches to the same conceptual ends produce the same results.

5 Like Conway et al. (2021b), we excluded Psychological Impacts from this measure due to its overlap with the DV. See Conway et al. (2021b) for further information.

6 We also tested for curvilinear effects for age. These additional results generally suggested curvilinear effects for both Impacts and Experiences (but not for Government Response). However, the Impacts and Experiences quadratic effects generally represented a curve that would validate the basic conclusions of the linear effects, as they suggested that the effects of age became even more pronounced (less impact, less experience) at greater ages, while the corresponding drop in younger persons (at the other end of the curve) was comparatively less pronounced. It is beyond the scope of this article to pursue this issue in depth.

7 For weighted averages for age and gender, all significant effects hold when controlling for the other variable (i.e., age controlling for gender and gender controlling for age), and effect sizes are essentially unchanged. In this study, the two variables generally operate independently.

8 We focus here and in the tables on the variables for which theory seems most relevant (and for which, on average, there were more culture-specific effects): threat, experiences, and impacts. We also analyzed government response items and those are presented in Supplementary material for completeness.

9 We performed all PAIR analyses controlling for age and biological sex. Controlling for age and biological sex did not alter any of the key effects.
