# Supplementary material for: How culturally unique are pandemic effects? Evaluating cultural similarities and differences in effects of age, biological sex, and political beliefs on COVID impacts
Source: Front Psychol. 2022 Dec 19;13:937211. doi: 10.3389/fpsyg.2022.937211 (PMC9807227; doi:10.3389/fpsyg.2022.937211)
Supplement: Supplementary file 1 [file Table_1.DOCX]

**How Culturally-Bound are Pandemic Effects?: Evaluating Cultural Similarities and Differences in Effects of Age, Biological Sex, and Political Beliefs on COVID Impacts**

**Online Supplement: Additional Results, Appendices, Additional Tables, Additional Information about Each Sample and Factor Analyses**

**Preliminary Study for Scale Development**

We developed and tested a battery of face-valid social psychological questionnaires designed to measure various social psychological aspects of COVID-19 across multiple cultures. We focused on four key areas that are central to the social psychology of pandemics (see Van Bavel et al., 2020): *Perceived Threat, Desired Governmental Response, Impacts,* and *Experiences.* We then use these questionnaires to test relationships between COVID-19 beliefs/experiences with age and biological sex assigned at birth across 18 nations. Finally, we use these questionnaires to provide a novel cross-cultural test of an emerging model of the socio-political nature of pathogen outbreaks. Cumulatively, this set of data will help further our understanding of the cross-cultural psychology of disease outbreaks.

**Construct Development**

Construct development (see, e.g., Clark & Watson, 2019; Leung et al., 2011) was driven by four sets of theory-based questions that are central to understanding the cross-cultural psychology of a pandemic (Van Bavel et al., 2020): (1) How threatening do people around the world view COVID-19? This is one of the most central psychological aspects to any disease spread (Van Bavel et al., 2020), and yet we currently have no validated cross-cultural measurements to fully evaluate it across multiple nations. (2) Do people want their government to enforce social distancing, support vaccine research, or offer stimulus packages? (3) Do people experience different levels of psychological distress, financial loss, or resource deprivation? (4) Do people report different levels of experience with COVID-19? Currently, our social psychological understanding of these vital questions is hindered by a lack of validated cross-cultural measurements for evaluating the psychology of disease. We first address this obstacle by developing questionnaires in one context (preliminary study), validating the psychometric properties of those questionnaires in multiple other contexts around the world (Primary study), and then use these questionnaires to test hypotheses about the social psychology of pandemics (Primary study – see main text).

**Process Followed in Scale Development**

The process followed for scale development balanced scientific rigor with the need to produce scales as quickly as possible during the pandemic. As described in more detail below, in Samples 1-3 we produced an initial item pool for each scale. In these samples, we recruited participants in one cultural context (the United States) to complete the scales. We ran exploratory factor analyses (EFAs; Sample 1) on the scales and subsequently removed items that were ineffective, while adding additional items as necessary. In Samples 2 and 3, we performed confirmatory factor analyses (CFAs), which validated the final scales. These final questionnaires were subsequently evaluated by the Disaster Information Management Research Center at the United States National Institutes of Health (NIH) and deemed worthy to post on their Research Tools website for other researchers to use (Disaster Information Management Research Center, 2020).

This initial work was a build-up to the primary study. In the primary study, participants completed these validated final scales as a part of multiple projects in national locales across six continents.^[[1]](#footnote-1)^ We used this large dataset to test cross-cultural hypotheses about age, biological sex, and the Perceived Anxiety-Ideology Relationship (PAIR) model of cross-cultural pandemic psychology (Primary study). The PAIR model focuses on the psychological lens through which persons view a disease outbreak. In particular, it highlights the importance of the perceived *match* between desired ideological outcomes and the properties of a given outbreak (in this case, a pandemic), and suggests that people evaluate disease outbreaks differently if they have different desired ideological outcomes. As described, the tests in the primary study provided novel cross-cultural evidence for two of the basic predictions of the PAIR model of pandemic psychology.

**Sample 1 Methods**

For the sake of brevity, we provide narrative summaries of the methods and subsequent item analyses for Samples 1-3. In all cases, however, the complete list of initial items and factor loadings for each study can be found in the Supplementary Tables.

**Participants**

In Sample 1, Amazon Mechanical Turk (MTurk) participants (*n* = 279)^[[2]](#footnote-2)^ completed a battery of items we viewed as related to Perceived Coronavirus Threat, Desired Governmental Response, Impacts, and Experiences. Given recently identified potential issues with MTurk (e.g., Kennedy et al., 2019), across Samples 1-3, we ensured the highest quality of data by including several screener questions (e.g., “I am a human being” followed by a long list of possible answers) that participants had to answer correctly to be included in the study. Evidence suggests that MTurk still produces excellent data when such safeguards are applied (Kennedy et al., 2019). Our analyses suggest that efforts to ensure data quality were successful.

**Perceived Coronavirus Threat Questionnaire**

Participants completed six items concerning how threatened or worried they were about COVID-19, for example, “Thinking about the coronavirus (COVID-19) makes me feel threatened.” All measurements used a 1-7 rating scale anchored by "not true of me at all" (1) and "very true of me" (7).

**Governmental Response to Coronavirus Questionnaire**

Participants completed 48 items concerning how they felt about their government response to the crisis. These 48 items represented 6 dimensions across 3 layers of government (Federal, State, City). For each dimension, participants completed two questions that were parallel for each level of government. For example, for the Restriction dimension, the item, “I support Federal government measures to restrict the movement of American citizens to curb the spread of Coronavirus (COVID-19)” measured Federal-level restriction. The same item at the state-level read, “I support State government measures to restrict the movement of American citizens to curb the spread of Coronavirus (COVID-19),” and at the city-level, the item read, “I support City government measures to restrict the movement of American citizens to curb the spread of Coronavirus (COVID-19).” The six dimensions are discussed in more detail below. All measurements used a 1-7 rating scale anchored by "not true of me at all" (1) and "very true of me" (7).

**Restriction.** *Restriction* questions measured the degree to which participants wanted their Federal, State, and City governments to restrict citizens behavior to help stop the spread of the virus. A sample question at the Federal level is, “I support Federal government measures to restrict the movement of American citizens to curb the spread of Coronavirus (COVID-19).”

**Punishment.** *Punishment* questions measured the degree to which participants wanted their Federal, State, and City governments to punish citizens who violated social distancing rules. A sample question at the Federal level is, “I want my Federal government to severely punish those who violate orders to stay home.”

**Reactance.** *Reactance* questions measured the degree to which participants felt angry that their Federal, State, and City governments were taking away their freedom during the crisis. These items were adapted from prior work on political reactance (e.g., Conway & Repke, 2019; Conway et al., 2017). A sample question at the Federal level is, “I am upset at the thought that my Federal government would force people to stay home against their will.”

**Research.** *Research* questions measured the degree to which participants wanted their Federal, State, and City governments to fund research on the virus. A sample question at the Federal level is, “I think we should spend most of our Federal resources right now towards finding a vaccine (or other medical cure) for Coronavirus (COVID-19).”

**Stimulus.** *Stimulus* questions measured the degree to which participants wanted their Federal, State, and City governments to give stimulus money back to individuals to help the economy. A sample question at the Federal level is, “I think it is a good idea for the Federal government to give individual citizens money back during these difficult times to increase spending and keep business going.”

**Informational Contamination.** *Informational Contamination* questions measured the degree to which participants felt that they could not trust their Federal, State, and City governments to provide accurate information during the crisis. These items were adapted from prior work on political informational contamination (e.g., Conway & Repke, 2019; Conway et al., 2017). A sample question at the Federal level is, “I distrust the information I receive about the Coronavirus (COVID-19) from my Federal government.”

**Coronavirus Experiences and Impacts Questionnaire**

Participants completed 14 items concerning their experiences with and impacts of COVID-19. All measurements used a 1-7 rating scale anchored by "not true of me at all" (1) and "very true of me" (7). The questions stemmed from several conceptual dimensions: Whether participants might have had COVID-19 or other related diseases recently (“I have been diagnosed with coronavirus (COVID-19)”), whether they might have known others who had COVID-19 (“I know someone who has had coronavirus-like symptoms in the last two months”), how much COVID-19 news they had been consuming (“I watch a lot of news about the Coronavirus (COVID-19)”), how they had been financially impacted (“I have lost job-related income due to the Coronavirus (COVID-19)”), and how they had been psychologically impacted (“The Coronavirus (COVID-19) outbreak has impacted my psychological health negatively”). As is evident below, although conceptually related, these questions did not cohere into a single questionnaire with excellent psychometric properties. Consequently, we split the Coronavirus Experiences and Impacts Questionnaire into two measures.

**Sample 1 Results and Discussion**

For all questionnaire groups with more than one component factor, we performed a Principal Components EFA with a Varimax rotation, with a cutoff of an Eigenvalue of 1 for each component.

**Perceived Coronavirus Threat Questionnaire**

Analysis on the Perceived Coronavirus Threat Questionnaire yielded one component factor, with a total variance accounted for of 67.9%.

**Governmental Response to Coronavirus Questionnaire**

Analysis of the Government Response to Coronavirus Questionnaire yielded seven component factors with a total variance accounted for of 80.6%. Generally, these factors matched the six-dimensional structure we had intended. There were two exceptions. Primarily, the second *Punishment* item loaded onto the *Restriction* factor, rather than the *Punishment* factor. For Sample 2, this item was replaced with a new item designed to better tap into punishment specifically. Additionally, item analyses relevant to the seventh factor clearly suggested that this additional factor occurred in part due to some differences across levels of government. As a result, for Sample 2 we split the Governmental Response questionnaire into three separate questionnaires devoted to participants’ views of the response of their Federal, State, and City governments, respectively.

**Coronavirus Experiences and Impacts Questionnaire**

The Experiences and Impacts Questionnaire yielded five component factors with a total variance accounted for of 73.3%. The clearest factors were for Psychological Impacts, Financial Impacts, and News Watching. The questions concerning one’s own experience with the disease and experience with others seemed to split into two components. Further, the resource-related question did not load on financial impacts as anticipated, but on psychological impacts (though not as strongly as the expected psychological impacts items). As a result, we developed a separate resource impacts subscale for Sample 2.

**Sample 2 Methods**

**Participants**

In Sample 1b, two hundred and seventy five MTurk participants completed a similar battery of items as Sample 1 participants, with identical data quality safeguards as used in Sample 1.

**Differences from Sample 1**

Sample 1b questionnaires were identical to Sample 1 questionnaires except for the following changes. First, we removed the failed *Punishment* item from the Governmental Response Questionnaire and replaced it (at all three governmental levels) with a new item that more clearly measured punishment. Secondly, we expanded the Coronavirus Impacts and Experiences questionnaire by (a) adding additional con-trait (i.e., reverse-scored) items to several of the presumed subscales, and (b) adding two additional items with the goal of creating a *Resource Acquisition* subscale.

**Sample 2 Results and Discussion**

Informed by Sample 1, Sample 2 Confirmatory Factor Analyses (CFAs) were conducted to more directly test the fit of the expected factor structures. CFAs are a stringent method of substantiating factor structures because of high-standards fit indices (Costello & Osborne, 2005). The CFAs were conducted using the Lavaan package for *R* (Rosseel, 2012). Items were standardized, and the maximum likelihood estimation with full information maximum likelihood (FIML) method was used to manage missing data (Li, 2016).

**Perceived Coronavirus Threat Questionnaire**

Using CFA, the Perceived Coronavirus Threat Questionnaire showed excellent psychometric properties. The Comparative Fit Index (CFI = .98) and the Tucker-Lewis Index (TLI = .97) surpassed both the typically accepted minimums (.90) and the typically accepted standards for a “good” fit of the measure to the data (.95; Lai & Green, 2016; Schreiber et al., 2006). Furthermore, the Root Mean Square Error of Approximation (RMSEA = .09, 90% CI = .05, .13, *p* = .04) and the Standardized Root Mean Square Residual (SRMR = .03) also showed good fit; the typical standards for adequate RMSEAs and SRMRs are .10 and below, with .05 and below indicating a “good” fit (Lai & Green, 2016). This model has both good relative fit (i.e., performs better than a hypothesized null model, as indicated by the CFI and TLI) and adequate-to-good absolute fit (i.e., reproduces the data with little error, as indicated by the RMSEA and SRMR).

**Governmental Response to Coronavirus Questionnaire**

The CFA for the Governmental Response to Coronavirus Questionnaires six-factor structures demonstrated strong fits to the data (Federal: CFI = .99, TLI = .98, RMSEA = .05, 90% CI = .02, .03, *p* = .034, SRMR = .03; State: CFI = .99, TLI = .98, RMSEA = .05, 90% CI = .03, .07, *p* = .48, SRMR = .02; City: CFI = .99, TLI = .98, RMSEA = .06, 90% CI = .04, .08, *p* = .14, SRMR = .03). Thus, used as separate scales for each level of government, the six-factor structure holds.

**Coronavirus Experiences and Impacts Questionnaire**

Consistent with Sample 1, the Coronavirus Experiences and Impacts Questionnaire demonstrated a substandard fit to the Sample 2 data (CFI = .82, TLI = .77, RMSEA = .11, 90% CI = .10, .11, *p* < .001, SRMR = .09). To better understand why and seek guidance for Sample 3, we examined factor loadings across the scales. This led us to drop two of the con-trait items and to split the questionnaire up into two smaller questionnaires using a theory-based approach: We separated participants’ *Impacts* of COVID-19 (e.g., reports of how it had affected their lives) and their *Experiences* with the disease (e.g., reports of their own and others’ symptoms, their own experience seeing news about it) into two scales. The Impacts scale comprised Financial Impacts, Resource Impacts, and Depression Impacts, and the Experiences scale included Personal Diagnoses/Symptoms, Close Other Diagnosis/Symptoms, and COVID-19 News Exposure. Fit improved for both scales, but more so for the Impacts scale (CFI = .94, TLI = .90, RMSEA = .12, 90% CI = .10, .14, *p* < .001, SRMR = .06), which demonstrated an overall adequate fit. However, the indices for the Experiences questionnaire Experiences (CFI = .78, TLI = .69, RMSEA = .16, 90% CI = .14, .18, *p* < .001, SRMR = .09) were still below typical standards.

**Primary Investigation: Factor Analysis (Additional Results)**

To test the cross-cultural validity of the factor structures of the Perceived Coronavirus Threat, Federal Governmental Response to Coronavirus, Coronavirus Impacts, and Coronavirus Experience Questionnaires, these four measures were administered to individuals in 48 countries across 19 separate studies. Confirmatory Factor Analyses (CFAs) were conducted to assess whether or not the factor structure of each scale held across different countries (Costello & Osborne, 2005). The CFAs were conducted using the Lavaan package for *R* (Rosseel, 2012). Items were standardized within dataset, and the maximum likelihood estimation with full information maximum likelihood (FIML) method was used for missing data (Li, 2016). For each questionnaire, we report analyses on the whole sample, on short and long versions separately, and by nation. We additionally performed analyses on each dataset separately and for each nation within each dataset. These analyses generally confirmed the results of the main analyses and thus we do not report them for the sake of brevity; however, the complete analyses can be found in the online supplementary materials.

**Factor Analyses: Perceived Coronavirus Threat Questionnaire**

A Confirmatory Factor Analysis (CFA) on the Perceived Coronavirus Threat Questionnaire using the full combined dataset (*N* = 11,882) produced a CFI (.989) and TLI (.982) that indicated a strong fit of the measure to the data (Lai & Green, 2016; Schreiber et al., 2006). The RMSEA (.051, 90% CI = .046, .056, *p* = .350) and the SRMR (.030) indicated good fit to the data based on the typical standards for RMSEAs and SRMRs (Lai & Green, 2016; Schreiber et al., 2006). Thus, the Perceived Coronavirus Threat Questionnaire has both good relative fit (i.e., performs better than a hypothesized null model, as indicated by the CFI and TLI) and absolute fit (i.e., reproduces the data with little error, as indicated by the RMSEA and SRMR).

When a CFA was conducted on the Threat Questionnaire using only data from studies that administered the full Threat Questionnaire (*N* = 2,238), the CFI (.959), TLI (.931), RMSEA (.110, 90% CI = .099, .122, *p* < .001), and SRMR (.029) indicated that this measure has an adequate-to-good relative fit to the data and an overall adequate absolute fit to the data (RMSEA was slightly above the typically accepted maximum for adequate fit, well within the typically accepted range for good fit).

The short version of the Perceived Coronavirus Threat Questionnaire consists of three items on a single factor and is thus a saturated or “just identified” model (e.g., Kaplan, 2009; see also UCLA Statistical Consulting Group, 2020). Such models do not have any degrees of freedom with which to calculate CFA fit indices. In the case of a saturated model, one can look to other indicators of model fit, such as the factor loadings and standardized regression coefficients for each of the three items. Using the full combined dataset, each of the 3 Threat items on the short questionnaire was found to have very strong factor loadings (1.672, 1.728, and 1.809) and standardized regression coefficients (ranging from .832, .872, and .908). This suggests that the short Perceived Coronavirus Threat Questionnaire has a good fit to the data (Young & Pearce, 2013).

CFAs were then conducted on the Perceived Coronavirus Threat Questionnaire for each country that had at least 20 participants across all studies (combining data from separate studies). CFAs were conducted on the full Threat Questionnaire unless all or nearly all participants from a country were administered the short Threat Questionnaire. CFAs for the full Threat Questionnaire were calculated and found adequate to strong fits to the data (see Table 2 of supplementary materials). CFAs on countries that were only administered the short Threat Questionnaire also found good fits to the data within-country (based on factor loadings and standardized regression coefficients; see Table 3 of Supplementary materials). Thus, whether looking (1) across-country or within-country or (2) at the long or short form, the Threat Questionnaire had generally excellent psychometric properties.

**Factor Analyses: Federal Governmental Response to Coronavirus Questionnaire**

A CFA on the Federal Governmental Response to Coronavirus Questionnaire using the full combined dataset (*N* = 6,310) produced a CFI of .983 and TLI of .971, indicating a strong relative fit of the measure to the data. The RMSEA (.046, 90% CI = .042, .049, *p* = .980) and the SRMR (.029) indicated good absolute fit to the data.

CFAs were then conducted on the Federal Governmental Response to Coronavirus Questionnaire using data from each individual country that had at least 20 participants across all studies (combining data from separate studies). CFAs on these countries found adequate to strong fits to the data (see Table 4 of supplementary materials).

**Factor Analyses: Coronavirus Impacts Questionnaire**

A CFA on the Coronavirus Impacts Questionnaire using the full combined dataset produced a CFI of .994, TLI of .992, RMSEA of .028 (90% CI = .025, .031, *p* = .999), and SRMR of .055, together indicating an excellent fit of the measure to the data.

When a CFA was conducted on the full Impacts Questionnaire using only data from studies that administered the full Impacts Questionnaire (*n* = 1,186), the CFI (.953), TLI (.930), RMSEA (.068, 90% CI = .058, .079, *p* = .002), and SRMR (.053) suggested adequate-to-good fit to the data.

CFA on the short version of the Coronavirus Impacts Questionnaire using the full combined dataset found a CFI of .997, TLI of .994, RMSEA of .037 (90% CI = .031, .043, *p* = 1.000), and SRMR of 0.008, indicating a strong fit to the data.

CFAs were then conducted on the Coronavirus Impacts Questionnaire using data from each individual country that had at least 20 participants across all studies (combining data from separate studies). CFAs on the full Impacts Questionnaire found an adequate-to-strong fit to the data, with the exception of Germany, which is likely due to its small *N* (*n* = 32, see Table 5 of supplementary materials). CFAs were also conducted on the short Impacts Questionnaire using countries that were entirely or almost entirely administered the short scale. These CFAs generally showed strong fit (see Table 6 of supplementary materials).

**Factor Analyses: Coronavirus Experiences Questionnaire**

A CFA on the full Coronavirus Experiences Questionnaire using the full combined dataset demonstrated an adequate-to-good fit of the measure to the data (CFI = .963; TLI = .948; RMSEA = .061, 90% CI = .059, .064, *p* < .001; SRMR = .069).

A CFA on the full Coronavirus Experiences Questionnaire using only the data from studies that administered the full Experiences Questionnaire (*N* = 1,022) produced fit indices indicating an overall subpar fit of the measure to the data (CFI = .724; TLI = .612; RMSEA = .114, 90% CI = .105, .124, *p* < .0001; SRMR = .069).

A CFA on the short version of the Coronavirus Experiences Questionnaire using the full combined dataset yielded a CFI of .975, TLI of .952, RMSEA of .085 (90% CI = .081, .090, *p* < .001), and an SRMR of .023. These suggest that the short Experiences Questionnaire has an overall good fit to the data.

Next, CFAs were conducted on the Coronavirus Experiences Questionnaire using data from each individual country that had at least 20 participants across all studies (combining data from separate studies). CFAs on the full Experiences Questionnaire produced mixed results, with just over half of the indices demonstrating an adequate-to-strong fit and just under half demonstrating an inadequate fit (see Table 7 of supplementary materials). CFAs were also conducted on the short Experiences Questionnaire using countries that were entirely or almost entirely administered the short scale. These CFAs generally showed an adequate-to-good fit of the data (see Table 8 of supplementary materials).

**Supplementary Tables**

Table 1: Scale Alphas for Long and Short Versions of Each Scale

________________________________________________________________________

Scale Long Short

____________________________________________________________________________

Perceived Coronavirus Threat .88 .90

Federal Government Response:

Restriction .87 n/a

Punishment .93 n/a

Reactance .86 n/a

Research .71 n/a

Stimulus .89 n/a

Informational Contamination .85 n/a

State Government Response:

Restriction .89 n/a

Punishment .95 n/a

Reactance .91 n/a

Research .88 n/a

Stimulus .94 n/a

Informational Contamination .90 n/a

City Government Response:

Restriction .88 n/a

Punishment .95 n/a

Reactance .94 n/a

Research .93 n/a

Stimulus .94 n/a

Informational Contamination .92 n/a

Impacts:

Financial .81 .76

Resource .86 .93

Psychological .81 .89

Experiences:

Personal Diagnosis/Symptoms .66 n/a

Proximity Others .79 .71

News .65 .64

________________________________________________________________________

Note: *N* = 413.

____________________________________________________________________________

Table 2: Perceived Coronavirus Threat Scale by Nation, Full version

| Country | CFI | TLI | RMSEA | SRMR |
| --- | --- | --- | --- | --- |
| United Kingdom (N = 2,214) | .994** | .990** | .036** (90% CI = .023, .049, *p* = .967) | .041** |
| Germany (N = 36) | .941* | .902* | .112 (90% CI = .000, .234, *p* = .216) | .082* |
| Austria (N = 67) | .965** | .942* | .083* (90% CI = .000, .173, *p* = .256) | .045** |
| Italy (N = 472) | .973** | .954** | .075* (90% CI = .049, .104, *p* = .057) | .056* |
| USA (N = 1,498) | .976** | .961** | .083* (90% CI = .069, .098, *p* = .000) | .033** |
| Poland (N = 723) | .988** | .980** | .055* (90% CI = .033, .078, *p* = .319) | .021** |
| Turkey (N = 2,181) | .993** | .988** | .040** (90% CI = .028, .053, *p* = .887) | .027** |
| Mexico (N = 4,399) | .993** | .988** | .039** (90% CI = .030, .048, *p* = .983) | .067* |

* Indicates an adequate fit ** Indicates a good fit

Table 3: Perceived Coronavirus Threat Scale by Nation, Short version

| Country | Factor Loadings | Standardized Regression Coefficients |
| --- | --- | --- |
| Greece (N = 104) | 1.276, 1.334, 1.633 | .760, .812, .981 |
| India (N = 64) | 1.360, 1.363, 1.373 | .835, .866, .932 |
| Brazil (N = 23) | 1.169, 1.500, 1.701 | .640, .862, .952 |
| Canada (N = 23) | 1.380, 1.716, 1.785 | .744, .889, .963 |
| Australia (N = 52) | 1.380, 1.554, 1.676 | .841, .898, .930 |

Table 4: Governmental Response to Coronavirus by Nation

| Country | CFI | TLI | RMSEA | SRMR |
| --- | --- | --- | --- | --- |
| United Kingdom (N = 2,199) | .987** | .978** | .040** (90% CI = .034, .046, *p* = .996) | .022** |
| Greece (N = 103) | .940* | .899 | .101 (90% CI = .069, .132, *p* = .007) | .074* |
| USA (N = 1,043) | .983** | .970** | .049** (90% CI = .040, .058, *p* = .562) | .029** |
| Poland (N = 721) | .975** | .958** | .049** (90% CI = .038, .061, *p* = .518) | .035** |
| Turkey (N = 1,879) | .971** | .950** | .063* (90% CI = .057, .069, *p* = .000) | .041** |
| Guinea West Africa (N = 240) | .998** | .995** | .015 (90% CI = .000, .075, p = .771) | .028** |
| India (N = 62)^a^ | .962** | .936* | .089* (90% CI = .033, .135, *p* = .105) | .052* |
| Brazil (N = 23)^b^ | .906* | .841 | .124 (90% CI = .000, .203, *p* = .113) | .130 |

*Indicates an adequate fit. **Indicates a good fit. ^a^Covariation matrix is not positive definite, suggesting there might be multicollinearity. Results should be treated with caution. We included them here for completeness. ^b^Data does not meet traditional participants-per-item ration and thus should be treated with caution. We include them here for completeness.

Table 5: Coronavirus Impacts Questionnaire by Nation, Full Version

| Country | CFI | TLI | RMSEA | SRMR |
| --- | --- | --- | --- | --- |
| UK (N = 2,205) | .994** | .991** | .030** (90% CI = .022, .038, *p* = 1.000) | .046** |
| Germany (N = 32) | .866 | .799 | .142 (90% CI = .051, .219, *p* = .049) | .129 |
| Austria (N = 67) | .942* | .913* | .106 (90% CI = .048, .158, *p* = .054) | .076* |
| Italy (N = 852) | .990** | .985** | .032** (90% CI = .017, .046, *p* = .982) | .040** |
| USA (N = 1,107) | .981** | .972** | .047** (90% CI = .032, .058, *p* = .644) | .041** |
| Poland (N = 721) | .953** | .929* | .073* (90% CI = .060, .086, *p* = .002) | .064* |

* Indicates an adequate fit ** Indicates a good fit

Table 6: Coronavirus Impacts Questionnaire by Nation, Short Version

| Country | CFI | TLI | RMSEA | SRMR |
| --- | --- | --- | --- | --- |
| Greece (N = 104) | 1.000** | 1.005** | .000** (90% CI = .000, .128, *p* = .642) | .019** |
| Turkey (N = 1,885) | .997** | .992** | .045** (90% CI = .030, .062, *p* = .649) | .012** |
| Mexico (N = 4,128) | .990** | .976** | .070* (90% CI = .060, .081, *p* = .001) | .024** |
| Slovenia (N = 265) | .995** | .986** | .049** (90% CI = .000, .102, *p* = .443) | .019** |
| Portugal (N = 254) | .963** | .909* | .120 (90% CI = .077, .166, *p* = .005) | .026** |
| Lithuania (N = 274) | .999** | .998** | .022** (90% CI = .000, .084, *p* = .698) | .015** |
| China (N = 208) | .960** | .900* | .118 (90% CI = .070, .170, *p* = .012) | .043** |

* Indicates an adequate fit ** Indicates a good fit

Table 7: Coronavirus Experiences Questionnaire, Full Version

| Country | CFI | TLI | RMSEA | SRMR |
| --- | --- | --- | --- | --- |
| UK (N = 2,199) | .959** | .942* | .075* (90% CI = .069, .081, *p* = .000) | .229 |
| USA (N = 1,102) | .909* | .873 | .090* (90% CI = .081, .099, *p* = .000) | .113 |
| Poland (N = 721) | .873 | .821 | .095* (90% CI = .084, .106, *p* = .000) | .076* |

* Indicates an adequate fit ** Indicates a good fit

Table 8: Coronavirus Experiences Questionnaire, Full Version, Short Version

| Country | CFI | TLI | RMSEA | SRMR |
| --- | --- | --- | --- | --- |
| Greece (N = 103) | .922* | .852 | .127 (90% CI = .072, .184, *p* = .014) | .048** |
| Italy (N = 712) | .978** | .957** | .079* (90% CI = .061, .100, *p* = .006) | .021** |
| Turkey (N = 1,879) | .989** | .980** | .046** (90% CI = .035, .059, *p* = .662) | .027** |
| Mexico (N = 4,127) | .958** | .921* | .084* (90% CI = .077, .092, *p* = .000) | .043** |
| India (N = 62) | 1.000** | 1.024** | .000** (90% CI = .000, .080, *p* = .892) | .014** |
| Slovenia (N = 260) | .999** | .997** | .013** (90% CI = .000, .067, *p* = .828) | .030** |
| Portugal (N = 253) | .933* | .871 | .091* (90% CI = .058, .127, *p* = .024) | .062* |
| Lithuania (N = 273) | .980** | .962** | .059* (90% CI = .018, .096, *p* = .311) | .046** |
| China (N = 207) | .889 | .787 | .173 (90% CI = .138, .210, *p* = .000) | .079* |

* Indicates an adequate fit ** Indicates a good fit

Table 9:  Explaining Culture-Level Variance: Weighted Correlations Between Culture-Level Socioecological Variables and the relationships of Age with Outcomes

|  | Pathogens | | | Cold Stress | | | Heat Stress | | | GDP/PC | |  |
| --- | --- | --- | --- | --- | --- | --- | --- | --- | --- | --- | --- | --- |
| Threat | | -.55 | | | .19 | | | .33 | | .20 | |  |
| Impacts | |  | | |  | | |  | |  | |  |
| Financial Impacts | | .08 | | | -.29 | | | -.25 | | -.42 | |  |
| Resource Impacts | | -.56 | | | .23 | | | -.11 | | .27 | |  |
| Psychological Impacts | | -.43 | | | -.06 | | | .18 | | .08 | |  |
| Experiences | |  | | |  | | |  | |  | |  |
| Personal | | -.16 | | | -.22 | | | -.19 | | -.21 | |  |
| Other | | -.33 | | | -.28 | | | -.05 | | -.13 | |  |
| News | | -.50 | | | .12 | | | .26 | | .49 | |  |
| Government Response | |  | | |  | | |  | |  | |  |
| Restriction | | .12 | | | .09 | | | -.02^^ | | .05 | |  |
| Punishment | | .42 | | | -.75 | | | .17 | | -.86 | |  |
| Reactance | | -.02^^ | | | -.63 | | | -.15 | | -.77 | |  |
| Research | | .27 | | | -.45 | | | .10 | | -.50 | |  |
| Stimulus | | -.52 | | | .20 | | | -.09 | | .12 | |  |
| Informational Contamination | | -.48 | | | .21 | | | -.54 | | .10 | |  |
|  | |  |  | | |  | | |  | |  | |

*Note*. All correlations weighted by sample size. All correlations significant at *p* <= .001 unless otherwise noted. ^*p* < .01. ^^*p* > .05. Higher scores mean that cultures high in the variable in each column have a positive relationship between age and the variable in each row; lower scores mean that cultures high in the variable in each column have a negative relationship between age and the variable in each row. For example, the negative relationship between pathogens and threat means that cultures high in pathogens are more likely to have younger people perceive COVID as threatening than older people.

Table 10:  Explaining Culture-Level Variance: Weighted Correlations Between Culture-Level Socioecological Variables and the relationships of Age with Outcomes

|  | Inequality | | | Totalitarianism | | | Collectivism | | |  |  |
| --- | --- | --- | --- | --- | --- | --- | --- | --- | --- | --- | --- |
| Threat | | -.60 | | | -.28 | | | -.30 | |  |  |
| Impacts | |  | | |  | | |  | |  |  |
| Financial Impacts | | -.00^^ | | | .33 | | | .40 | |  |  |
| Resource Impacts | | -.49 | | | -.49 | | | -.37 | |  |  |
| Psychological Impacts | | -.58 | | | -.13 | | | -.44 | |  |  |
| Experiences | |  | | |  | | |  | |  |  |
| Personal | | -.15 | | | -.08 | | | .11 | |  |  |
| Other | | -.35 | | | -.11 | | | .02 | |  |  |
| News | | -.60 | | | -.47 | | | -.63 | |  |  |
| Government Response | |  | | |  | | |  | |  |  |
| Restriction | | -.22 | | | .08 | | | -.28 | |  |  |
| Punishment | | .02^^ | | | .72 | | | .65 | |  |  |
| Reactance | | -.29 | | | .71 | | | .56 | |  |  |
| Research | | -.09 | | | .41 | | | .25 | |  |  |
| Stimulus | | -.21 | | | -.43 | | | .60 | |  |  |
| Informational Contamination | | -.68 | | | -.08 | | | -.24 | |  |  |
|  | |  |  | | |  | | |  | |  |

*Note*. All correlations weighted by sample size. All correlations significant at *p* <= .001 unless otherwise noted. ^*p* < .01. ^^*p* > .05. Higher scores mean that cultures high in the variable in each column have a positive relationship between age and the variable in each row; lower scores mean that cultures high in the variable in each column have a negative relationship between age and the variable in each row. For example, the negative relationship between inequality and threat means that cultures high in inequality are more likely to have younger people perceive COVID as threatening than older people.

Table 11:  Explaining Culture-Level Variance: Weighted Correlations Between Culture-Level Socioecological Variables and the relationships of Biological Sex with Outcomes

|  | Pathogens | | | Cold Stress | | | Heat Stress | | | GDP/PC | |  |
| --- | --- | --- | --- | --- | --- | --- | --- | --- | --- | --- | --- | --- |
| Threat | | -.50 | | | -.09 | | | -.16 | | .40 | |  |
| Impacts | |  | | |  | | |  | |  | |  |
| Financial Impacts | | .21 | | | -.25 | | | -.55 | | .03^ | |  |
| Resource Impacts | | -.14 | | | -.48 | | | -.41 | | .34 | |  |
| Psychological Impacts | | -.38 | | | -.27 | | | .22 | | .49 | |  |
| Experiences | |  | | |  | | |  | |  | |  |
| Personal | | -.68 | | | .11 | | | -.04 | | .46 | |  |
| Other | | -.67 | | | .31 | | | -.19 | | .32 | |  |
| News | | -.05 | | | .23 | | | -.41 | | .20 | |  |
| Government Response | |  | | |  | | |  | |  | |  |
| Restriction | | .49 | | | -.34 | | | -.07 | | -.67 | |  |
| Punishment | | -.74 | | | .22 | | | -.55 | | .77 | |  |
| Reactance | | -.74 | | | .36 | | | -.52 | | .77 | |  |
| Research | | -.31 | | | -.70 | | | -.04^ | | .21 | |  |
| Stimulus | | -.06 | | | -.41 | | | -.24 | | -.39 | |  |
| Informational Contamination | | -.51 | | | .02^^ | | | -.27 | | .61 | |  |
|  | |  |  | | |  | | |  | |  | |

*Note*. All correlations weighted by sample size. All correlations significant at *p* <= .001 unless otherwise noted. ^*p* < .01. ^^*p* > .05. Higher scores mean that cultures high in the variable in each column have a positive relationship between biological sex and the variable in each row; lower scores mean that cultures high in the variable in each column have a negative relationship between biological sex and the variable in each row. For example, the negative relationship between pathogens and threat means that cultures high in pathogens are more likely to have women people perceive COVID as threatening than men.

Table 12:  Explaining Culture-Level Variance: Weighted Correlations Between Culture-Level Socioecological Variables and the relationships of Biological Sex with Outcomes

|  | Inequality | | | Totalitarianism | | | Collectivism | | |  |  |
| --- | --- | --- | --- | --- | --- | --- | --- | --- | --- | --- | --- |
| Threat | | -.61 | | | -.43 | | | -.49 | |  |  |
| Impacts | |  | | |  | | |  | |  |  |
| Financial Impacts | | .19 | | | .01^^ | | | -.06 | |  |  |
| Resource Impacts | | -.28 | | | -.37 | | | -.31 | |  |  |
| Psychological Impacts | | -.47 | | | -.47 | | | -.51 | |  |  |
| Experiences | |  | | |  | | |  | |  |  |
| Personal | | -.70 | | | -.43 | | | -.63 | |  |  |
| Other | | -.71 | | | -.33 | | | -.58 | |  |  |
| News | | .06 | | | -.21 | | | -.23 | |  |  |
| Government Response | |  | | |  | | |  | |  |  |
| Restriction | | .20 | | | .73 | | | .52 | |  |  |
| Punishment | | -.68 | | | -.75 | | | -.78 | |  |  |
| Reactance | | -.56 | | | -.81 | | | -.82 | |  |  |
| Research | | -.55 | | | -.29 | | | -.39 | |  |  |
| Stimulus | | -.16 | | | .23 | | | -.19 | |  |  |
| Informational Contamination | | -.34 | | | -.69 | | | -.67 | |  |  |
|  | |  |  | | |  | | |  | |  |

*Note*. All correlations weighted by sample size. All correlations significant at *p* <= .001 unless otherwise noted. ^*p* < .01. ^^*p* > .05. Higher scores mean that cultures high in the variable in each column have a positive relationship between biological sex and the variable in each row; lower scores mean that cultures high in the variable in each column have a negative relationship between biological sex and the variable in each row. For example, the negative relationship between inequality and threat means that cultures high in inequality are more likely to have women people perceive COVID as threatening than men.

**Main Study: Additional Factor Analyses**

To test the cross-cultural validity of the Perceived Coronavirus Threat, Federal Governmental Response to Coronavirus, Coronavirus Impacts, and Coronavirus Experience Questionnaires, these four measures were administered to individuals in 48 countries across 19 separate studies. Confirmatory Factor Analyses (CFAs) were conducted to assess whether or not the factor structure of each scale held across different countries. CFAs are a stringent method of substantiating factor structures because of the high standards of CFA fit indices (Costello & Osborne, 2005). The CFAs were conducted using the Lavaan package for R (Rosseel, 2012). Items were standardized within dataset, and the maximum likelihood estimation with full information maximum likelihood (FIML) method was used for missing data (Li, 2016).

**Factor Analyses: Perceived Coronavirus Threat Questionnaire**

A Confirmatory Factor Analysis (CFA) on the full Perceived Coronavirus Threat Questionnaire using the full combined dataset of 19 studies (N = 11,882) produced a Comparative Fit Index (CFI) of .989 and Tucker-Lewis Index (TLI) of .982, indicating a strong fit of the measure to the data (.90 and .95 being the minimum values for adequate and good fit, respectively; Lai & Green, 2016; Schreiber et al., 2006). The Root Mean Square Error of Approximation (RMSEA) = .051 (90% CI = .046, .056, *p* = .350), and the Standardized Root Mean Square Residual (SRMR) = .030. Based on the typical standards for RMSEAs and SRMRs (.10 and below for adequate fit and .05 and below for good fit; Lai & Green, 2016; Schreiber et al., 2006), these metrics indicate good fit to the data. Thus, the Perceived Coronavirus Threat Questionnaire has both good relative fit (i.e., it does much better than a hypothesized null model, as indicated by the CFI and TLI) and absolute fit (i.e., the model reproduces the data with little error, as indicated by the RMSEA and SRMR).

When a CFA was conducted on the full Threat Questionnaire using only data from studies that administered the full Threat Questionnaire (Brussels Pickett, Poland Grotkowski, Turkey Bozkurt/Mutlu, UK Farmer, Vienna Massaccesi, Mexico Nadia, USA Goldberg, and USA Tchalova; N = 2,238), the CFI = .959, TLI = .931, RMSEA = .110 (90% CI = .099, .122, *p* = .000), and SRMR = .029. Thus, this measure has an adequate to good relative fit to the data and an overall adequate absolute fit to the data (with the RMSEA slightly above the typically accepted maximum for adequate fit but the SRMR but the SRMR well within the typically accepted range for a good fit).

The short version of the Perceived Coronavirus Threat Questionnaire consists of three items on a single factor and is thus a saturated or “just identified” model (UCLA Statistical Consulting Group, 2020). Such models do not have any degrees of freedom with which to calculate fit indices. In the case of a saturated model, one can look to other indicators of model fit, such as the factor loadings and standardized regression coefficients for each of the three items. Using the full combined dataset of 19 studies (N = 11,882), each of the three Threat items on the short questionnaire were found to have very strong factor loadings (1.672, 1.728, and 1.809) and standardized regression coefficients (ranging from .832, .872, and .908). This suggests that the short Perceived Coronavirus Threat Questionnaire has a good fit to the data (Young & Pearce, 2013).

CFAs were then conducted on the Perceived Coronavirus Threat Questionnaire on each of the studies that administered this scale individually. CFAs on the studies that used the full Threat Questionnaire (Brussels Pickett, Poland Grotkowski, Turkey Bozkurt/Mutlu, UK Farmer, Vienna Massaccesi, Mexico Nadia, USA Goldberg, and USA Tchalova) found adequate to strong fits to the data (see Table 1 for specifics). CFAs on studies that used the short Threat Questionnaire (UK Papageorgiou, UK Mojtahedi, UK mTurk Mojtahedi, Mexico Tiburcio, Italy Giorgia Paleari, Turkey Ulukok, USA Szaflarski, and Australia Mackelprang) were again unable to produce fit indices, but factor loadings and standardized regression coefficients demonstrated a good fit to the data (see Table 2).

Table 1: Perceived Coronavirus Threat Scale, Full version

| Study | CFI | TLI | RMSEA | SRMR |
| --- | --- | --- | --- | --- |
| Brussels Pickett (N = 126) | .957** | .928* | .103 (90% CI = .045, .161, *p* = .064) | .040** |
| Poland Grotkowski (N = 443) | .983** | .971** | .070* (90% CI = .045, .161, *p* = .064) | .022** |
| Turkey Bozkurt/Mutlu (N = 296) | .956** | .927* | .102 (90% CI = .069, .137, *p* = .006) | .031** |
| UK Farmer (N = 301) | .970** | .949* | .085* (90% CI = .052, .121, *p* = .043) | .034** |
| Vienna Massaccesi (N = 288) | .945* | .908* | .132 (90% CI = .099, .167, *p* = .000) | .041** |
| Mexico Nadia (N = 271) | .904* | .840 | .160 (90% CI = .127, .195, *p* = .000) | .053* |
| USA Goldberg (N = 154) | .965** | .941* | .112 (90% CI = .064, .163, *p* = .021) | .034** |
| USA Tchalova (N = 359) | .937* | .896 | .152 (90% CI = .123, .182, *p* = .000) | .046** |

* Indicates an adequate fit ** Indicates a good fit

Table 2: Perceived Coronavirus Threat Scale, Short version

| Study | Factor Loadings | Standardized Regression Coefficients |
| --- | --- | --- |
| UK Papageorgiou (N = 1901) | 1.328, 1.377, 1.432 | .835, .866, .884 |
| UK Motjahedi (N = 96) | 1.486, 1.684, 1.789 | .855, .863, .937 |
| UK mTurk Motjahedi (N = 397) | 1.297, 1.385, 1.440 | .786, .834, .857 |
| Mexico Tiburcio (N = 4127) | 1.657, 1.712, 1.842 | .810, .878, .891 |
| Italy Giogia Paleari (N = 332) | 1.201, 1.389, 1.534 | .648, .832, .892 |
| Turkey Ulukok (N = 1879) | 1.614, 1.625, 1.818 | .823, .853, .936 |
| USA Szaflarski (N = 571) | 1.508, 1.575, 1.751 | .846, .855, .958 |
| Australia Mackelprang (N = 436) | 1.436, 1.506, 1.638 | .763, .830, .928 |

Next, CFAs were conducted on the Perceived Coronavirus Threat Questionnaire on each individual country that had at least 20 participants across all studies (combining data from separate studies). CFAs were conducted on the full Threat Questionnaire unless all or nearly all participants from a country were administered the short Threat Questionnaire. CFAs on the countries that were administered the full Threat Questionnaire (United Kingdom, Germany, Austria, Italy, USA, Poland, Turkey, and Mexico) found adequate to strong fits to the data (see Table 3 for specifics). CFAs on countries that were only administered the short Threat Questionnaire (Greece, India, Brazil, Canada, and Australia) found good fits to the data (based on factor loadings and standardized regression coefficients; see Table 4).

Table 3: Perceived Coronavirus Threat Scale, Full version

| Country | CFI | TLI | RMSEA | SRMR |
| --- | --- | --- | --- | --- |
| United Kingdom (N = 2,214) | .994** | .990** | .036** (90% CI = .023, .049, *p* = .967) | .041** |
| Germany (N = 36) | .941* | .902* | .112 (90% CI = .000, .234, *p* = .216) | .082* |
| Austria (N = 67) | .965** | .942* | .083* (90% CI = .000, .173, *p* = .256) | .045** |
| Italy (N = 472) | .973** | .954** | .075* (90% CI = .049, .104, *p* = .057) | .056* |
| USA (N = 1,498) | .976** | .961** | .083* (90% CI = .069, .098, *p* = .000) | .033** |
| Poland (N = 723) | .988** | .980** | .055* (90% CI = .033, .078, *p* = .319) | .021** |
| Turkey (N = 2,181) | .993** | .988** | .040** (90% CI = .028, .053, *p* = .887) | .027** |
| Mexico (N = 4,399) | .993** | .988** | .039** (90% CI = .030, .048, *p* = .983) | .067* |

* Indicates an adequate fit ** Indicates a good fit

Table 4: Perceived Coronavirus Threat Scale, Short version

| Country | Factor Loadings | Standardized Regression Coefficients |
| --- | --- | --- |
| Greece (N = 104) | 1.276, 1.334, 1.633 | .760, .812, .981 |
| India (N = 64) | 1.360, 1.363, 1.373 | .835, .866, .932 |
| Brazil (N = 23) | 1.169, 1.500, 1.701 | .640, .862, .952 |
| Canada (N = 23) | 1.380, 1.716, 1.785 | .744, .889, .963 |
| Australia (N = 52) | 1.380, 1.554, 1.676 | .841, .898, .930 |

Finally, CFAs were conducted on the Perceived Coronavirus Threat Questionnaire on each individual country that had at least 20 participants without combining data from the same country across separate studies. Two countries, India and Brazil, only appeared in one study with enough data to conduct a CFA (UK mTurk Mojtahedi) and were thus not included in these analyses on any of the four questionnaires. Again, CFAs on the countries that were administered the full Threat Questionnaire (Germany, Austria, and Italy from the Vienna Massaccesi study) found generally adequate to strong fits to the data (see Table 5 for specifics). CFAs on countries that were only administered the short Threat Questionnaire (UK and Greece from the UK Papageorgiou study, UK and USA data from the UK mTurk Mojtahedi study, USA and Poland from the USA Szaflarski study, and Australia, Canada, and USA from the Australia Mackelprang study) found, based on factor loadings and standardized regression coefficients) good fits to the data (see Table 6).

Table 5: Perceived Coronavirus Threat Scale, Full version

| Country by Study | CFI | TLI | RMSEA | SRMR |
| --- | --- | --- | --- | --- |
| Vienna Massaccesi |  |  |  |  |
| Germany (N = 30) | .919* | .865 | .127 (90% CI = .000, .260, *p* = .191) | .089* |
| Austria (N = 66) | .965** | .942* | .084* (90% CI = .000, .174, *p* = .253) | .045** |
| Italy (N = 139) | .946* | .910* | .144 (90% CI = .095, .196, *p* = .001) | .040* |

* Indicates an adequate fit

** Indicates a good fit

Table 6: Perceived Coronavirus Threat Scale, Short version

| Country by Study | Factor Loadings | Standardized Regression Coefficients |
| --- | --- | --- |
| UK Papageorgiou |  |  |
| UK (N = 1798) | 1.256, 1.310, 1.348 | .823, .861. .867 |
| Greece (N = 103) | 1.273, 1.340, 1.632 | .759, .812, .981 |
| UK mTurk Mojtahedi |  |  |
| UK (N = 31) | 1.250, 1.573, 1.954 | .781, .871, 1.006 |
| USA (N = 266) | 1.241, 1.393, 1.402 | .760, .837, .844 |
| USA Szaflarski |  |  |
| USA (N = 293) | 1.560, 1.616, 1.767 | .854, .860, .950 |
| Poland (N = 278) | 1.413, 1.451, 1.697 | .827, .842, .968 |
| Australia Mackelprang |  |  |
| Australia (N = 52) | 1.380, 1.554, 1.676 | .841, .898, .930 |
| Canada (N =23) | 1.380, 1.716, 1.785 | .744, .889, .963 |
| USA (N = 300) | 1.333, 1.417, 1.507 | .731, .808, .900 |

**Factor Analyses: Federal Governmental Response to Coronavirus Questionnaire**

A CFA on the Federal Governmental Response to Coronavirus Questionnaire using the full combined dataset (N = 6,310) produced a CFI of .983 and TLI of .971, indicating a strong relative fit of the measure to the data. The RMSEA = .046 (90% CI = .042, .049, *p* = .980), and the SRMR = .029, indicating a good absolute fit to the data.

CFAs were then conducted on the Federal Governmental Response to Coronavirus Questionnaire on each of the studies that administered this scale individually (UK Papageorgiou, UK Mojtahedi, UK mTurk Mojtahedi, Poland Grotkowski, UK Farmer, Turkey Ulukok, USA Tchalova). CFAs found adequate to strong fits to the data (see Table 7 for specifics).

Table 7: Governmental Response to Coronavirus

| Study | CFI | TLI | RMSEA | SRMR |
| --- | --- | --- | --- | --- |
| UK Papageorgiou (N = 1901) | .985** | .975** | .044** (90% CI = .038, .051, *p* = .933) | .022** |
| UK Mojtahedi (N = 96) | .985** | .974** | .047** (90% CI = .000, .089, *p* = .517) | .053* |
| UK mTurk Mojtahedi (N = 296) | .979** | .964** | .052* (90% CI = .036, .068, *p* = .386) | .029** |
| Poland Grotkowski (N = 443) | .940* | .898 | .081* (90% CI = .068, .095, *p* = .000) | .065* |
| UK Farmer (N = 301) | .949* | .914* | .063* (90% CI = .045, .081, *p* = .108) | .048** |
| Turkey Ulukok (N = 1,879) | .971** | .950** | .063* (90% CI = .057, .069, *p* = .000) | .041** |
| USA Tchalova (N = 359) | .981* | .967** | .058* (90% CI = .042, .075, *p* = .187) | .026** |

* Indicates an adequate fit

** Indicates a good fit

CFAs were then conducted on the Federal Governmental Response to Coronavirus Questionnaire using data from each individual country that had at least 20 participants across all studies (combining data from separate studies). CFAs on these countries (United Kingdom, Germany, Austria, Italy, USA, Poland, Turkey, and Mexico) found adequate to strong fits to the data (see Table 8).

Table 8: Governmental Response to Coronavirus

| Country | CFI | TLI | RMSEA | SRMR |
| --- | --- | --- | --- | --- |
| United Kingdom (N = 2,199) | .987** | .978** | .040** (90% CI = .034, .046, *p* = .996) | .022** |
| Greece (N = 103) | .940* | .899 | .101 (90% CI = .069, .132, *p* = .007) | .074* |
| USA (N = 1,043) | .983** | .970** | .049** (90% CI = .040, .058, *p* = .562) | .029** |
| Poland (N = 721) | .975** | .958** | .049** (90% CI = .038, .061, *p* = .518) | .035** |
| Turkey (N = 1,879) | .971** | .950** | .063* (90% CI = .057, .069, *p* = .000) | .041** |
| India (N = 62) | .962** | .936* | .089* (90% CI = .033, .135, *p* = .105) | .052* |
| Brazil (N = 23) | .906* | .841 | .124 (90% CI = .000, .203, *p* = .113) | .130 |

* Indicates an adequate fit ** Indicates a good fit

Next, CFAs were conducted on the Federal Governmental Response to Coronavirus Questionnaire on each individual country that had at least 20 participants without combining data from the same country across separate studies. Again, CFAs on the countries that were administered the full Threat Questionnaire found generally adequate to strong fits to the data, with the exception of the UK data from the UK mTurk Mojtahedi study, which is likely a result of an N well below the typically recommended ratio of five to ten participants per item (Comrey & Lee, 1992). See Table 9 for specifics.

Table 9: Governmental Response to Coronavirus

| Country by Study | CFI | TLI | RMSEA | SRMR |
| --- | --- | --- | --- | --- |
| UK Papageorgiou |  |  |  |  |
| UK (N = 1,798) | .986** | .977** | .042** (90% CI = .036, .049, *p* = .971) | .022** |
| Greece (N = 103) | .940* | .899 | .101 (90% CI = .069, .132, *p* = .007) | .074* |
| UK mTurk Mojtahedi |  |  |  |  |
| UK (N = 21) | .817 | .691 | .211 (90% CI = .138, .282, *p* = .001) | .144 |
| USA (N = 266) | .973** | .954** | .057* (90% CI = .037, .078, *p* = .256) | .034** |

* Indicates an adequate fit ** Indicates a good fit

**Factor Analyses: Coronavirus Impacts Questionnaire**

A CFA on the Coronavirus Impacts Questionnaire using the full combined dataset (N = 12,489) produced a CFI of .994, TLI of .992, RMSEA of .028 (90% CI = .025, .031, *p* = 1.000), and the SRMR = .055, together indicating an excellent fit of the measure to the data.

When a CFA was conducted on the full Impacts Questionnaire using only data from studies that administered the full Impacts Questionnaire (Poland Grotkowski, Turkey Bozkurt/Mutlu, UK Farmer, Vienna Massaccesi, and USA Goldberg; N = 1,186), the CFI = .953, TLI = .930, RMSEA = .068 (90% CI = .058, .079, *p* = .002), and SRMR = .053. Thus, this measure has an adequate to good fit to the data.

A CFA on the short version of the Coronavirus Impacts Questionnaire using the full combined dataset of studies (N = 12,489) found a CFI of .997, TLI of .994, RMSEA of .037 (90% CI = .031, .043, *p* = 1.000), and SRMR of 0.008, indicating a strong fit to the data.

CFAs were then conducted on the Coronavirus Impacts Questionnaire on each of the studies that administered this scale individually. CFAs on data from studies that administered the full Coronavirus Impacts Questionnaire (Poland Grotkowski, UK Farmer, Vienna Massaccesi, and USA Goldberg) found adequate to strong fits to the data (see Table 10). CFAs on the data from studies that administered the short Impacts Questionnaire (UK Papageorgiou, Mexico Tiburcio, Italy Giorgia Paleari, Turkey Ulukok, and Italy Sorgente) also found strong fits to the data (see Table 11).

Table 10: Coronavirus Impacts Questionnaire, Full Version

| Study | CFI | TLI | RMSEA | SRMR |
| --- | --- | --- | --- | --- |
| Poland Grotkowski (N = 443) | .932* | .899 | .093* (90% CI = .077, .111, *p* = .000) | .062* |
| UK Farmers (N = 301) | .977** | .966** | .053* (90% CI = .028, .077, *p* = .385) | .039** |
| Vienna Massaccesi (N = 288) | .984** | .976** | .054* (90% CI = .027, .078, *p* = .374) | .037** |
| USA Goldberg (N = 154) | .978** | .967** | .071* (90% CI = .034, .105, *p* = .154) | .044** |

* Indicates an adequate fit

** Indicates a good fit

Table 11: Coronavirus Impacts Questionnaire, Short version

| Study | CFI | TLI | RMSEA | SRMR |
| --- | --- | --- | --- | --- |
| UK Papageorgiou (N = 1,901) | .990** | .976** | .070* (90% CI = .060, .081, *p* = .001) | .023** |
| Mexico Tiburcio (N = 4,127) | .990** | .976** | .070* (90& CI = .060, .081, *p* = .001) | .023** |
| Italy Giorgia Paleari (N = 332) | 1.000** | 1.007** | .000** (90% CI = .000, .057, *p* = .922) | .015** |
| Turkey Ulukok (N = 1,879) | .997** | .992** | .045** (90% CI = .029, .062, *p* = .671) | .011** |
| Italy Sorgente (N = 1,637) | .999** | .998** | .018** (90% CI = .000, .039, *p* = .995) | .005** |

* Indicates an adequate fit ** Indicates a good fit

CFAs were then conducted on the Coronavirus Impacts Questionnaire using data from each individual country that had at least 20 participants across all studies (combining data from separate studies). CFAs on the full Impacts Questionnaire found an adequate to strong fit of the full Impacts Questionnaire to the data, with the exception of Germany, which is likely due to its small N (see Table 12). CFAs were also conducted on the short Impacts Questionnaire using countries that were entirely or almost entirely administered the short scale (Greece, Turkey, Mexico, Slovenia, Portugal, Lithuania, and China). These CFAs generally showed a strong fit of the data (see Table 13).

Table 12: Coronavirus Impacts Questionnaire, Full Version

| Country | CFI | TLI | RMSEA | SRMR |
| --- | --- | --- | --- | --- |
| UK (N = 2,205) | .994** | .991** | .030** (90% CI = .022, .038, *p* = 1.000) | .046** |
| Germany (N = 32) | .866 | .799 | .142 (90% CI = .051, .219, *p* = .049) | .129 |
| Austria (N = 67) | .942* | .913* | .106 (90% CI = .048, .158, *p* = .054) | .076* |
| Italy (N = 852) | .990** | .985** | .032** (90% CI = .017, .046, *p* = .982) | .040** |
| USA (N = 1,107) | .981** | .972** | .047** (90% CI = .032, .058, *p* = .644) | .041** |
| Poland (N = 721) | .953** | .929* | .073* (90% CI = .060, .086, *p* = .002) | .064* |

* Indicates an adequate fit

** Indicates a good fit

Table 13: Coronavirus Impacts Questionnaire, Short Version

| Country | CFI | TLI | RMSEA | SRMR |
| --- | --- | --- | --- | --- |
| Greece (N = 104) | 1.000** | 1.005** | .000** (90% CI = .000, .128, *p* = .642) | .019** |
| Turkey (N = 1,885) | .997** | .992** | .045** (90% CI = .030, .062, *p* = .649) | .012** |
| Mexico (N = 4,128) | .990** | .976** | .070* (90% CI = .060, .081, *p* = .001) | .024** |
| Slovina (N = 265) | .995** | .986** | .049** (90% CI = .000, .102, *p* = .443) | .019** |
| Portugal (N = 254) | .963** | .909* | .120 (90% CI = .077, .166, *p* = .005) | .026** |
| Lithuania (N = 274) | .999** | .998** | .022** (90% CI = .000, .084, *p* = .698) | .015** |
| China (N = 208) | .960** | .900* | .118 (90% CI = .070, .170, *p* = .012) | .043** |

* Indicates an adequate fit ** Indicates a good fit

Next, CFAs were conducted on the Coronavirus Impacts Questionnaire on each individual country that had at least 20 participants without combining data from the same country across separate studies. Again, CFAs on the countries that were administered the full Impacts Questionnaire (Germany, Austria, and Italy data from the Vienna Massaccesi study), found generally adequate to good fits to the data, with the exception of the Germany data, which is again likely a result of a small N. See Table 14. CFAs were also conducted on the short Impacts Questionnaire using countries that were entirely or almost entirely administered the short scale (UK and Greece data from UK Papageorgiou; Italy, USA, Slovina, and Portugal data from the Italy Sorgente study) These CFAs generally showed a strong fit of the data (see Table 15).

Table 14 Coronavirus Impacts Questionnaire, Full Version

| Country by Study | CFI | TLI | RMSEA | SRMR |
| --- | --- | --- | --- | --- |
| Vienna Massaccesi |  |  |  |  |
| Germany (N = 30) | .872 | .807 | .146 (90% CI = .053, .226, *p* = .047) | .125 |
| Austria (N = 66) | .941* | .912* | .106 (90% CI = .049, .159, *p* = .053) | .076* |
| Italy (N = 139) | 1.000** | 1.010** | .000* (90% CI = .000, .055, *p* = .930) | .031** |

* Indicates an adequate fit ** Indicates a good fit

Table 15: Coronavirus Impacts Questionnaire, Short version

| Country by Study | CFI | TLI | RMSEA | SRMR |
| --- | --- | --- | --- | --- |
| UK Papageorgiou |  |  |  |  |
| UK (N = 1,798) | .997** | .992** | .041** (90% CI = .025, .059, *p* = .771) | .007** |
| Greece (N = 103) | 1.000** | 1.005** | .000** (90% CI = .000, .120, *p* = .653) | .020** |
| Italy Sorgente |  |  |  |  |
| Italy (N = 379) | .987** | .968** | .067* (90% CI = .029, .108, *p* = .196) | .027** |
| USA (N = 263) | 1.000** | 1.007** | .000** (90% CI = .000, .054, *p* = .938) | .007** |
| Slovina (N = 260) | .995** | .988** | .045** (90% CI = .000, .100, *p* = .490) | .019** |
| Portugal (N = 253) | .964** | .909* | .119 (90% CI = .076, .166, *p* = .006) | .026** |

* Indicates an adequate fit ** Indicates a good fit

**Factor Analyses: Coronavirus Experiences Questionnaire**

A CFA on the full Coronavirus Experiences Questionnaire using the full combined dataset (N = 11,959) demonstrated an adequate to good fit of the measure to the data, with CFI = .963, TLI = .948, RMSEA = .061 (90% CI = .059, .064, *p* = .000), and SRMR = .069.

A CFA on the full Coronavirus Experiences Questionnaire using only the data from studies that administered the full Experiences Questionnaire (Brussels Pickett, Poland Grotkowski, UK Farmer, and USA Goldberg; N = 1,022) produced fit indices indicating an overall adequate fit of the measure to the data, with CFI = .724, TLI = .612, RMSEA = .114 (90% CI = .105, .124, *p* = .000), SRMR = .069.

A CFA on the short version of the Coronavirus Experiences Questionnaire using the full combined dataset of studies (N = 11,959) resulted in CFI = .975, TLI = .952, RMSEA = .085 (90% CI = .081, .090, *p* = .000), SRMR = .023. These suggest that the short Experiences Questionnaire has an overall good fit to the data.

CFAs were then conducted on the Coronavirus Experiences Questionnaire on each of the studies that administered this scale individually. CFAs on data from studies that administered the full Coronavirus Experiences Questionnaire (Poland Grotkowski, UK Farmer, and USA Goldberg) found generally inadequate fits to the data (see Table 16). CFAs on the data from studies that administered the short Experiences Questionnaire, however, (UK Papageorgiou, UK Mojtahedi, UK mTurk Mojtahedi, Mexico Tiburcio, Italy Giorgia Paleari, Turkey Ulukok, and Italy Sorgente) found generally adequate to strong fits to the data (see Table 17).

Table 16: Coronavirus Experiences Questionnaire, Full Version

| Study | CFI | TLI | RMSEA | SRMR |
| --- | --- | --- | --- | --- |
| Poland Grotkowski (N = 443) | .840 | .775 | .118 (90% CI = .104, .132, *p* = .000) | .085* |
| UK Farmers (N = 301) | .808 | .729 | .108 (90% CI = .091, .127, *p* = .000) | .066* |
| USA Goldberg (N = 153) | Model not positive definite | | | |

* Indicates an adequate fit

** Indicates a good fit

Table 17: Short version

| Study | CFI | TLI | RMSEA | SRMR |
| --- | --- | --- | --- | --- |
| UK Papageorgiou (N = 1,901) | .979** | .961** | .102 (90% CI = .091, .114, *p* = .000) | .021** |
| UK Motjahedi (N = 96) | 1.000** | 1.010** | .000** (90% CI = .000, .097, *p = .*732) | .035** |
| UK mTurk Motjahedi (N = 397) | .989** | .979** | .071* (90% CI = .044, .099, *p* = .097) | .017** |
| Mexico Tiburcio (N = 4,127) | .958** | .921* | .084* (90% CI = .077, .092, *p* = .000) | .043** |
| Italy Giorgia Paleari (N = 332) | .918* | .844 | .083* (90% CI = .054, .114, *p* = .032) | .054* |
| Turkey Ulukok (N = 1,879) | .989** | .980** | .046** (90% CI = .035, .059, *p* = .662) | .027** |
| Italy Sorgente (N = 1,634) | .970** | .942* | .066* (90% CI = .053, .079, *p* = .020) | .032** |

* Indicates an adequate fit

** Indicates a good fit

Next, CFAs were conducted on the Coronavirus Experiences Questionnaire using data from each individual country that had at least 20 participants across all studies (combining data from separate studies). CFAs on the full Experiences Questionnaire produced mixed results, with a little over half of the indices demonstrating an adequate to strong fit and a little under half demonstrating an inadequate fit (see Table 18). CFAs were also conducted on the short Experiences Questionnaire using countries that were entirely or almost entirely administered the short scale (Greece, Turkey, Mexico, Slovenia, Portugal, Lithuania, and China). These CFAs generally showed an adequate to good fit of the data (see Table 19).

Table 18: Coronavirus Experiences Questionnaire, Full Version

| Country | CFI | TLI | RMSEA | SRMR |
| --- | --- | --- | --- | --- |
| UK (N = 2,199) | .959** | .942* | .075* (90% CI = .069, .081, *p* = .000) | .229 |
| USA (N = 1,102) | .909* | .873 | .090* (90% CI = .081, .099, *p* = .000) | .113 |
| Poland (N = 721) | .873 | .821 | .095* (90% CI = .084, .106, *p* = .000) | .076* |

* Indicates an adequate fit ** Indicates a good fit

Table 19: Coronavirus Experiences Questionnaire, Full Version, Short Version

| Country | CFI | TLI | RMSEA | SRMR |
| --- | --- | --- | --- | --- |
| Greece (N = 103) | .922* | .852 | .127 (90% CI = .072, .184, *p* = .014) | .048** |
| Italy (N = 712) | .978** | .957** | .079* (90% CI = .061, .100, *p* = .006) | .021** |
| Turkey (N = 1,879) | .989** | .980** | .046** (90% CI = .035, .059, *p* = .662) | .027** |
| Mexico (N = 4,127) | .958** | .921* | .084* (90% CI = .077, .092, *p* = .000) | .043** |
| India (N = 62) | 1.000** | 1.024** | .000** (90% CI = .000, .080, *p* = .892) | .014** |
| Slovina (N = 260) | .999** | .997** | .013** (90% CI = .000, .067, *p* = .828) | .030** |
| Portugal (N = 253) | .933* | .871 | .091* (90% CI = .058, .127, *p* = .024) | .062* |
| Lithuania (N = 273) | .980** | .962** | .059* (90% CI = .018, .096, *p* = .311) | .046** |
| China (N = 207) | .889 | .787 | .173 (90% CI = .138, .210, *p* = .000) | .079* |

* Indicates an adequate fit

** Indicates a good fit

Lastly, CFAs were conducted on the Coronavirus Experiences Questionnaire on each individual country that had at least 20 participants without combining data from the same country across separate studies. All of these countries were entirely or almost entirely administered the short Coronavirus Experiences Questionnaire CFAs were also conducted on the short Experiences Questionnaire using countries that (UK and Greece data from the UK Papageorgiou study; UK and USA data from the UK mTurk Mojtahedi study; and Italy, USA, Slovina, and Portugal data from the Italy Sorgente study). These CFAs generally showed an adequate fit to the data (see Table 20).

Table 20: Coronavirus Experiences Questionnaire

| Country by Study | CFI | TLI | RMSEA | SRMR |
| --- | --- | --- | --- | --- |
| UK Papageorgiou |  |  |  |  |
| UK (N = 1,798) | .978* | .957* | .105 (90% CI = .094, .117, *p* = .000) | .023** |
| Greece (N = 103) | .922* | .852 | .127 (90% CI = .072, .184, *p* = .014) | .048** |
| UK mTurk Mojtahedi |  |  |  |  |
| UK (N = 21) | .917* | .841 | .209 (90% CI = .060, .343, *p* = .044) | .096* |
| USA (N = 266) | .994** | .988** | .052* (90% CI = .000, .091, *p* = .419) | .015** |
| Italy Sorgente |  |  |  |  |
| Italy (N = 378) | .965** | .932* | .070* (90% CI = .042, .099, *p* = .114) | .038** |
| USA (N = 263) | .966** | .935* | .068* (90% CI = .031, .105, *p* = .181) | .036** |
| Slovina (N = 260) | Model did not converge | | | |
| Portugal (N = 253) | .933* | .871 | .091* (90% CI = .058, .127, *p* = .024) | .062* |

* Indicates an adequate fit ** Indicates a good fit

**Appendix A: Study 3 Correlations Between Subscales**


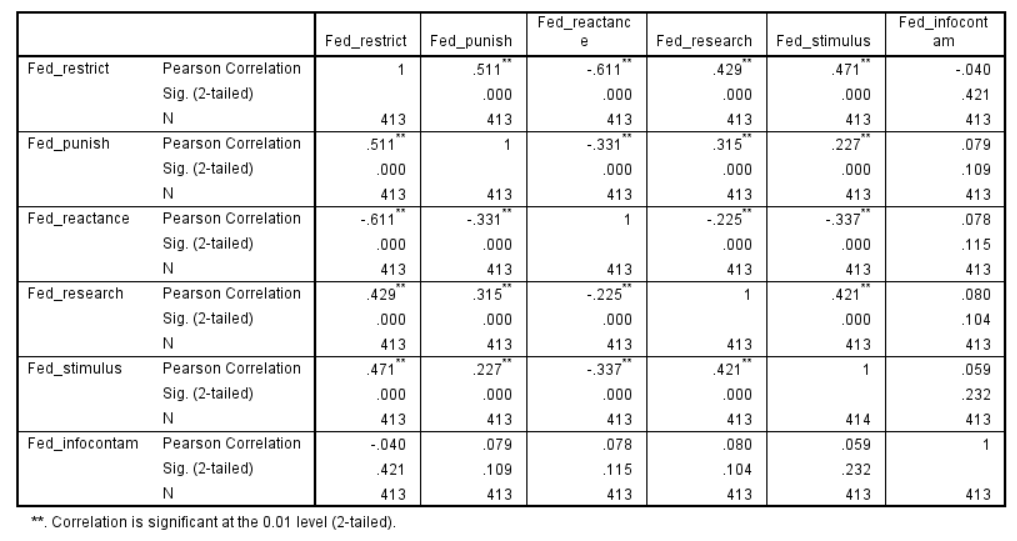

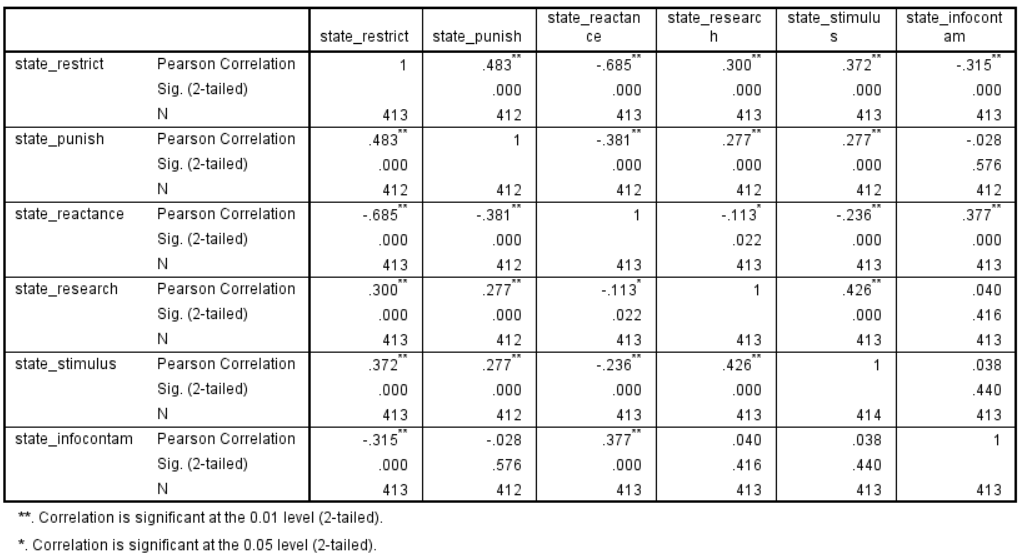

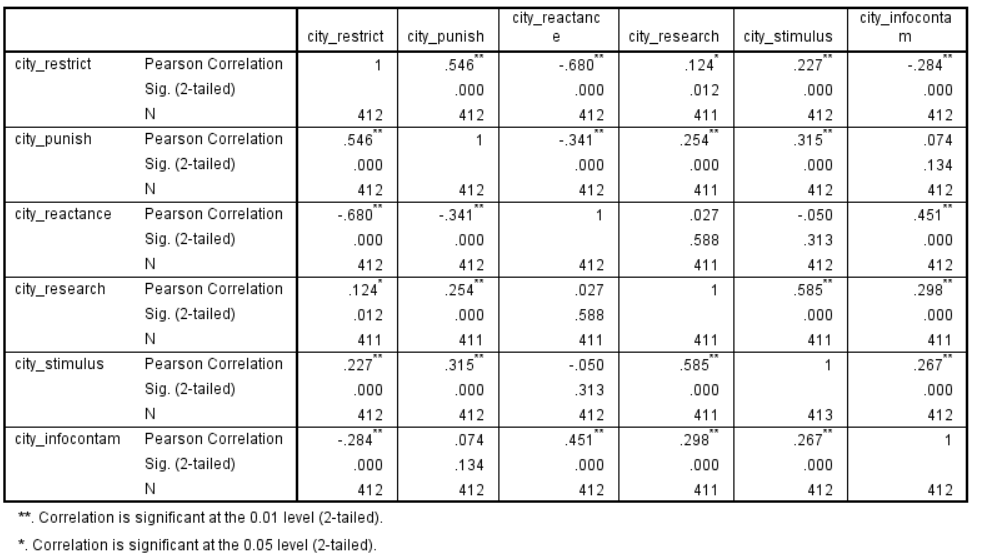

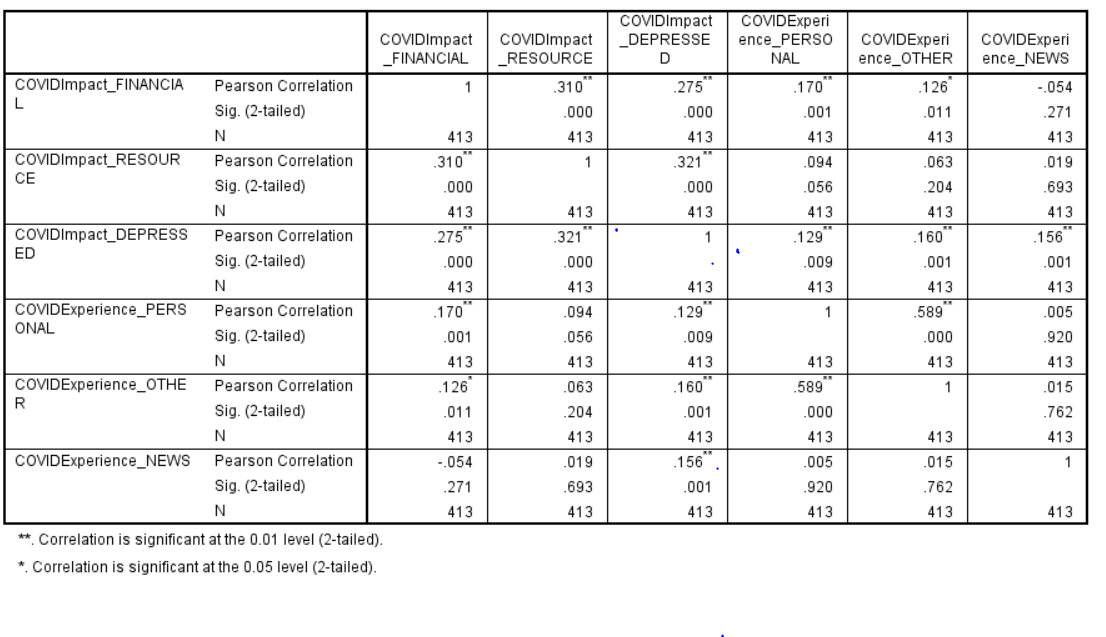


**Appendix B: Long Versions of the Scales**

Direct access to these scales on Qualtrics can be obtained by e-mailing Luke Conway at [luke.conway@umontana.edu](mailto:luke.conway@umontana.edu).

In all cases, scales are presented with options from 1-7 anchored by “1 = not true of me at all” and (7 = “very true of me.”).

* = Reverse-scored item.

**Perceived Coronavirus Threat Questionnaire**

Thinking about the coronavirus (COVID-19) makes me feel threatened.

I am afraid of the coronavirus (COVID-19).

I am not worried about the coronavirus (COVID-19).*

I am worried that I or people I love will get sick from the coronavirus (COVID-19).

I am stressed around other people because I worry I’ll catch the coronavirus (COVID-19).

I have tried hard to avoid other people because I don’t want to get sick.

**Appendix B: Long Versions of the Scales, Cont.**

Direct access to these scales on Qualtrics can be obtained by e-mailing Luke Conway at [luke.conway@umontana.edu](mailto:luke.conway@umontana.edu).

In all cases, scales are presented with options from 1-7 anchored by “1 = not true of me at all” and (7 = “very true of me.”).

* = Reverse-scored item.

**Federal Governmental Response to Coronavirus Questionnaire**

Restriction Scale

I support Federal government measures to restrict the movement of American citizens to curb the spread of Coronavirus (COVID-19).

We need strong Federal government officials right now to take action to stop the spread of disease.

Punishment Scale

I want my Federal government to severely punish those who violate orders to stay home.

It is vital right now that the Federal government strongly punishes people who do not engage in social distancing measures.

Reactance Scale

I am upset at the thought that my Federal government would force people to stay home against their will.

It makes me angry that the Federal government would tell me where I can go and what I can do, even when there is a crisis such as Coronavirus (COVID-19).

Research Scale

I think we should spend most of our Federal resources right now towards finding a vaccine (or other medical cure) for Coronavirus (COVID-19).

I want to see more Federal research on Coronavirus (COVID-19) because I think that’s the best way to stop it.

Stimulus Scale

I think it is a good idea for the Federal government to give individual citizens money back during these difficult times to increase spending and keep business going.

I think a Federal government stimulus package during the virus spread is a good idea.

Informational Contamination Scale

I distrust the information I receive about the Coronavirus (COVID-19) from my Federal government.

I think the Federal government has an agenda that’s causing them not to give the whole story to the populace.

**Appendix B: Long Versions of the Scales, Cont.**

Direct access to these scales on Qualtrics can be obtained by e-mailing Luke Conway at [luke.conway@umontana.edu](mailto:luke.conway@umontana.edu).

In all cases, scales are presented with options from 1-7 anchored by “1 = not true of me at all” and (7 = “very true of me.”).

* = Reverse-scored item.

**State Governmental Response to Coronavirus Questionnaire**

Restriction Scale

I support State government measures to restrict the movement of American citizens to curb the spread of Coronavirus (COVID-19).

We need strong State government officials right now to take action to stop the spread of disease.

Punishment Scale

I want my State government to severely punish those who violate orders to stay home.

It is vital right now that the State government strongly punishes people who do not engage in social distancing measures.

Reactance Scale

I am upset at the thought that my State government would force people to stay home against their will.

It makes me angry that the State government would tell me where I can go and what I can do, even when there is a crisis such as Coronavirus (COVID-19).

Research Scale

I think we should spend most of our State resources right now towards finding a vaccine (or other medical cure) for Coronavirus (COVID-19).

I want to see more State research on Coronavirus (COVID-19) because I think that’s the best way to stop it.

Stimulus Scale

I think it is a good idea for the State government to give individual citizens money back during these difficult times to increase spending and keep business going.

I think a State government stimulus package during the virus spread is a good idea.

Informational Contamination Scale

I distrust the information I receive about the Coronavirus (COVID-19) from my State government.

I think the State government has an agenda that’s causing them not to give the whole story to the populace.

**Appendix B: Long Versions of the Scales, Cont.**

Direct access to these scales on Qualtrics can be obtained by e-mailing Luke Conway at [luke.conway@umontana.edu](mailto:luke.conway@umontana.edu).

In all cases, scales are presented with options from 1-7 anchored by “1 = not true of me at all” and (7 = “very true of me.”).

* = Reverse-scored item.

**City Governmental Response to Coronavirus Questionnaire**

Restriction Scale

I support City government measures to restrict the movement of American citizens to curb the spread of Coronavirus (COVID-19).

We need strong City government officials right now to take action to stop the spread of disease.

Punishment Scale

I want my City government to severely punish those who violate orders to stay home.

It is vital right now that the City government strongly punishes people who do not engage in social distancing measures.

Reactance Scale

I am upset at the thought that my City government would force people to stay home against their will.

It makes me angry that the City government would tell me where I can go and what I can do, even when there is a crisis such as Coronavirus (COVID-19).

Research Scale

I think we should spend most of our City resources right now towards finding a vaccine (or other medical cure) for Coronavirus (COVID-19).

I want to see more City research on Coronavirus (COVID-19) because I think that’s the best way to stop it.

Stimulus Scale

I think it is a good idea for the City government to give individual citizens money back during these difficult times to increase spending and keep business going.

I think a City government stimulus package during the virus spread is a good idea.

Informational Contamination Scale

I distrust the information I receive about the Coronavirus (COVID-19) from my City government.

I think the City government has an agenda that’s causing them not to give the whole story to the populace.

**Appendix B: Long Versions of the Scales, Cont.**

Direct access to these scales on Qualtrics can be obtained by e-mailing Luke Conway at [luke.conway@umontana.edu](mailto:luke.conway@umontana.edu).

In all cases, scales are presented with options from 1-7 anchored by “1 = not true of me at all” and (7 = “very true of me.”).

* = Reverse-scored item.

**Coronavirus Impacts Questionnaire**

Financial Scale

The Coronavirus (COVID-19) has impacted me negatively from a financial point of view.

I have lost job-related income due to the Coronavirus (COVID-19).

The Coronavirus (COVID-19) has NOT impacted my financial status at all.*

Resource Scale

I have had a hard time getting needed resources (food, toilet paper) due to the Coronavirus (COVID-19).

It has been difficult for me to get the things I need due to the Coronavirus (COVID-19).

The Coronavirus (COVID-19) has NOT affected my ability to get needed resources.*

Psychological Scale

I have become depressed because of the Coronavirus (COVID-19).

The Coronavirus (COVID-19) outbreak has impacted my psychological health negatively.

The Coronavirus (COVID-19) pandemic has NOT made me feel any worse than I did before.*

**Appendix B: Long Versions of the Scales, Cont.**

Direct access to these scales on Qualtrics can be obtained by e-mailing Luke Conway at [luke.conway@umontana.edu](mailto:luke.conway@umontana.edu).

In all cases, scales are presented with options from 1-7 anchored by “1 = not true of me at all” and (7 = “very true of me.”).

* = Reverse-scored item.

**Coronavirus Experiences Questionnaire**

Personal Diagnoses/Symptoms Scale

I have been diagnosed with coronavirus (COVID-19).

I have had coronavirus-like symptoms at some point in the last two months.

I have been sick with something other than the coronavirus in the last two months.

Proximity to Others Scale

I know someone who has been diagnosed with Coronavirus

(COVID-19).

I have been in close proximity with someone who has been diagnosed with coronavirus (COVID-19).

I know someone who has had coronavirus-like symptoms in the last two months.

I have been in close proximity with someone who has had coronavirus-like symptoms in the last two months.

News Scale

I watch a lot of news about the Coronavirus (COVID-19).

I purposefully try NOT to watch news on Coronavirus (COVID-19).*

I spend a huge percentage of my time trying to find updates online or on TV about Coronavirus (COVID-19).

**Appendix C: Development of Short Versions of the Scales**

Due to limited resources or participant time (or both), researchers often seek shortened versions of scales. To facilitate this goal, we here present recommended short versions of each of the scales. The alphas for the shortened scale versions can be found in Table 11. We briefly comment here on the development of these scales (complete short versions of the scales can be found in Appendix D).

**Perceived Coronavirus Threat Questionnaire**

Across all three samples, these three items consistently loaded higher on the single factor that comprises the scale – these items serve as an excellent short version of the Perceived Coronavirus Threat Questionnaire.

**Governmental Response to Coronavirus Questionnaire**

Because the Governmental Response questionnaires at each level of government contain only two items per dimension, for researchers interested in shortening their scales, we recommend using only the Federal Governmental Response to Coronavirus Questionnaire. The scales of this questionnaire are generally highly related to the parallel scales on the other questionnaires (correlations for parallel scales across levels generally in the .60 to .80 range), and the Federal Government response is likely the most subjectively pertinent to most Americans. If researchers have other goals that include other levels of government, then they can select those scales as needed; but for general use, the 12-item Federal scale is recommended.

**Coronavirus Impacts Questionnaire**

For the Impacts Questionnaire, we recommend a shortened 6-item version that drops the con-trait items. This shortened scale set shows similar internal reliability as the longer scale (see Table 11).

**Coronavirus Experiences Questionnaire**

For the Coronavirus Experiences Questionnaire scale, we recommend that researchers treat it as a series of face-valid independent measures, rather than a coherent scale (see the Factor Analysis results from Samples 1-3 and the alphas in Table 11). As a result, we have selected the best items (based on factor loadings) for each item set and included those in a shortened version in Appendix D. Researchers can then use or adapt those items as they see fit.

**Appendix D: Recommended Short Versions of the Scales**

Direct access to these scales on Qualtrics can be obtained by e-mailing Luke Conway at [luke.conway@umontana.edu](mailto:luke.conway@umontana.edu).

In all cases, scales are presented with options from 1-7 anchored by “1 = not true of me at all” and (7 = “very true of me.”).

* = Reverse-scored item.

**Perceived Coronavirus Threat Questionnaire (Short)**

Thinking about the coronavirus (COVID-19) makes me feel threatened.

I am afraid of the coronavirus (COVID-19).

I am stressed around other people because I worry I’ll catch the coronavirus (COVID-19).

**Governmental Response to Coronavirus Questionnaire (Short)**

Restriction Scale

I support Federal government measures to restrict the movement of American citizens to curb the spread of Coronavirus (COVID-19).

We need strong Federal government officials right now to take action to stop the spread of disease.

Punishment Scale

I want my Federal government to severely punish those who violate orders to stay home.

It is vital right now that the Federal government strongly punishes people who do not engage in social distancing measures.

Reactance Scale

I am upset at the thought that my Federal government would force people to stay home against their will.

It makes me angry that the Federal government would tell me where I can go and what I can do, even when there is a crisis such as Coronavirus (COVID-19).

Research Scale

I think we should spend most of our Federal resources right now towards finding a vaccine (or other medical cure) for Coronavirus (COVID-19).

I want to see more Federal research on Coronavirus (COVID-19) because I think that’s the best way to stop it.

Stimulus Scale

I think it is a good idea for the Federal government to give individual citizens money back during these difficult times to increase spending and keep business going.

I think a Federal government stimulus package during the virus spread is a good idea.

Informational Contamination Scale

I distrust the information I receive about the Coronavirus (COVID-19) from my Federal government.

I think the Federal government has an agenda that’s causing them not to give the whole story to the populace.

**Coronavirus Impacts Questionnaire (Short)**

Financial Scale

The Coronavirus (COVID-19) has impacted me negatively from a financial point of view.

I have lost job-related income due to the Coronavirus (COVID-19).

Resource Scale

I have had a hard time getting needed resources (food, toilet paper) due to the Coronavirus (COVID-19).

It has been difficult for me to get the things I need due to the Coronavirus (COVID-19).

Psychological Scale

I have become depressed because of the Coronavirus (COVID-19).

The Coronavirus (COVID-19) outbreak has impacted my psychological health negatively.

**Coronavirus Experiences Questionnaire (Short)**

Personal Diagnoses/Symptoms Scale

I have been diagnosed with coronavirus (COVID-19).

I have had coronavirus-like symptoms at some point in the last two months.

I have been sick with something other than the coronavirus in the last two months.

Proximity to Others Scale

I have been in close proximity with someone who has been diagnosed with coronavirus (COVID-19).

I have been in close proximity with someone who has had coronavirus-like symptoms in the last two months.

News Scale

I watch a lot of news about the Coronavirus (COVID-19).

I spend a huge percentage of my time trying to find updates online or on TV about Coronavirus (COVID-19).

**Samples 1-3: Factor Loadings**

Table 1: Sample 1, Perceived Coronavirus Threat Questionnaire

| Items | Component |
| --- | --- |
|  | 1 |
| Thinking about the coronavirus (COVID-19) makes me feel  threatened. | **.898** |
| I am afraid of the coronavirus (COVID-19). | **.916** |
| I am not worried about the coronavirus (COVID-19).* | **-.539** |
| I am worried that I or people I love will get sick from the  coronavirus (COVID-19). | **.865** |
| I am stressed around other people because I worry, I’ll catch  the coronavirus (COVID-19). | **.873** |
| I have tried hard to avoid other people because I don’t want to get sick. | **.795** |

*Note*. *N* = 279. Factor loadings above .40 are in bold. *Reverse-scored item.

Table 2: Sample 1, Government Responses to Coronavirus Questionnaire

| Items | Components | | | | | | |
| --- | --- | --- | --- | --- | --- | --- | --- |
|  | 1 | 2 | 3 | 4 | 5 | 6 | 7 |
| Federal Government Items: |  |  |  |  |  |  |  |
|  |  |  |  |  |  |  |  |
| I support Federal government measures to restrict the  movement of American citizens to curb the spread of Coronavirus (COVID-19). | **.763** | -.348 | .135 | -.109 | .091 | .101 | -.035 |
| We need strong Federal government officials right now to take action to stop the spread of disease. | **.788** | -.299 | .107 | -.114 | .093 | .101 | -.027 |
| I want my Federal government to severely punish those who violate orders to stay home. | **.325** | -.074 | .171 | -.011 | .097 | **.853** | -.025 |
| It is vital right now that the Federal government strongly enforces social distancing measures. | **.693** | -.284 | .253 | -.037 | .178 | .364 | -.055 |
| I am upset at the thought that my Federal government would force people to stay home against their will. | -.271 | **.796** | -.028 | .207 | -.026 | -.091 | .075 |
| It makes me angry that the Federal government would tell me where I can go and what I can do, even when there is a crisis such as Coronavirus (COVID-19). | -.290 | **.860** | -.046 | .179 | -.060 | -.048 | .020 |
| I think we should spend most of our Federal resources right now towards finding a vaccine (or other medical cure) for Coronavirus (COVID-19). | **.377** | -.089 | **.657** | .008 | .202 | .143 | .181 |
| I want to see more Federal research on Coronavirus (COVID-19) because I think that’s the best way to stop it. | **.548** | -.069 | **.607** | -.078 | .200 | .055 | .246 |
| I think it is a good idea for the Federal government to give individual citizens money back during these difficult times to increase spending and keep business going. | **.423** | -.092 | .084 | -.139 | **.697** | .021 | .302 |
| I think a Federal government stimulus package during the virus spread is a good idea. | **.494** | -.128 | .079 | -.149 | **.678** | -.025 | .171 |
| I distrust the information I receive about the Coronavirus (COVID-19) from my Federal government. | .011 | .067 | .021 | **.629** | .009 | -.018 | **.534** |
| I think the Federal government has an agenda that’s causing them not to give the whole story to the populace. | -.056 | .046 | .025 | **.672** | .076 | .078 | **.564** |

| State Government Items |  |  |  |  |  |  |  |
| --- | --- | --- | --- | --- | --- | --- | --- |
|  |  |  |  |  |  |  |  |
| We need strong State government officials right now to take action to stop the spread of disease. | **.749** | -.349 | .098 | -.153 | .203 | .181 | .071 |
| I want my State government to severely punish those who violate orders to stay home. | .299 | -.127 | .180 | -.011 | .137 | **.876** | .030 |
| It is vital right now that the State government strongly enforces social distancing measures. | **.673** | -.254 | .229 | -.089 | .204 | **.366** | .070 |
| I am upset at the thought that my State government would force people to stay home against their will. | -.322 | **.818** | -.081 | .238 | -.087 | -.137 | -.039 |
| It makes me angry that the State government would tell me where I can go and what I can do, even when there is a crisis such as Coronavirus (COVID-19). | -.277 | **.838** | -.046 | .198 | -.037 | -.034 | .027 |
| I think we should spend most of our State resources right now towards finding a vaccine (or other medical cure) for Coronavirus (COVID-19). | .135 | -.124 | **.848** | .031 | .234 | .162 | .076 |
| I want to see more State research on Coronavirus (COVID-19) because I think that’s the best way to stop it. | **.326** | -.130 | **.770** | .021 | .251 | .131 | .104 |
| I think it is a good idea for the State government to give individual citizens money back during these difficult times to increase spending and keep business going. | .158 | -.134 | .322 | -.013 | **.801** | .164 | .082 |
| I think a State government stimulus package during the virus spread is a good idea. | .182 | -.094 | **.303** | .015 | **.828** | .151 | .041 |
| I distrust the information I receive about the Coronavirus (COVID-19) from my State government. | -.126 | .191 | .022 | **.841** | -.073 | -.096 | .009 |
| I think the State government has an agenda that’s causing them not to give the whole story to the populace. | -.114 | .230 | .027 | **.864** | -.005 | -.002 | -.008 |
|  |  |  |  |  |  |  |  |
| City Government Items: |  |  |  |  |  |  |  |
|  |  |  |  |  |  |  |  |
| I support City government measures to restrict the  movement of American citizens to curb the spread of Coronavirus (COVID-19). | **.729** | -.302 | .223 | -.023 | .166 | .173 | -.104 |
| We need strong City government officials right now to  take action to stop the spread of disease. | **.754** | -.285 | .179 | -.059 | .181 | .246 | -.105 |
| I want my City government to severely punish those who violate orders to stay home. | .282 | -.109 | .194 | .024 | .089 | **.861** | -.019 |
| It is vital right now that the City government strongly enforces social distancing measures. | **.687** | -.251 | .286 | -.036 | .196 | **.374** | -.016 |
| I am upset at the thought that my City government would force people to stay home against their will. | -.291 | **.835** | -.071 | .210 | -.074 | -.111 | -.056 |
| It makes me angry that the City government would tell me where I can go and what I can do, even when there is a crisis such as Coronavirus (COVID-19). | -.323 | **.832** | -.076 | .201 | -.053 | -.057 | -.029 |
| I think we should spend most of our City resources right now towards finding a vaccine (or other medical cure) for Coronavirus (COVID-19). | .078 | -.018 | **.803** | .183 | .252 | .159 | -.303 |
| I want to see more City research on Coronavirus (COVID-19) because I think that’s the best way to stop it. | .180 | .055 | **.759** | .175 | .257 | .171 | -.342 |
| I think it is a good idea for the City government to give individual citizens money back during these difficult times to increase spending and keep business going. | .103 | .026 | **.345** | .140 | **.699** | .100 | -.407 |
| I think a City government stimulus package during the  virus spread is a good idea. | .105 | .043 | **.327** | .111 | **.753** | .089 | -.367 |
| I distrust the information I receive about the Coronavirus (COVID-19) from my City government. | -.125 | .249 | .076 | **.836** | .020 | -.025 | -.108 |
| I think the City government has an agenda that’s causing them not to give the whole story to the populace. | -.075 | **.326** | .133 | **.790** | .014 | .089 | -.072 |

*Note*. *N* = 279. Factor loadings above .40 are in bold.

Table 3: Sample 1, COVID Experiences Questionnaire

| Items | Components | | | | |
| --- | --- | --- | --- | --- | --- |
|  | 1 | 2 | 3 | 4 | 5 |
| I have been diagnosed with coronavirus (COVID-19). | .146 | .016 | **.769** | .096 | .170 |
| I have had coronavirus-like symptoms at some point in the last two months. | **.773** | -.006 | .290 | .160 | .068 |
| I have been sick with something other than the coronavirus in the last two months. | **.628** | .198 | -.036 | -.004 | -.015 |
| The Coronavirus (COVID-19) has impacted me negatively from a financial point of view. | .121 | .244 | -.033 | **.856** | -.059 |
| I have lost job-related income due to the Coronavirus (COVID-19). | .074 | .130 | .192 | **.865** | .010 |
| I have had a hard time getting needed resources (food, toilet paper) due to the Coronavirus (COVID-19). | .077 | **.593** | -.043 | .368 | -.044 |
| I have become depressed because of the Coronavirus (COVID-19). | .123 | **.902** | .173 | .096 | -.019 |
| The Coronavirus (COVID-19) outbreak has impacted my psychological health negatively. | .169 | **.899** | .122 | .120 | -.055 |
| I know someone who has been diagnosed with Coronavirus (COVID-19). | .297 | .176 | **.705** | -.030 | -.104 |
| I have been in close proximity with someone who has been diagnosed with coronavirus (COVID-19). | .230 | .079 | **.852** | .091 | .096 |
| I know someone who has had coronavirus-like symptoms in the  last two months. | **.795** | .116 | .331 | .044 | -.031 |
| I have been in close proximity with someone who has had coronavirus-like symptoms in the last two months. | **.825** | .076 | .297 | .118 | .040 |
| I watch a lot of news about the Coronavirus (COVID-19).* | .034 | .268 | .066 | .074 | **-.860** |
| I purposefully try NOT to watch news on Coronavirus (COVID-19). | .070 | .151 | .231 | .017 | **.854** |

*Note*. *N* = 279. Factor loadings above .40 are in bold. *Reverse-scored item.

Table 4: Sample 2, Federal Government Responses to Coronavirus Questionnaire

| Items |  | Components | | | | |  |
| --- | --- | --- | --- | --- | --- | --- | --- |
|  | 1 | | 2 | 3 | 4 | 5 | 6 |
| I support Federal government measures to restrict the movement of American citizens to curb the spread of Coronavirus (COVID-19). | .200 | | -.324 | .171 | .179 | -.124 | **.805** |
| We need strong Federal government officials right now to take action to stop the spread of disease. | .215 | | -.316 | .207 | .179 | -.134 | **.787** |
| I want my Federal government to severely punish those who violate orders to stay home. | **.948** | | -.095 | .077 | .133 | -.045 | .149 |
| It is vital right now that the Federal government strongly punishes people who do not engage in social distancing measures. | **.943** | | -.087 | .028 | .161 | .001 | .163 |
| I am upset at the thought that my Federal government would force people to stay home against their will. | -.147 | | **.898** | -.104 | -.046 | .128 | -.252 |
| It makes me angry that the Federal government would tell me where I can go and what I can do, even when there is a crisis such as Coronavirus (COVID-19). | -.043 | | **.859** | -.146 | -.135 | .228 | -.277 |
| I think we should spend most of our Federal resources right now towards finding a vaccine (or other medical cure) for Coronavirus (COVID-19). | .135 | | -.055 | .081 | **.903** | .025 | .137 |
| I want to see more Federal research on Coronavirus (COVID-19) because I think that’s the best way to stop it. | .154 | | -.108 | .224 | **.860** | .009 | .132 |
| I think it is a good idea for the Federal government to give individual citizens money back during these difficult times to increase spending and keep business going. | .063 | | -.084 | **.907** | .176 | .018 | .087 |
| I think a Federal government stimulus package during the virus spread is a good idea. | .038 | | -.137 | **.889** | .112 | .003 | .198 |
| I distrust the information I receive about the Coronavirus (COVID-19) from my Federal government. | -.098 | | .170 | -.095 | -.018 | **.898** | -.053 |
| I think the Federal government has an agenda that’s causing them not to give the whole story to the populace. | .055 | | .118 | .117 | .053 | **.893** | -.135 |

*Note*. *N* = 285. Factor loadings above .40 are in bold.

Table 5: Sample 2, State Government Responses to Coronavirus Questionnaire

| Items | Components | | | | | |
| --- | --- | --- | --- | --- | --- | --- |
|  | 1 | 2 | 3 | 4 | 5 | 6 |
| I support State government measures to restrict the movement of American citizens to curb the spread of Coronavirus (COVID-19). | .140 | .169 | -.375 | -.219 | .111 | **.821** |
| We need strong State government officials right now to take action to stop the spread of disease. | .207 | .079 | -.291 | -.214 | .105 | **.865** |
| I want my State government to severely punish those who violate orders to stay home. | **.952** | .060 | -.091 | -.010 | .121 | .148 |
| It is vital right now that the State government strongly punishes people who do not engage in social distancing measures. | **.949** | .078 | -.124 | -.023 | .126 | .122 |
| I am upset at the thought that my State government would force people to stay home against their will. | -.155 | -.065 | **.861** | .278 | -.066 | -.289 |
| It makes me angry that the State government would tell me where I can go and what I can do, even when there is a crisis such as Coronavirus (COVID-19). | -.104 | -.010 | **.862** | .267 | -.054 | -.326 |
| I think we should spend most of our State resources right now towards finding a vaccine (or other medical cure) for Coronavirus (COVID-19). | .146 | .186 | -.064 | .052 | **.921** | .086 |
| I want to see more State research on Coronavirus (COVID-19) because I think that’s the best way to stop it. | .109 | .293 | -.040 | .057 | **.895** | .084 |
| I think it is a good idea for the State government to give individual citizens money back during these difficult times to increase spending and keep business going. | .088 | **.927** | -.036 | .000 | .218 | .065 |
| I think a State government stimulus package during the virus spread is a good idea. | .049 | **.918** | -.033 | .051 | .235 | .119 |
| I distrust the information I receive about the Coronavirus (COVID-19) from my State government. | -.069 | .000 | .259 | **.863** | .058 | -.230 |
| I think the State government has an agenda that’s causing them not to give the whole story to the populace. | .035 | .055 | .198 | **.913** | .058 | -.125 |

*Note*. *N* = 285. Factor loadings above .40 are in bold.

Table 6: Sample 2, City Government Responses to Coronavirus Questionnaire

| Items | Components | | | | | |
| --- | --- | --- | --- | --- | --- | --- |
|  | 1 | 2 | 3 | 4 | 5 | 6 |
| I support City government measures to restrict the movement of American citizens to curb the spread of Coronavirus (COVID-19). | .161 | .067 | -.338 | -.235 | .093 | **.826** |
| We need strong City government officials right now to take action to stop the spread of disease. | .244 | .098 | -.295 | -.153 | .067 | **.846** |
| I want my City government to severely punish those who violate orders to stay home. | **.957** | .087 | -.099 | .011 | .112 | .155 |
| It is vital right now that the City government strongly punishes people who do not engage in social distancing measures. | **.947** | .085 | -.115 | .014 | .143 | .176 |
| I am upset at the thought that my City government would force people to stay home against their will. | -.135 | .028 | **.908** | .200 | .021 | -.269 |
| It makes me angry that the City government would tell me where I can go and what I can do, even when there is a crisis such as Coronavirus (COVID-19). | -.099 | .009 | **.884** | .244 | .056 | -.309 |
| I think we should spend most of our City resources right now towards finding a vaccine (or other medical cure) for Coronavirus (COVID-19). | .163 | .312 | .024 | .135 | **.902** | .091 |
| I want to see more City research on Coronavirus (COVID-19) because I think that’s the best way to stop it. | .134 | .417 | .048 | .087 | **.866** | .053 |
| I think it is a good idea for the City government to give individual citizens money back during these difficult times to increase spending and keep business going. | .087 | **.918** | -.011 | .069 | .281 | .067 |
| I think a City government stimulus package during the virus spread is a good idea. | .087 | **.883** | .037 | .084 | .353 | .075 |
| I distrust the information I receive about the Coronavirus (COVID-19) from my City government. | -.013 | .040 | .232 | **.907** | .080 | -.157 |
| I think the City government has an agenda that’s causing them not to give the whole story to the populace. | .041 | .113 | .163 | **.911** | .116 | -.162 |

*Note*. *N* = 285. Factor loadings above .40 are in bold.

Table 7: Sample 2, COVID Impacts Questionnaire

| Items | Components | | |
| --- | --- | --- | --- |
|  | 1 | 2 | 3 |
| The Coronavirus (COVID-19) has impacted me negatively from a financial point of view. | .157 | .192 | **.883** |
| I have lost job-related income due to the Coronavirus (COVID-19). | .151 | .112 | **.783** |
| The Coronavirus (COVID-19) has NOT impacted my financial status at all.* | .006 | -.066 | **-.900** |
| I have had a hard time getting needed resources (food, toilet paper) due to the Coronavirus (COVID-19). | .159 | **.924** | .048 |
| It has been difficult for me to get the things I need due to the Coronavirus (COVID-19). | .179 | **.917** | .096 |
| The Coronavirus (COVID-19) has NOT affected my ability to get needed resources.* | -.044 | **-.752** | -.225 |
| I have become depressed because of the Coronavirus (COVID-19). | **.912** | .100 | .060 |
| The Coronavirus (COVID-19) outbreak has impacted my psychological health negatively. | **.927** | .137 | .096 |
| The Coronavirus (COVID-19) pandemic has NOT made me feel any worse than I did before.* | **-.770** | -.126 | -.133 |

*Note*. *N* = 285. Factor loadings above .40 are in bold. *Reverse-scored item.

Table 8: Sample 2, COVID Experiences Questionnaire

| Items | Components | | | |
| --- | --- | --- | --- | --- |
|  | 1 | 2 | 3 | 4 |
| I have been diagnosed with coronavirus (COVID-19). | **.411** | **.587** | -.212 | .259 |
| I have had coronavirus-like symptoms at some point in the last two months. | .176 | **.809** | .046 | -.251 |
| I have been sick with something other than the coronavirus in the last two months. | .079 | **.749** | .033 | -.150 |
| I know someone who has been diagnosed with Coronavirus  (COVID-19). | **.878** | -.001 | .092 | -.023 |
| I have been in close proximity with someone who has been  diagnosed with coronavirus (COVID-19). | **.758** | .311 | -.034 | -.170 |
| I have been NOT been anywhere close to someone who has been diagnosed with coronavirus (COVID-19).* | -.243 | .003 | .186 | **.696** |
| I know someone who has had coronavirus-like symptoms in the  last two months. | **.688** | .203 | .007 | -.374 |
| I have been in close proximity with someone who has had  coronavirus-like symptoms in the last two months. | **.549** | **.411** | .054 | -.483 |
| I am confident that I have NOT been anywhere close to someone who has had  coronavirus-like symptoms in the last two months.* | -.077 | -.235 | -.026 | **.822** |
| I watch a lot of news about the Coronavirus (COVID-19). | .015 | .022 | **.874** | .093 |
| I purposefully try NOT to watch news on Coronavirus (COVID-19).* | .135 | .211 | **-.771** | .136 |
| I spend a huge percentage of my time trying to find updates online or on TV about Coronavirus (COVID-19). | .245 | .229 | **.734** | .219 |

*Note*. *N* = 285. Factor loadings above .40 are in bold. *Reverse-scored item.

Table 9: Sample 3, COVID Impacts Questionnaire

| Items | Components | | |
| --- | --- | --- | --- |
|  | 1 | 2 | 3 |
| The Coronavirus (COVID-19) has impacted me negatively from a financial point of view. | .163 | .219 | .**858** |
| I have lost job-related income due to the Coronavirus (COVID-19). | .121 | .096 | **.799** |
| The Coronavirus (COVID-19) has NOT impacted my financial status at all.* | -.105 | -.024 | **-.852** |
| I have had a hard time getting needed resources (food, toilet paper) due to the Coronavirus (COVID-19). | **.908** | .164 | .075 |
| It has been difficult for me to get the things I need due to the Coronavirus (COVID-19). | **.893** | .191 | .167 |
| The Coronavirus (COVID-19) has NOT affected my ability to get needed resources.* | **-.785** | -.061 | -.157 |
| I have become depressed because of the Coronavirus (COVID-19). | .066 | **.888** | .116 |
| The Coronavirus (COVID-19) outbreak has impacted my psychological health negatively. | .133 | **.926** | .117 |
| The Coronavirus (COVID-19) pandemic has NOT made me feel any worse than I did before.* | -.181 | **-.700** | -.082 |

*Note*. *N* = 413. Factor loadings above .40 are in bold. *Reverse-scored item.

Table 10: Sample 3, COVID Experiences Questionnaire

| Items | Components | | |
| --- | --- | --- | --- |
|  | 1 | 2 | 3 |
| I have been diagnosed with coronavirus (COVID-19). | .133 | **.796** | -.095 |
| I have had coronavirus-like symptoms at some point in the last two months. | **.805** | .255 | .028 |
| I have been sick with something other than the coronavirus in the last two months. | **.750** | .053 | -.016 |
| I know someone who has been diagnosed with Coronavirus  (COVID-19). | .282 | .**612** | .036 |
| I have been in close proximity with someone who has been diagnosed with coronavirus (COVID-19). | .341 | **.767** | .053 |
| I know someone who has had coronavirus-like symptoms in the last two months. | **.751** | .270 | -.003 |
| I have been in close proximity with someone who has had coronavirus-like symptoms in the last two months. | **.781** | .322 | .012 |
| I watch a lot of news about the Coronavirus (COVID-19). | .051 | -.017 | **.854** |
| I purposefully try NOT to watch news on Coronavirus (COVID-19).*** | .037 | .261 | **-.702** |
| I spend a huge percentage of my time trying to find updates online or on TV about Coronavirus (COVID-19). | -.003 | .274 | **.732** |

*Note*. *N* = 413. Factor loadings above .40 are in bold. *Reverse-scored item.

**Primary Investigation: Additional Information About Each Sample**

**[BLINDED FOR REVIEW]**

**Participants**

The study involved 332 adults (76.2% females; males: 23.8%) aged between 19 and 73 years (M =30.18; SD = 12.94) and coming from Northern Italy (98,2%). More than half of participants (54.8%) had a high school degree, 25% a bachelor’s degree, 13.6% a master’s degree, 3.6% a middle school degree and 3% obtained a PhD or post-graduate school degree. Most participants (39.5%) were full-time students, 26.8% were full-time employed, 16.3% were part-time students having occasional or part-time jobs, and 7.5% were part-time employed.

Participants were mainly unmarried (78.6%) and had a moderately liberal political orientation (M=4.5; SD=1.95).

**Procedure**

Participants were contacted through social networks, messaging platforms, and university pages, and invited to complete an anonymous questionnaire. The invitation message indicated that the purpose of the study was to examine the effects of COVID-19 outbreak on personal well-being and that participants had to be Italian and at least 18 years old.

Data were collected from April 27 to May 10 2020 during the so called “lockdown” period, when mandatory restrictive measures, initially implemented in specific northern Italy areas, where applied uniformly throughout the whole country. These measures mandated staying at home and leaving it only if groceries, medical supplies or treatment were needed, attending school and university activities were only available remotely, shutting business (except for food, healthcare, and IT industries) or working from home, avoid physical proximity to non-cohabiting others. During the data collection period over 21,000 Italians were diagnosed with COVID-19 and 3,900 died after being infected.

**Measures**

Participants completed the following Questionnaires developed by Conway, Woodard and Zubrod (2020).

*Short version of Perceived Coronavirus Threat Questionnaire*. Participants rated their degree of agreement with each item on a 7-point Likert-type scale ranging from 1 (definitely disagree) to 7 (definitely agree)(α = .85).

*Short version of Coronavirus Impacts Questionnaire*. Participants rated their degree of agreement with each item on a 7-point Likert-type scale ranging from 1 (definitely disagree) to 7 (definitely agree) (αs = .81, 71, and .83 for the Financial, the Resource, and the Psychological scale respectively).

*Short version of Coronavirus Experiences Questionnaire.* The response format for the Personal diagnoses/symptoms and the Proximity to other scales was in the present study dichotomous (0=no; 1 =yes), whereas it was a 7-point Likert-type scale ranging from 1 (definitely disagree) to 7 (definitely agree) for the News scale (α = .76).

Participant were asked to indicate their degree of agreement or disagreement with each of the previous questionnaires items.

The data were collected as part of a study aimed at analysing to what extent COVID-19 related stigma, threats and experiences were related to the psychosocial well-being of the subjects living in one of the European districts more severely stricken by the virus during the nationwide lockdown period, thereby analyzing the subjects' perceptions in a particularly challenging situation.

**[BLINDED FOR REVIEW]**

**COVID-19 IN TURKEY**

The total number of tests performed in Turkey from 11 March 2020, when the first coronavirus patient was reported, to 6 December 2020, is 19.691.845. The total number of patients is 539.291 and the total number of deaths is 14.900 (Republic of Turkey Ministry of Health, 2020a). Considering the number of patients by the date of 25/10/2020 in Turkey, the highest number of patients is in Istanbul. (140.192 people). The highest number of patients on the basis of the region are respectively in West Anatolia, Southeast Anatolia, East Marmara and Aegean Region (Republic of Turkey Ministry of Health, 2020b).

Following the detection of the first case on March 11, with the circular sent by the Ministry of Interior to 81 provincial governorships, 149,382 workplaces across the country temporarily suspended their activities between 15-18 March (Ministry of the Interior, 2020). With the Presidential Circular dated 22 March 2020; It has been stated that flexible working methods such as remote working and rotational work can be applied to those working in public institutions and organizations (Turkey - Legal Gazette, 2020). School closures as of 12 March, playing sports competitions without spectators, closing bars, nightclubs, theaters, cinemas, gyms and cafes, ending collective praying in mosques, two-week quarantine obligation for everyone returning from abroad, declaring all private hospitals as pandemic hospitals, postponement of cultural and scientific activities, suspension of flights with most countries, curfews from time to time, curfews without masks and intercity transportation restrictions were taken to control the epidemic (BBC, 2020). As of June 1, the normalization process has been started with the opening of restaurants and cafes, allowing some domestic flights and lifting restrictions on intercity transportation. Turkey, as in most countries throughout the world, by taking some precautions and measures, has tried to minimize the damage of such a large-scale epidemic.

**Method and Participants**

All study procedures were approved by the [BLINDED FOR REVIEW]. In this research, for the purpose of social psychological measurement of Covid-19, the short form of Perceived Coronavirus Threat, Governmental Response to Coronavirus, Coronavirus Impacts, Coronavirus Experiences Questionnaires developed by Conway et al. (2020), was used. In addition to Covid-19 scales; Demographic questions were asked about gender, age, marital status, education level, income level, place of residence and whether there is a chronic disease. The participants of the study consisted of the participants over the age of 18 residing in Turkey. In the first stage, the original form of the scale was translated into Turkish by experts in their field. Later, the online questionnaire was delivered to the participants through the personal relations of the authors, social media platforms, and the automation information system of the university to which the authors belong. The data were collected from 1879 people between 11.06.2020-21.09.2020. There are students, housewives, unemployed, civil servants, teachers, academicians, doctors, nurses, factory workers, business managers, soldiers, and individuals with different education levels and ages among the 1879 people whose data are collected; Most of the participants are individuals living in provinces such as Ankara, Konya, and Bursa where the number of cases is high. Participants' information is as follows:

• While 807 (42.9%) of the 1879 participants were female, 1072 (57.1%) were male.

• Looking at the age distribution, 33.1% of the participants are in the 18-25 age range, 23.2%, 26-33; 17.7%, 34-41; 13.9%, 42-49; 8.8%, 50-57; 2.9% 58-65; 0.5% of them are between the ages of 66-73 and one participant is 74 and over.

• 48.7% of the participants are single while 51.3% are married.

• Thirteen participants were literate but did not graduate from any school. Six participants have primary school degrees, 8 participants have secondary school degrees, 253 participants have high school degrees, 1053 participants have university degrees, 308 participants have master degrees, and 238 participants have a doctorate degree.

• While 9.4% of the participants have an income below the minimum wage, 18% have an income more than the minimum wage, 37% twice the minimum wage, and 35.6% have an income more than three times the minimum wage.

• 61% of the participants are in metropolitan cities, 24% is in cities, 12.5% ​​reside in the districts and 2.5% in the village.

• In addition, the participants were asked whether they have a chronic disease or not. 16.7% of the participants stated that they have a chronic disease and 83.3% stated that they do not have a chronic disease.

**Ethics Committee Approval Date: 09.06.2020**

**[BLINDED FOR REVIEW]**

**Survey sites were the USA and Poland, but residents ages 18+ of any country were invited to participate. The survey in English and Polish was administered online (using Qualtrics) and was fielded in June-July 2020. Participants were recruited via email and social media using the researcher’s professional and personal networks in the US and Poland. Anyone aged 18+ who received the survey link was invited to participate. The following scales were included: Perceived coronavirus threat (3 items), Federal/national government response to coronavirus (11 items), Coronavirus impacts (5 items), and Coronavirus experiences (6 items). Standard demographics and additional measures were also collected. The project examined a) COVID-19 attitudes and practices in relation to ideologies (political, religion) (Szaflarski 2020, forthcoming) and b) health/health care related issues, incl. health care workers’ experiences and perspectives (subsample).**

**[BLINDED FOR REVIEW]**

**Sample**

The study included 383 participants, of which 288 completed the questions and questionnaires until the main task and 239 subjects completed the entire study including the main task and the last questionnaires (Massaccesi et al., 2021). All participants were older than 18 years and provided consent to the use of the collected anonymous data. Participants were recruited via advertisements posted on social media (e.g. Facebook) and via direct contact (e.g. email). Participation was voluntary, and participants were not given any incentive for their participation. The study was approved by the [BLINDED FOR REVIEW].

**Procedure**

Before starting the online study, participants could choose their preferred language to conduct the experiment in English, Italian or German. After reading the instructions and providing consent, participants provided demographic data (e.g., age, gender, nationality) and answered a series of questions related to their personal situation during the COVID-19 pandemic (e.g. whether they were in social isolation, if their way to communicate with close others had changed, etc.). Participants also filled out the “Perceived Coronavirus Threat” and the “Coronavirus Impacts” questionnaires (Conway et al., 2020). Then, they engaged in an online experiment in which they had to judge 60 pictures depicting a single person, small or large groups of people in terms of valence, arousal and perceived physical distance. Lastly, participants filled out the UCLA Loneliness Scale (Russell, 1996), the Lubben Social Network Scale (LSNS; Lubben et al., 2006), the Positive and Negative Affect Scale (PANAS; Watson et al., 1988), and the 10-item Big Five Inventory (Rammstedt & John, 2007). The total duration of the study was approximately 25 minutes, and data were collected from 30 of April 2020 to 15 of May 2020, via the online platform SosciSurvey (www.soscisurvey.de).

**[BLINDED FOR REVIEW]**

**Participants and procedure**

A total of 155 participants in Alaska’s commercial seafood industry were recruited via advertisements on social media (e.g., Facebook and Twitter). Participation was voluntary and participants were not given any incentive for their participation. The parent study received ethical approval from the Ethics Review Board of the [BLINDED FOR REVIEW] prior to the study. After participants were informed of the aim of the study, the confidentiality of the study and provided consent, participants provided demographic data (e.g., age, gender, ethnicity) plus tenure, job title.

**Measures**

Personality was measured with the Ten Item Personality Measure (TIPI) (Gosling, Rentfrow, & Swann, 2003, Positive and negative affect were measured using the Positive and Negative Affect Schedule (PANAS) of Watson, Clark & Tellegen (1988), Turnover intention was measured by the three-item Turnover Intention Scale of De Gieter, Hofmans and Pepermans (2011), Coronavirus Anxiety was measured by the Coronavirus anxiety scale (Lee, 2020), Optimism was measured (Scheier et al., 1994), Subject wellbeing (Diener et al., 2011) and finally

Covid-19 Concern, Experience subscales were used as well as one item each and three items from the Government subscale were measured (Conway et al., 2020). Data were collected from May 19^th^ to July 17^th^, 2020 via the online platform Qualtrics ([www.qualtrics.com](http://www.qualtrics.com)).

**[BLINDED FOR REVIEW]**

**Methods**

All study procedures were approved by the [BLINDED FOR REVIEW]. We administered the long versions of the Perceived Coronavirus Threat Questionnaire and the Governmental Response to Coronavirus Questionnaire^[[3]](#footnote-3)^ as part of a larger project examining the psychological impacts of COVID-19 and social distancing measures. This project consisted of an intake survey, diary entries completed three times a week for four weeks, and an exit survey administered approximately one month after the intake survey. The Perceived Coronavirus Threat Questionnaire and the Governmental Response to Coronavirus Questionnaire were administered during the intake survey.

Participants were recruited via the CloudResearch (formerly TurkPrime) participant-sourcing platform for online research. The study was made available to participants residing in Canada and the United States who had an approval rating of 95%+ on the platform (only Americans ended up participating in the survey). We recruited 540 participants for the intake survey between June 26 and July 8, 2020. After review of the data, 22 participants were flagged for implausibly short response times (i.e., these participants took 15 minutes or less to complete what was approximately a 45 minute survey), and an additional 158 participants were flagged for nonsensical/suspicious responses on open-ended questions on the survey. Thus, the final sample consisted of 360 participants. The intake survey took approximately 45 minutes and participants were compensated $2.25.

**[BLINDED FOR REVIEW]**

**:**

We recruited a combination of local and national participants who had previously participated in a fully remote randomized controlled trial testing a mobile health app. All participants who completed baseline measures in the previous trial (approximately n = 331) were invited to complete a follow-up assessment that included the Conway COVID-19 items. No directions were given to participants beyond the items themselves. 154 participants completed the follow-up assessment. We administered the perceived threat, coronavirus impact, and coronavirus experiences questionnaires. Participants completed the COVID-19 questionnaires between May 4 and 17, 2020.

**[BLINDED FOR REVIEW]**

The parent study received ethical approval from the [BLINDED FOR REVIEW] prior to commencement. Questionnaires were provided to a sample of 315 graduate and undergraduate participants from universities and research centers in northwest Mexico (271 completed the study). The longitudinal study included a translated and back-translated Spanish version of the “perceived threat subscale”. The primary objective of the parent study was to examine the predictive nature of personality and stress on mental health over a two-semester period. A total of four waves were completed once at the beginning of the semester and again at the end of the semester. The perceived threat subscale was included in the fourth and final wave, during a state-imposed quarantine due to the COVID-19 pandemic outbreak (April 20 to May 15, 2020). Participants were recruited through flyers posted around the campus as well as via social media, professor invitations, and snowball effect. For the fourth wave, participants were provided with a link to a Qualtrics online survey, which had the additional advantage of allowing for data collection during quarantine and while the universities and research centers were closed to promote social distancing. Each participant was informed of the objectives of the study and digitally signed an informed consent form prior to participation. Participants were compensated for their time a total of 400 Mexican pesos (approximately $20 USD, half following the second wave and half upon completion of the fourth wave). The student sample was largely female (70.5%) with 65.7% having completed at least high school, 28% having completed undergraduate and 6.3% having completed a graduate degree. Over half (55.3%) of the sample were psychology undergraduate or graduate students (52% and 3.3% respectively). The remaining students covered a wide range of majors from 15 additional degree programs including medicine/nursing/dentistry (10%), engineering (8.5%), law (5.2%), and administration/accounting (2.6%). Income was also diverse with 7.7% earning a household income of less than $4,000 pesos per month (approx. $180 USD), 26.9% between $4,000 and $9,000 pesos per month, 31.7% between $9,000 and $20,000 pesos per month, 26.2% between $20,000 and $40,000 pesos per month, 6.3% between $40,000 and $85,000 pesos per month, and 1.1% earning $85,000 pesos or more per month.

**[BLINDED FOR REVIEW]**

**Method and Participants**

The English versions of the Coronavirus Perceived Threat, Government Response, Impacts, and Experiences Questionnaires (Conway, Woodard, & Zubrod, 2020) were translated into the Polish language by Karolina Grotkowski and Aneta Przepiorka. The Perceived Threat, Impacts, and Experiences Questionnaires were readily translated and adopted into Polish. However, there is no separation of city, state, and federal government in Poland as there is in the United States and thus only the Federal Government Response questionnaire was translated.

The data were collected as part of a larger project focusing on translating and adopting the Negative Mood Regulation Expectancies scale (NMRE; Catanzaro & Mearns, 1990) for use in the Polish population. The web-based crowdsourcing platform, Ariadna (Ogolnopolski Panel Badawczy Ariadna see <https://panelariadna.pl>) was used to collect data from participants living in Poland. Ariadna sends emails to registered “panelists” to determine their interest in a particular study given the length and possible reward points. Of note, Ariadna does not allow participants to skip questions. Potential participants read a brief explanation of the study and if interested, accessed the study through a link. Once the link is accessed, participants completed the informed consent process. Participants were rewarded after completing the survey. The survey began with demographic questions including age, biological sex, religion, and primary language. Individuals were disqualified from the study if they were under 18 years old. Participants completed the Polish language versions of the Coronavirus Perceived Threat, Government Response, Impacts, and Experiences Questionnaires. Participants were provided with the following instructions:

*Poniżej zadamy kilka pytań związanych z koronawirusem (COVID-19). Proszę zaznacz na ile zgadzasz się podanymi poniżej twierdzeniami (1 = „Całkowicie się nie zgadzam i 7 = „Zgadzam się całkowicie”)*

When translated back to English these instructions read:

*Below we're going to ask you several questions relevant to the Coronavirus (COVID-19).  Please respond with the number that best represents your agreement with the following statements (1 = "I definitely do not agree" and 7 = "I definitely agree.")*

Participants may have chosen to discontinue the survey at any time. Participants were compensated for completing the survey through a research grant awarded by the National Science Centre in Poland (PI: Przepiórka; Grant # 2015/19/D/HS6/01690). The Ethics Committee at the [BLINDED FOR REVIEW] has approved this study.

All data was collected during June of 2020. A total of 443 participants completed the survey. The sample was fairly split between females (n = 242, 54.6%) and males (n = 201, 45.4%). The vast majority of participants identified “Christian Catholic” as their religion (n = 377, 85.1%), followed by “other” (n = 38, 8.6%). Most of the sample (n = 218, 49.2%) stated they were living in a city with a population of under 200,000 individuals.

**Translated Scales**

*Poniżej zadamy kilka pytań związanych z koronawirusem (COVID-19). Proszę zaznacz na ile zgadzasz się podanymi poniżej twierdzeniami (1 = „Całkowicie się nie zgadzam i 7 = „Zgadzam się całkowicie”)*

*Kwestionariusz: Postrzegane zagrożenie koronawirusem*

1. Myślenie o koronawirusie (COVID-19) sprawia, że czuję się zagrożony/a.
2. Obawiam się koronawirusa (COVID-19).
3. Nie martwię się o koronawirus (COVID-19).*
4. Martwię się, że ja, lub ludzie których kocham, zachorują na koronawirus (COVID-19).
5. Stresuję się, jeżeli jestem w pobliżu innych ludzi, ponieważ obawiam się zarażenia koronawirusem (COVID-19).
6. Starałem/am się unikać innych ludzi, ponieważ nie chcę chorować.

*Odpowiedź rządu na koronawirusa kwestionariusz*

Skala Ograniczeń

1. Popieram zalecenia rządowe mające na celu zakaz przemieszczania się obywateli polskich, aby ograniczyć rozprzestrzeniania się koronawirusa (COVID-19).
2. Potrzebujemy teraz zdecydowanego działania organów rządowych, aby podjąć działania mające na celu powstrzymanie rozprzestrzeniania się choroby.

Skala kar

1. Chcę, aby mój rząd surowo ukarał tych, którzy nie przestrzegają poleceń pozostania w domu.
2. W tej chwili niezwykle ważne jest, aby rząd surowo karał ludzi, którzy nie przestrzegają zasad utrzymania dystansu społecznego.

Skala Reaktywności

1. Jestem zdenerwowany/a myślą, że rząd zmusza obywateli do pozostania w domu wbrew ich woli.
2. Jestem zły, gdy rząd nakazuje mi, gdzie mogę iść i co mogę zrobić, nawet w przypadku kryzysu takiego jak koronawirus (COVID-19).

Skala Badań

1. Myślę, że powinniśmy teraz wydać większość naszych zasobów rządowych na znalezienie szczepionki (lub innego lekarstwa) na koronawirusa (COVID-19).
2. Oczekuję zwiększenia liczby badań nad koronawirusem (COVID-19), ponieważ uważam, że to najlepszy sposób, aby go powstrzymać.

Skala pakietu stymulacyjnego

1. Myślę, że dobrym pomysłem jest, aby rząd wspomógł finansowo obywateli w tych trudnych czasach, aby zwiększyć wydatki i utrzymać biznes.
2. Myślę, że pakiet stymulacyjny zawarty w tarczy antykryzysowej (pakiet rządu podczas rozprzestrzeniania się wirusa) jest dobrym pomysłem.

Skala Zanieczyszczenie informacyjne

1. Nie ufam otrzymanym od mojego rządu informacjom na temat koronawirusa (COVID-19).
2. Myślę, że rząd ma plan, który powoduje, że nie przekazują ludziom wszystkich informacji.

*Kwestionariusz wpływów koronawirusa*

Skala finansowa

1. Koronawirus (COVID-19) pogorszył moją sytuację finansową.
2. Straciłem/am dochód związany z pracą z powodu koronawirusa (COVID-19).
3. Koronawirus (COVID-19) wcale NIE wpłynął na moją sytuację finansową. *

Skala zasobów

1. Trudno mi było zdobyć potrzebne zasoby (żywność, papier toaletowy) ze względu na koronawirusa (COVID-19).
2. Z powodu koronawirusa (COVID-19) trudno mi było zdobyć rzeczy, których potrzebuję.
3. Koronawirus (COVID-19) NIE wpłynął na moją możliwość uzyskania potrzebnych zasobów. *

Skala psychologiczna

1. Wpadłem/am w depresję z powodu koronawirusa (COVID-19).
2. Wybuch koronawirusa (COVID-19) negatywnie wpłynął na moje zdrowie psychiczne.
3. Pandemia koronawirusa (COVID-19) NIE sprawiła, że poczułem/am się gorzej niż wcześniej. *

*Kwestionariusz doświadczeń koronawirusa*

Skala osobistych diagnoz / objawów

1. U mnie zdiagnozowano koronawirusa (COVID-19).
2. Miałem/am objawy podobne do koronawirusa w pewnym momencie w ciągu ostatnich dwóch miesięcy.
3. Byłem/am chory/a na coś innego niż koronawirus w ciągu ostatnich dwóch miesięcy.

Skala odległości od innych

1. Znam kogoś, u którego zdiagnozowano koronawirusa (COVID-19).
2. Byłem/am w bliskiej odległości z osobą, u której zdiagnozowano koronawirusa (COVID-19).
3. Znam kogoś, kto miał objawy podobne do koronawirusa w ciągu ostatnich dwóch miesięcy.
4. Byłem/am w bliskiej odległości z kimś, kto miał objawy podobne do koronawirusa w ciągu ostatnich dwóch miesięcy.

Skala wiadomości

1. Oglądam wiele wiadomości na temat koronawirusa (COVID-19).
2. Celowo staram się NIE oglądać wiadomości na temat koronawirusa (COVID-19). *
3. Ogromny procent czasu spędzam, próbując znaleźć aktualizacje online lub w telewizji na temat koronawirusa (COVID-19).

**[BLINDED FOR REVIEW]**

**Participants**

A total of 301 participants were recruited to take part in the study using the online research platform Prolific, all participants were recruited from Prolific (<https://www.prolific.co/>) and were verified as resident in the United Kingdom. The mean age ± SD of participants was 18.16 ± 1.64. Of these 187 participants identified as female, 112 as male and 2 as non-binary. In terms of ethnicity 215 participants identified as White, 32 as South Asian, 18 as mixed or other ethnicity, 16 as East Asian, eight as South East Asian, six as Black, three as Middle Eastern, one as Latin American and two did not state an ethnicity. In terms of nationality 281 were British citizens, 17 were non-British and 3 preferred not to say. In terms of residency with the nations of the UK, 268 participants were resident in England, 23 in Scotland, 7 in Wales and 3 in Northern Ireland.

**Methods and Materials**

Participants were tested on the long form measures of the Perceived Coronavirus Threat Questionnaire, the Coronavirus Impacts Questionnaire, and the Coronavirus Experiences Questionnaire, due to the largely centralised nature of the UK participants complete a version of the Governmental Response to Coronavirus Questionnaire in which the phrase UK government replaced federal, state and city government in those questions.

Participants answered these scales in the context of a larger study in which they were first presented with a series of vignettes of male and females from White, Black, East Asian and South Asian ethnicities performing behaviours that either violated or conformed to government measures to combat coronavirus. They were then asked to judge whether they perceived the behaviour positively or negatively, the person positively or negatively, how likely they themselves were to perform the behaviour and how much they felt the behaviour violated government guidelines. After answering these questions for 16 different vignettes participants then rated how at risk from Coronavirus men and women of each ethnic group were. Following this they answered the Coronavirus measures along with other questionnaires including the Social Dominance Orientation scale (Pratto et al., 1994), the Belief in a Just World scale (Lucas et al., 2011), a measure of political attitudes including voting intention and support for Brexit and additional measures related to risk perception and exposure to coronavirus. These questionnaires were presented in a random order with the items within them randomised, however the four Social Psychology measures on Corona virus taken from Conway et al. (2020) were always presented in one block and were preceded by the instruction “In this part of the study we will ask you a series of questions about COVID-19 and your response to it.” All measures were collected using the online experiment platform Gorilla (<https://gorilla.sc/>) on the 7^th^ of June 2020.

**[BLINDED FOR REVIEW]**

**Participants**

In order to investigate Coronavirus’ effects on individuals, 296 participants completed our survey via online Qualtrics link. All of participants were from different regions of Turkey including metropoles and rural areas (mostly from İstanbul). Throughout the online survey link, participants first given consent and information forms prior to the study. Once participants approved the consent form, they were asked to answer basic demographic information. Based on this, we obtained the following information:

1. First, out of 296 participants, 226 of the participants were females (76.4%), whereas 68 of them were males (23%) and 2 of the participants chose the “Don’t want to specify / Other” option (0.7%).
2. Second, the mean age of the participants was 30.98 and almost half of the participants were high-school graduates (49.3%); followed by university graduates (37.8%), postgraduates (10.5%), primary school graduates (1.7%) and finally secondary school graduates (0.7%).
3. Third, 45.9% of the participants indicated that they were students while 28% of participants reported that they were working full-time, followed by participants who did not work with the rate of 17.9%. Lastly the lowest rate was participants who were working part-time with the rate of 8.1%.
4. Additionally, due to Coronavirus outbreak, 61.7% of the participants reported that they were working from home while 27.1% of the participants reported that they were keep working from their workplaces.
5. Lastly, when asked the monthly income, almost half of the participants indicated that their income was between 0-3000 Turkish Liras (49.3%), followed by 3001-6000 TL (28%), 6001-9000 TL (11.8%) and lastly, 9001-and more TL (10.8%).

**Procedure**

The online Qualtrics link, which included the study, was distributed through social media to the participants and it was threefold:

1. Consent form and demographic questions
2. Autobiographical memory questions regarding to psychological distance
3. Perceived Coronavirus Threat Questionnaire

The data was collected between 4 May-15 June 2020. During the data collection process, curfew was imposed on weekends in metropolitan cities such as İstanbul, Ankara, etc. (in which major part of the data came from). Also, participants were given directions as there is no right or wrong answers for these questions and to choose how they feel.

**Autobiographical Memory Questions**

In the second part of our study, we asked participants to remember 4 events that they experienced before the Coronavirus outbreak began in Wuhan. The first two of these memories were to be positive memories, and the last two of them were to be negative memories. We asked participants to indicate a) Whether this memory is personal/public, b) The date of the event, c) How long it has been they feel since this event (psychological distance question), d) Whether they remember this event from their own eyes or as a witness.

**Perceived Coronavirus Threat Questionnaire**

In the third part of our study, we used Perceived Coronavirus Threat Questionnaire **(**with all 6 items) to investigate the effects of one’s self-report of how threatened they felt regarding to Coronavirus. However, in our study, we used 6-point Likert Scale to enable participants answer the questionnaire and also added an attention check question in between the questions (e.g. please check the 5^th^ box if you are reading carefully). In the data, we eliminated the participants who did not check the 5^th^ box (32 participants).

**[BLINDED FOR REVIEW]**

The purpose of our study was to evaluate the psychosocial impact of COVID-19 in the Guinea population. We undertook a cross-sectional online survey among literate people (from secondary to university level). We used free E-surv online platform to send questionnaires to students, who were encouraged to pass the questionnaire to each other through social media (Facebook, WhatsApp, messenger) by using a snowball strategy. The study ran between May 1st and May 10th 2020. The study questionnaire was divided in four parts: Socio-Demographic information, adapted “Social Psychological Measurements of COVID-19”, Impact of Event Scale –Revised and Penn state worry questionnaire (PSWQ). Participation in the study was totally voluntary. Institutional approval was obtained from [BLINDED FOR REVIEW] committee of research. Duplicated responses were carefully handled by blocking the same IP responses.

**[BLINDED FOR REVIEW]**

The present study aimed to examine the relationship between mental toughness traits and self-reported states of mental health (anxiety, stress, and depression) and wellbeing. In addition, the study aimed to examine whether resultant changes in individuals’ employment statuses (e.g. furloughing and working from home) had any effect on their mental health and wellbeing.

**Samples**

Two separate sampling procedures were used within our project to see whether the findings could be replicated using both a UK and non-UK sample.

*Sample A.* Data from the first sample (*Sample A*) were collected through an online survey platform (*Qualtrics*). An anonymous link to the survey was disseminated by the research team through social media and online internet groups. Three-hundred and seventy-one responses were collected; however, the COVID-19 SF had been incorporated into the survey mid-way through the data collection process. As a result, only 96 responses containing the COVID-19 SF items were collected between 30^th^ April and 22^nd^ May. The majority of the sample (*n* = 89) were from the UK.

*Sample B.* Data from the second sample (*Sample B*) were collected through an online survey platform (*Qualtrics*). However, the survey link was disseminated using *Amazon* *MTurk*, a crowdsourcing website that allowed the current researchers to purchase responses (respondents were paid $.30 for participation). In total, 397 complete responses were recorded between the 18^th^-25^th^ May (the majority of responses were collected on the 25^th^). The preference filter was used to present the survey only to non-UK participants, however, several responses from individuals living in the UK were still recorded (these have not been removed as we believe they still may be of use for your study).

**Materials and Procedure**

Participants first answered demographic question, including questions about their employment status during the pandemic. They were then asked to complete the Mental Toughness Questionnaire 48-item (Clough, Earle, & Sewell, 2002) and the following mental wellbeing scales; *The State-Trait Anxiety Inventory* (Spielberger, 2010), The *Depression, Anxiety and Stress Scale – 21 items* (Lovibond & Lovibond, 1995)*, Ryff’s Psychological Well-Being Scale* (1989), and *The subjective happiness scale* (Lyubomirsky & Lepper,1999). Finally, participants were asked to answer a series of questions about their current attitudes towards the pandemic, using the COVID-19 SF questions (27 items) and an additional fourteen questions about their actions during the pandemic, designed by the current researchers. For the COVID19-SF items, participants were given the following instructions “please read through the following statements about your thoughts on the COVID19 pandemic and indicate how much each statement applies to you.”. They were then presented with the 27 statements and asked to indicate how much each item applied to them using a 7-point scale (1 = Not very true of me at all; 7 = Very true of me).

**[BLINDED FOR REVIEW]**

The purpose of the study was to examine the consumption of substances such as alcohol, tobacco, and other drugs during the first months of confinement, and to evaluate its relationship with stress and depressive symptoms.

**Participants.** The participants were people over 18 years of age, residing in Mexico who voluntarily agreed and gave consent to respond to the questionnaire. The final sample includes 4,127 individuals, mainly women (n = 71.9%), aged between 21 and 40 years (55.9%). More than half (52.7%) have bachelor's degrees.

**Instruments.** The data was collected through an electronic questionnaire made up of different sections:

Sociodemographic data. It consists of 10 questions that inquire about sex, age, education, marital status, occupation, state of origin, and family income.

Family. Six questions about the family structure.

Perception of health status. Six questions that evaluate the general appreciation of the health condition in the last 12 months.

Coronavirus impact questionnaire (short version translated into Spanish, Conway, Woodard & Zubrod, 2020).

Coronavirus experiences questionnaire (short version translated into Spanish, Conway, Woodard & Zubrod, 2020).

Perceived coronavirus threat questionnaire (short version translated into Spanish, Conway, Woodard, & Zubrod, 2020).

Preventive measures. Checklist of eight actions to prevent the spread of Coronavirus.

Social distancing. Level of isolation that participants have sought in confinement.

Life and stress questionnaire. Purpose-built instrument

Patient Health Questionnaire 2 (PHQ-2). (Kroenke, 2003).

Substance use. Adaptation of ASSIST, (Humeniuk et al., 2008)

Emotional state. List of positive and negative emotions that participants might experience during the quarantine.

Support needs. Perceived support needs related to mental health and substance use as well as the use of telephone or web-based care services during the quarantine

**Procedure:** The questionnaire was administered through the Google Forms platform during May and June 2020. The invitation to respond to the questionnaire was disseminated through social networks such as Facebook, WhatsApp and Twitter

**[BLINDED FOR REVIEW]**

**Participants**

A convenient sample of emerging adults (i.e., people aged 18-30 years) was obtained using a variety of recruitment techniques, such as university and student mailing lists, posts on social media and relevant social media groups, researchers own participant pools, snowball recruiting initiated through colleagues, students and acquaintances who were asked to forward the link of the survey to emerging adults they know. A small percentage of the participants were recruited using Amazon Mturk in the US (0.9% of the entire sample).

The final sample included 2282 participants from six participating countries, i.e. China (n = 337), Italy (n = 443), Lithuania (n = 447), Portugal (n = 338), Slovenia (n = 369), and the U.S.A (n = 348). The participants ranged in age from 18 to 30 years (M = 23.5, SD = 3.48). Most of the participants were females (76.2%), while the remaining were males (22.8%) or gender queer individuals (1.0%).

**Procedure**

Researchers in each country firstly secured the approval of the Institutional Review Board at their home institution before data collection.

Once obtained the approval, the survey was initially developed in English. Then, all of the measures that were unavailable in target languages were translated by the authors of this paper and their local collaborators.

Participants first provided consent and then completed a 15-minute online survey in Qualtrics. We applied the survey between July and September 2020, when the strict protective measures (e.g., lockdown) were eased in most of the participating countries after the first wave of COVID-19 infections.

**Measures**

The online survey consisted of a series of demographic variables (e.g., gender, age) and psychometric questionnaires. Two questionnaires were adopted in the current study:

**Short version of Coronavirus Impacts Questionnaire.** Participants rated how true each item was for them on a 5-point Likert-type scale ranging from 1 (completely not true) to 7 (completely true) (αs = .80, .82, and .89 for the Financial, the Resource, and the Psychological scale respectively).

**Short version of Coronavirus Experiences Questionnaire.** Participants rated how true each item was for them on a 5-point Likert-type scale ranging from 1 (completely not true) to 7 (completely true) (αs = .50, .72, and .81 for the Personal diagnoses/symptoms, the Proximity to other, and the News scale respectively).

Both scales were translated in five different languages and these translation are here reported.

**Chinese version**

**Short version of Coronavirus Impacts Questionnaire**

***Financial Impact***

新冠肺炎给我的经济带来了负面的影响

由于新冠肺炎，我的工作收入有所降低

***Resource Impact***

由于新冠肺炎，我度过了一段无法获得必备资源（食物，卫生纸等）的困难时期

由于新冠肺炎，我很难获得我需要的东西

***Psychological Impact***

新冠肺炎让我变得抑郁

新冠肺炎的爆发给我的心理健康带来了负面的影响

**Short version of Coronavirus Experiences Questionnaire**

***Personal diagnoses/symptoms***

我被诊断感染了新冠肺炎

在过去的几个月内，我有和新冠肺炎相似的症状

在过去的几个月内，我患了新冠肺炎以外的其他疾病

***Proximity to others***

我与新冠肺炎患者有过密切接触

我与有新冠肺炎类似症状的人有过密切接触

***News***

我看了很多新冠肺炎的新闻

我花了大量的时间尝试在网上或电视上寻找新冠肺炎相关的最新报道

**Italian version**

**Short version of Coronavirus Impacts Questionnaire**

***Financial Impact***

Il Coronavirus ha avuto su di me un impatto negativo dal punto di vista finanziario

Ho perso parte delle mie entrare economiche a causa del Coronavirus

***Resource Impact***

Ho avuto difficoltà a recuperare risorse delle quali avevo bisogno (es. cibo, carta igienica) a causa del Coronavirus

A causa del Coronavirus, è stato difficile per me ottenere delle cose di cui avevo bisogno.

***Psychological Impact***

Sono diventato depresso a causa del Coronavirus.

La diffusione del Coronavirus ha avuto un impatto negativo sulla mia salute psicologica

**Short version of Coronavirus Experiences Questionnaire**

***Personal diagnoses/symptoms***

Mi è stato diagnosticato il Coronavirus

Negli ultimi mesi, per un periodo io ho avuto dei sintomi simili a quelli del Coronavrius

Negli ultimi mesi, sono stato malato ma non si trattava di Coronavirus

***Proximity to others***

Sono stato molto vicino a persone a cui hanno diagnosticato il Coronavirus

Sono stato molto vicino a persone che avevano sintomi simili a quelli del Coronavirus

***News***

Guardo molte news relative al Coronavirus

Passo una larga parte del mio tempo cercando aggiornamenti online o in TV relativi al Coronavirus

**Lithuanian version**

**Short version of Coronavirus Impacts Questionnaire**

***Financial Impact***

Koronavirusas neigiamai paveikė mano finansinę situaciją.

Dėl koronaviruso sumažėjo mano pajamos iš darbinės veiklos.

***Resource Impact***

Dėl koronaviruso man buvo itin sunku gauti kai kurių produktų (pvz., maisto ar tualetinio popieriaus).

Dėl koronaviruso man buvo itin sunku gauti man reikalingų dalykų.

***Psychological Impact***

Dėl koronaviruso aš tapau labiau prislėgtas.

Koronaviruso paplitimas neigiamai paveikė mano psichologinę sveikatą.

**Short version of Coronavirus Experiences Questionnaire**

***Personal diagnoses/symptoms***

Man buvo nustatytas koronavirusas .

Per pastaruosius kelis mėnesius man pasireiškė koronavirusui būdingi simptomai.

Per pastaruosius kelis mėnesius aš sirgau kita nei koronoviruso liga.

***Proximity to others***

Per pastaruosius kelis mėnesius turėjau artimą kontaktą su tuo, kam buvo nustatytas koronavirusas.

Turėjau artimą kontaktą su tuo, kam reiškėsi koronavirusui būdingi simptomai.

***News***

Žiūriu daug naujienų apie koronavirusą .

Skiriu daug laiko naujausių žinių apie koronavirusą peržiūrai internete arba per televiziją.

**Portuguese version**

**Short version of Coronavirus Impacts Questionnaire**

***Financial Impact***

O coronavírus tem-me afectado negativamente de um ponto de vista financeiro

Perdi rendimentos do meu trabalho devido ao coronavírus

***Resource Impact***

Tenho tido dificuldades em adquirir bens necessários (alimentação, papel higiénico) devido ao coronavírus

Tem sido difícil para mim adquirir as coisas que eu preciso devido ao coronavírus

***Psychological Impact***

Fiquei deprimido por causa do coronavírus

O surto de coronavírus tem tido um impacto negativo na minha saúde mental

**Short version of Coronavirus Experiences Questionnaire**

***Personal diagnoses/symptoms***

Fui diagnosticado com coronavírus

Tive sintomas que podiam estar associados ao coronavírus em algum momento nos últimos meses

Estive doente com algo não relacionado com o coronavírus nos últimos meses

***Proximity to others***

Estive em contacto próximo com alguém que foi diagnostico com coronavírus

Estive em contacto próximo com alguém que teve sintomas que podiam estar associados ao coronavírus nos últimos meses

***News***

Vejo muitas notícias sobre o coronavírus

Passo uma boa parte do meu tempo online ou a ver televisão a tentar actualizar-me sobre o coronavírus

**Slovenian version**

**Short version of Coronavirus Impacts Questionnaire**

***Financial Impact***

Koronavirus je negativno vplival na moje finančno stanje.

Zaradi koronavirusa sem izgubil s službo povezan dohodek.

***Resource Impact***

Zaradi koronavirusa sem imel težave z nabavo pomembnih dobrin (npr. hrana, toaletne potrebščine).

Zaradi koronavirusa je bilo težko dobiti stvari, ki sem jih potreboval.

***Psychological Impact***

Zaradi koronavirusa sem postal/-a depresiven/depresivna.

Izbruh koronavirusa je negativno vplival na moje duševno zdravje.

**Short version of Coronavirus Experiences Questionnaire**

***Personal diagnoses/symptoms***

Potrdili so mi okužbo s koronavirusom.

V preteklih mesecih sem imel simptome koronavirusne bolezni.

V preteklih mesecih sem zbolel, a ne za koronavirusno boleznijo.

***Proximity to others***

Bil sem v neposrednem stiku z nekom, ki so mu potrdili okužbo s koronavirusom.

Bil sem v neposrednem stiku z nekom, ki je imel simptome koronavirusne bolezni.

***News***

Pogosto spremljam novice o koronavirusu.

Velik del svojega časa iščem zadnje novice o koronavirusu na spletu ali na televiziji.

**[BLINDED FOR REVIEW]**

**Procedure**

These data were part of a longitudinal study to assess the effects of the coronavirus (COVID-19) pandemic (declared on 11 March 2020 by the World Health Organization) on the careers, household responsibilities, and wellbeing of masters- and doctoral-level academics and doctoral and PhD students. Academics who were teaching or conducting research at a higher education institution or who were postdoctoral fellows or doctoral or PhD students were eligible to participate in this study. Recruitment occurred globally via electronic advertisements posted on social media (e.g., Facebook, LinkedIn, Twitter) and professional listservs, emails sent by study investigators to publicly available email addresses, and participant forwarding of the survey link.

Baseline recruitment occurred over three weeks in April and May 2020. At the conclusion of that survey, participants were given the option to provide an email address where subsequent survey invitations could be sent. Data in the present study were from the first follow-up survey, which was administered between September 22, 2020 and October 14, 2020. Surveys were administered on Qualtrics. Study procedures were approved by human research ethics committees at [BLINDED FOR REVIEW].

**Measures**

Survey questions were derived from a larger battery of measures administered in the first follow-up survey in this longitudinal study. Demographic details included age, gender, and country. We also administered the Perceived Coronavirus Threat Questionnaire (Short; Conway, Woodard, & Zubrod, 2020), a 3-item measure that assesses perceived stress associated with COVID-19 on a 7-point Likert scale from 1 (*Not at all true of me*) to 7 (*Very true of me*); Cronbach’s alpha in the current study was good (α = .88).

**Participants**

Participants included 436 academics and doctoral/PhD students from 25 countries with an average age of 39.4 years (*SD =* 8.6, range 23 to 70 years). Participants were predominantly cis women (81.4%), with fewer participants who were cis men (14.2%); agender, genderqueer, gender fluid, non-binary, or trans men or women (*n =* 3.2%); or participants who declined to report their gender (1.2%). Most participants resided in the US (69.6%), Australia (12.1%), and Canada (5.3%; Table 1).

**Table 1.** Participants by Country of Current Residence (*N =* 431)

| Country of residence | *N* | % |
| --- | --- | --- |
| Australia | 52 | 12.1 |
| Canada | 23 | 5.3 |
| United States of America | 300 | 69.6 |
| Other ^a^ | 56 | 13.0 |

^a^ Includes all countries with fewer than 20 participants: Belgium, Chile, Colombia, Czech Republic, El Salvador, Finland, Georgia, Germany, India, Ireland, Israel, Italy, Malaysia, Netherlands, New Zealand, Norway, Paraguay, Poland, South Africa, Sweden, Switzerland, United Kingdom

**[BLINDED FOR REVIEW]**

**Participants**

The study involved 261 Filipinos (75.5% females; males: 24.5%) aged between 18 and 27 years. Most of the participants are currently studying (90%) while the rest are employed or unemployed at the time of data collection. All participants are single/never married.

**Procedure**

Participants were contacted through the university e-mail and social network. They were asked to complete an online questionnaire that includes demographic questions, their current perceptions, attitudes, responses, and experiences related to COVID-19, their need for cognition, and their level of existential anxiety. The data collection period ran from January 28 to March 6, 2021.

Measures

Participants completed the long versions of the following questionnaires developed by Conway, Woodard and Zubrod (2020):

·         Perceived Coronavirus Threat Questionnaire

·         State Governmental Response to Coronavirus Questionnaire

·         Coronavirus Impacts Questionnaire

·         Coronavirus Experiences Questionnaire

The data were collected as part of a descriptive study on Filipinos’ perceptions and experiences related to COVID-19 , their need for cognition and level of existential anxiety.

**References**

BBC (2020), Koronavirüs: Adım adım Türkiye'nin Covid-19'la mücadelesi,

<https://www.bbc.com/turkce/haberler-turkiye-52899914>, (Accessed: 10.10.2020).

Clark, L. A., & Watson, D. (2019). Constructing validity: New developments in creating objective measuring instruments. *Psychological Assessment, 31*(12), 1412–1427.

Clough, P., Earle, K., & Sewell, D. (2002). Mental toughness: The concept and its measurement. *Solutions in sport psychology*, 32-43.

Comrey, A. L., & Lee, H. B. (1992). *A first course in factor analysis* (2nd ed.). Lawrence Erlbaum Associates, Inc.

Kennedy, R., Clifford, S., Burleigh, T., Jewell, R., & Waggoner, P. (2018). The shape of and solutions to the MTurk quality crisis. *Available at SSRN 3272468*.

Kurdi, B., Lozano, S., & Banaji, M. R. (2017). Introducing the Open Affective Standardized

Image Set (OASIS). Behavior Research Methods, 49(2), 457–470. https://doi.org/10.3758/s13428-016-0715-3

Lai, K., & Green, S. B. (2016) The problem with having two watches: Assessment of fit when RMSEA and CFI disagree. *Multivariate Behavioral Research, 51*(2-3), 220-239. doi: 10.1080/00273171.2015.1134306

Li, C.-H. (2016). The performance of ML, DWLS, and ULS estimation with robust corrections in structural equation models with ordinal variables. *Psychological Methods, 21*(3), 369-387. Retrieved from https://psycnet.apa.org/record/2016-41158-001

Lovibond, S.H. & Lovibond, P.F. (1995). *Manual for the Depression Anxiety Stress Scales*. (2nd. Ed.)  Sydney: Psychology Foundation

Lubben, J., Blozik, E., Gillmann, G., Iliffe, S., von Renteln Kruse, W., Beck, J. C., & Stuck, A.

E. (2006). Performance of an abbreviated version of the Lubben Social Network Scale among three European community-dwelling older adult populations. The Gerontologist, 46(4), 503–513. https://doi.org/10.1093/geront/46.4.503

Lucas, T., Zhdanova, L., & Alexander, S. (2011). Procedural and Distributive Justice Beliefs for Self and Others: Assessment of a Four-Factor Individual Differences Model. *Journal of Individual Differences*, *32*(1), 14–25. https://doi.org/10.1027/1614-0001/a000032

Lyubomirsky, S., & Lepper, H. S. (1999). A measure of subjective happiness: Preliminary reliability and construct validation. Social Indicators Research, 46, 137-155.

Massaccesi C, Chiappini E, Paracampo R and Korb S (2021) Large Gatherings? No, Thank You. Devaluation of Crowded Social Scenes During the COVID-19 Pandemic. Front. Psychol. 12:689162. doi: 10.3389/fpsyg.2021.689162

Ministry of the Interior (19.03.2020). <https://www.icisleri.gov.tr/koronavirus-tedbirleri->

genelgesi-kapsaminda-149382-is-yeri-gecici-sureligine-faaliyetlerine-ara-verdi

(Accessed: 11.10.2020).

Pratto, F., Sidanius, J., Stallworth, L. M., & Malle, B. F. (1994). Social dominance orientation: a personality variable predicting social and political attitudes. *Journal of Personality and Social Psychology*, *67*(4), 741–763. https://doi.org/10.1037/0022-3514.67.4.741

Rammstedt, B., & John, O. P. (2007). Measuring personality in one minute or less: A 10-item

short version of the Big Five Inventory in English and German. Journal of Research in Personality, 41(1), 203–212. https://doi.org/10.1016/j.jrp.2006.02.001

Republic of Turkey Ministry (2020a). Covid-19 Information Page, General Coronavirus Table.

<https://covid19.saglik.gov.tr/EN-69532/general-coronavirus-table.html> (Accessed: 12.10.2020).

Republic of Turkey Ministry (2020b). COVID-19 Weekly Situation Report,

<https://covid19.saglik.gov.tr/Eklenti/39040/0/covid-19-weekly-situation-report---40-weekpdf.pdf?_tag1=EB07E7F16F13B11C75B38F3C1F4EEBFC0A2232C7> (Accessed: 11.10.2020).

Russell, D. W. (1996). UCLA Loneliness Scale (Version 3): Reliability, Validity, and Factor

Structure. Journal of Personality Assessment, 66(1), 20–40. https://doi.org/10.1207/s15327752jpa6601_2

Ryff, C. D. (1989). Happiness is everything, or is it? Explorations on the meaning of psychological well-being. *Journal of personality and social psychology*, *57*(6), 1069.

Schreiber, J. B., Stage, F. K., King, J., Nora, A., & Barlow, E. A. (2006). Reporting structural equation modeling and confirmatory factor analysis results: A review. *The Journal of Educational Research, 99*(6), 323-337. <https://doi.org/10.3200/JOER.99.6.323-338>

Spielberger, C. D. (2010). State‐Trait anxiety inventory. *The Corsini encyclopedia of psychology*, 1-1.

UCLA: Statistical Consulting Group (n.d.). *Confirmatory factor analysis (CFA) in R with Lavaan.* <https://stats.idre.ucla.edu/r/seminars/rcfa/#s2b>

Watson, D., Clark, L. A., & Tellegen, A. (1988). Development and validation of brief measures

of positive and negative affect: The PANAS scales. J Pers Soc Psychol, 54(6), 1063–1070.

Young, A. G., & Pearce, S. (2013). A beginner’s guide to factor analysis: Focusing on Exploratory Factor Analysis. *Tutorials in Quantitative Methods for Psychology, 9*(2), 79-94. <https://doi.org/10.20982/tqmp.09.2.p079>

1. In all studies, participants completed additional questionnaires related to the theoretical aims of each specific project; for the sake of brevity, we focus exclusively on the questionnaires central to the social psychological questions about COVID-19 discussed in the present manuscript. [↑](#footnote-ref-1)
2. Combining Samples 1-3, our sample had typical U.S. Mechanical Turk characteristics for age (*mean* = 41), biological sex assigned at birth (48% female), and race/ethnicity (largest groups were White/European-American = 78%, Asian = 9%, and Black/African-American = 7%). [↑](#footnote-ref-2)
3. Due to time constraints, we were not able to administer all three versions of the scale (Federal, State, and City). We instructed participants to answer the questions as they pertained to their perceptions of their *local* government. With this exception, we respected the wording of the instructions of the original scales. [↑](#footnote-ref-3)
